# Study of the Metabolic Profiles of “Indazole-3-Carboxamide” and “Isatin Acyl Hydrazone” (OXIZID) Synthetic Cannabinoids in a Human Liver Microsome Model Using UHPLC-QE Orbitrap MS

**DOI:** 10.3390/metabo13040576

**Published:** 2023-04-18

**Authors:** Jiahong Xiang, Di Wen, Junbo Zhao, Ping Xiang, Yan Shi, Chunling Ma

**Affiliations:** 1 Hebei Key Laboratory of Forensic Medicine, Collaborative Innovation Center of Forensic Medical Molecular Identification, Research Unit of Digestive Tract Microecosystem Pharmacology and Toxicology, College of Forensic Medicine, Hebei Medical University, Chinese Academy of Medical Sciences, Shijiazhuang 050017, China; 2 Shanghai Key Laboratory of Forensic Medicine, Shanghai Forensic Science Platform, Key Laboratory of Judicial Expertise, Department of Forensic Toxicology, Academy of Forensic Science, Ministry of Justice, Shanghai 200063, China

**Keywords:** metabolism, pooled human liver microsome, UHPLC-QE Orbitrap MS, NPS, OXIZIDs, synthetic cannabinoid

## Abstract

Unregulated core structures, “isatin acyl hydrazones” (OXIZIDs), have quietly appeared on the market since China legislated to ban seven general core scaffolds of synthetic cannabinoids (SCs). The fast evolution of SCs presents clinical and forensic toxicologists with challenges. Due to extensive metabolism, the parent compounds are barely detectable in urine. Therefore, studies on the metabolism of SCs are essential to facilitate their detection in biological matrices. The aim of the present study was to elucidate the metabolism of two cores, “indazole-3-carboxamide” (e.g., ADB-BUTINACA) and “isatin acyl hydrazone” (e.g., BZO-HEXOXIZID). The in vitro phase I and phase II metabolism of these six SCs was investigated by incubating 10 mg/mL pooled human liver microsomes with co-substrates for 3 h at 37 °C, and then analyzing the reaction mixture using ultrahigh-performance liquid chromatography-quadrupole/electrostatic field orbitrap mass spectrometry. In total, 9 to 34 metabolites were detected for each SC, and the major biotransformations were hydroxylation, dihydrodiol formation (MDMB-4en-PINACA and BZO-4en-POXIZID), oxidative defluorination (5-fluoro BZO-POXIZID), hydrogenation, hydrolysis, dehydrogenation, oxidate transformation to ketone and carboxylate, N-dealkylation, and glucuronidation. Comparing our results with previous studies, the parent drugs and SC metabolites formed via hydrogenation, carboxylation, ketone formation, and oxidative defluorination were identified as suitable biomarkers.

## 1. Introduction

Reports published by the United Nations Office on Drugs and Crime indicate that the number of new psychoactive substances (NPS) found on global drug markets has now stabilized at around 550 per year, confirming that their abuse is becoming a difficult drug issue worldwide [1]. Among the NPS available globally, synthetic cannabinoids (SCs) remain one of the most prevalent classes of NPS [2]. The SCs mimic and surpass the effects of (-)-trans-Δ9-tetrahydrocannabinol (THC), and most of them have high potency and affinity for the human cannabinoid receptor subtype-1 (CB1) and subtype-2 (CB2) [3,4].

Many SCs have been associated with psychosis, hallucinations, seizures, respiratory failure, cardiovascular effects, coma, and even death [5,6,7]. Various countries, including China, now enforce strict legal controls over SCs. Indeed, in July 2021, China put into effect a new generic legislation banning SCs containing one of seven general core scaffolds. However, these regulations now appear to have driven manufacturers to synthesize SCs with alternative core structures. This trend is particularly evident by the recent emergence of OXIZIDs, a new class of NPS that has emerged in the recreational drug market in several countries, particularly the United States, China, and Singapore [8].

**Figure 1:**
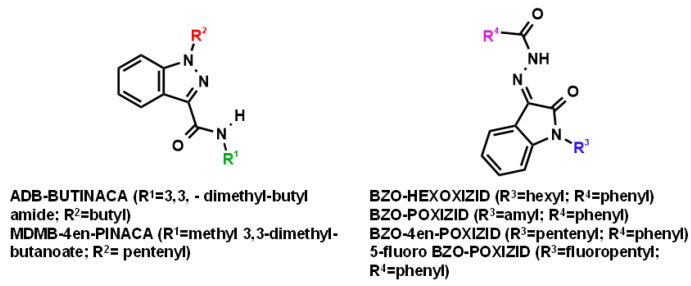
Molecular structures of the studied synthetic cannabinoids.

The introduction of OXIZIDs as a new class of non-scheduled compounds has led to the anticipation of an increasing number of OXIZID-type substances in the near future [9]. The present study concentrates on four specific OXIZIDs, namely BZO-HEXOXIZID, BZO-POXIZID, BZO-4en-POXIZID, and 5-fluoro BZO-POXIZID, which belong to the N-alkyl isatin acyl hydrazone structural class of compounds due to their feature “isatin acyl hydrazone” core. This core differs from the general SC structures regulated in revised Chinese legislation, thereby sparing OXIZIDs from legal control. The two other substances, ADB-BUTINACA and MDMB-4en-PINACA, have an “indazole-3-carboxamide” core, are under Chinese legal control, and may also be showing a popular trend in abuse in China, according to the research carried out by Zhou et al. (Figure 1) [10].

Confirmation of the consumption of NPS, including SCs, requires the development of targeted methods for those compounds. Moreover, due to the limited half-life of NPS in blood and serum and their extensive metabolism, the proof of consumption often relies on the detection of metabolites in urine, a matrix characterized by a wider detection window [11,12]. In fact, SCs undergo such extensive biotransformation after administration that little to no unchanged parent drug is found in human urine. Certain SC metabolites, such as monohydroxylated species, have been reported to retain some biological activity and to cross the blood–brain barrier and bind to the CB1 and CB2 receptors [13,14,15]. Hydroxylated metabolites have been suggested for use as in vivo markers to identify several SCs in clinical and forensic urine samples. Therefore, metabolic studies are critical for confirming SC substance use.

Due to the limited access to samples from subjects with known or suspected drug intake, as well as uncertainties related to which drug has been ingested, in vitro model systems of drug metabolism have emerged as a valuable tool. Several studies have shown comparable metabolic profiles between in vitro human liver microsome (HLM) or human hepatocyte assays and human in vivo urine samples [16,17,18,19]. Previous studies have examined the metabolic profiles of ADB-BUTINACA and MDMB-4en-PINACA after in vitro or in vivo incubation, and some differences have been reported between them [20,21,22,23,24]. The metabolic profiles of BZO-HEXOXIZID, BZO-POXIZID, and 5-fluoro BZO-POXIZID have been explored, with 12–16 metabolites reported for each; however, none have been reported for BZO-4en-POXIZID [25].

The aim of the present study was to elucidate the main in vitro phase I and phase II metabolites of ADB-BUTINACA, MDMB-4en-PINACA, BZO-HEXOXIZID, BZO-POXIZID, 5-fluoro BZO-POXIZID, and BZO-4en-POXIZID, with a focus on the analytical differentiation and interpretation of the metabolites and the identification of major-specific biomarkers for intake. A further aim was to explore the metabolic rules of the “isatin acyl hydrazone” and “indazole-3-carboxamide” cores (Figure 1). The structures of the six SCs and their metabolites were determined using ultrahigh-performance liquid chromatography-quadrupole/electrostatic field orbitrap mass spectrometry (UHPLC-QE Orbitrap MS).

## 2. Materials and Methods

### 2.1. Chemicals and Reagents

ADB-BUTINACA [N-(1-amino-3,3-dimethyl-1-oxobutan-2-yl)−1-butyl-1H-indazole-3-carboxamide] (≥98%), MDMB-4en-PINACA [methyl 3,3-dimethyl-2-(1-(pent-4-en-1-yl)−1H-indazole-3-carboxamido) butanoate] (≥98%), BZO-HEXOXIZID [(Z)-N′-(1-hexyl-2-oxoindolin-3ylidene) benzohydrazide] (≥95%), BZO-POXIZID [(Z)-N′-(1-pentyl-2-oxoindolin-3-ylidene) benzohydrazide] (≥98%), 5F-BZO-POXIZID [(Z)-N′-(1-(5-fluoropentyl)-2-oxoindolin-3-ylidene) benzohydrazide] (≥98%), and BZO-4en-POXIZID [(Z)-N’-(2-oxo-1-(pent-4-en-1-yl) indolin-3-ylidene) benzo hydrazide] (≥95%) were purchased from Cayman Chemical Company (Ann Arbor, MI, USA). Analytical grade methanol and acetonitrile (>99%) were purchased from Sigma-Aldrich (St. Louis, MO, USA), and formic acid (FA, analytical grade, 98%) was purchased from Fluka (Buchs, Switzerland). Ammonium formate (>99%) was obtained from Sigma-Aldrich (St. Louis, MO, USA). The 0.1 mol/L phosphate-buffered saline (pH = 7.4), pooled human liver microsomes (pHLMs, protein concentration of 20 mg/mL), nicotinamide adenine dinucleotide phosphate (NADPH) regeneration solution A (1.3 mmol/L NADP+, 3.3 mmol/L magnesium chloride, and 3.3 mmol/L glucose-6-phosphate), NADPH regeneration solution B (0.4 U/mL 6-phosphoglucose dehydrogenase and 0.05 mmol/L sodium citrate), and the UGT incubation system (uridine diphosphate glucuronic acid [UDPGA], D-gluconic acid-1,4-lactone, and alamethicin) were purchased from the Huizhi Taikang Pharmaceutical. Technology Co. (Beijing, China). A Millipore AFS-10 water purification system (Billerical, MA, USA) was used to prepare ultrapure water for samples. All analytical compounds were dissolved in methanol at 1 mg/mL concentrations as working solutions.

### 2.2. Incubation in pHLMs

A 2 μL aliquot of working solution (1 mg/mL) was mixed with 176 μL of phosphate-buffered saline and 10 μL of liver microsomal solution and then pre-incubated for 5 min at 37 °C. The metabolic reaction was initiated by adding 10 μL NADPH regeneration solution A and 2 μL NADPH regeneration solution B. The reaction was allowed to proceed at a constant temperature of 37 °C for 120 min, followed by the addition of 30 μL UGT incubation system reagents (10 μL each of UDPGA, alamethicin, and D-gluconic acid-1,4-lactone). After 60 min, the reaction was terminated by protein precipitation by adding 200 μL ice-cold acetonitrile. The mixture was then centrifuged at 13,500× *g* for 5 min. The supernatant was evaporated to dryness under a stream of air in an MD200 sample concentrator (ALLSHENG, Hangzhou, China). The residues were reconstituted in 100 μL reagent (95% phase A and 5% phase B) for analysis with the UHPLC-QE-Orbitrap MS system. A blank sample without the six compounds and a negative control sample without pHLMs were also prepared, with the substrate or enzyme replaced with buffer.

### 2.3. LC-MS Analysis

The samples were separated and analyzed using an UHPLC-QE-Orbitrap MS system comprising a Thermo-Scientific™ Q-Exactive™ quadrupole electrostatic field orbitrap mass spectrometer coupled to an UltiMate 3000 ultrahigh-performance liquid chromatography (UHPLC) system (both from Thermo Fisher Scientific, Waltham, MA, USA) with a heated electrospray ionization (HESI)-II source in the positive ionization mode. The samples were separated with an Agilent Eclipse Plus C18 column (100 × 2.1 mm, 3.5 μm) maintained at 25 °C and a flow rate of 300 μL/min. The mobile phases were mobile phase A (water with 0.1% *v*/*v* formic acid) and mobile phase B (acetonitrile with 0.1% *v*/*v* formic acid). The following gradient was used: 0–1.0 min, 5% B; 1.0–11.0 min, 5% B–95% B; 11.0–15.5 min, 95% B; 15.5–16.0 min, 95% B–5% B; 16.0–18.0 min, 5% B. The injection volume was 10 μL.

The following HESI-II source conditions were adopted: capillary temperature, 325 °C; auxiliary gas heater temperature, 300 °C; spray voltage, 3.50 kV; auxiliary gas, 15 arbitrary units (AU); and sheath gas, 45 AU. The mass spectrometry analysis was carried out in full-scan (FS) mode to trigger data-dependent acquisition in tandem mass spectrometry (FS-ddMS2) mode. The FS data acquisition was performed with a survey scan from *m*/*z* 100 to 1000, and a maximum injection time (IT) of 250 ms. The fragment ions were detected from *m*/*z* 50 to 1000 with normalized collision energy (NCE) of 10, 15, and 20 eV. The maximum IT was set to 50 ms. The spectrum data type was centroid, and all mass spectral data were processed using Xcalibur Qual Browser software, version 3.1. The mass deviation allowed was within 5 ppm.

## 3. Results

**Figure 2:**
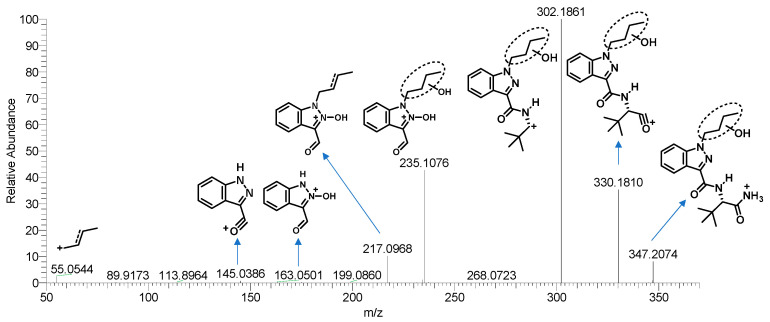
Mass spectrometry spectra and fragmentation of A1 (the monohydroxylated metabolite of ADB-BUTINACA, the wavy bonds position unassigned).

**Figure 3:**
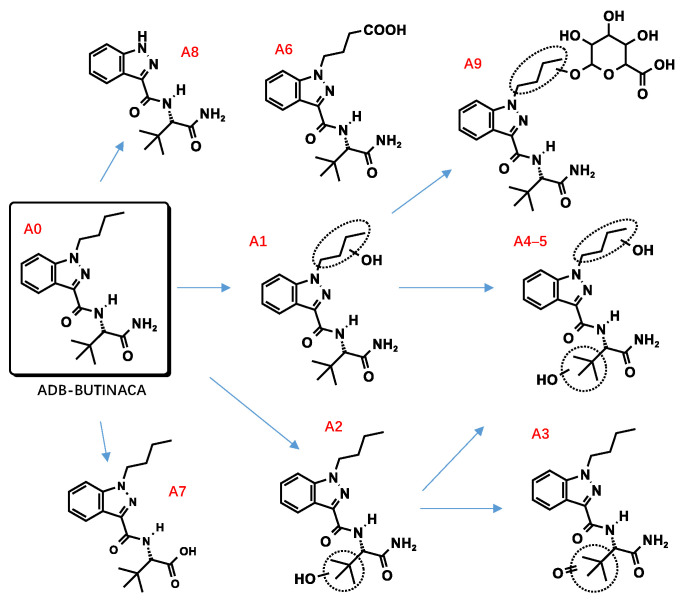
Proposed metabolic pathway of ADB-BUTINACA.

**Figure 4:**
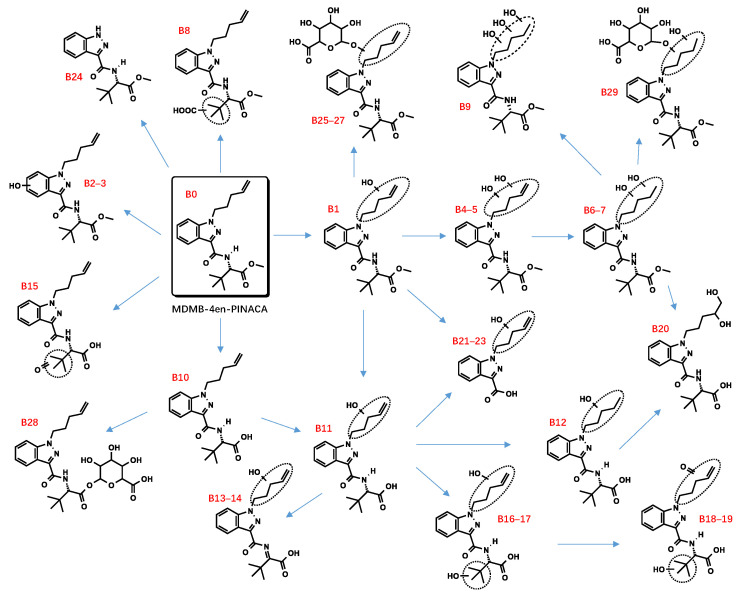
Proposed metabolic pathway of MDMB-4en-PINACA.

**Figure 5:**
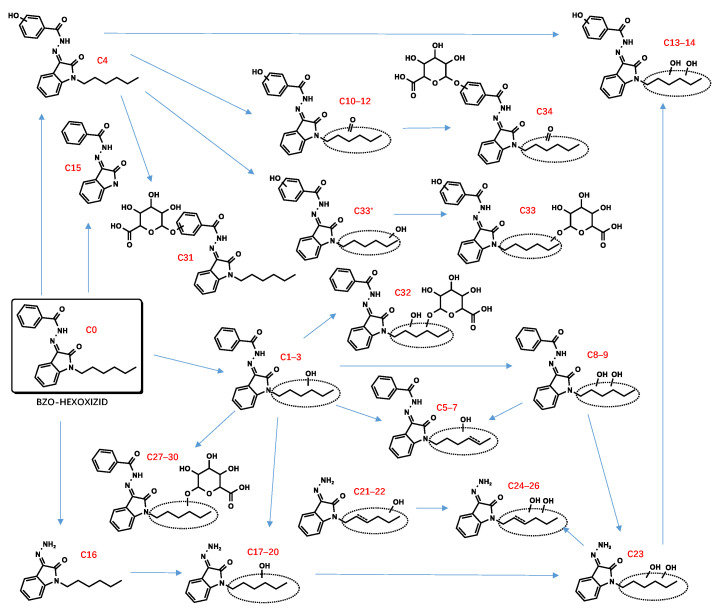
Proposed metabolic pathway of BZO-HEXOXIZID.

**Figure 6:**
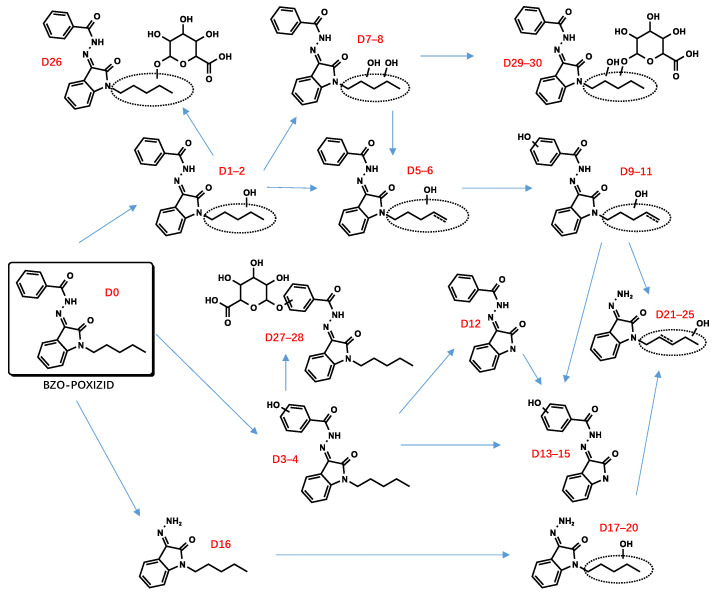
Proposed metabolic pathway of BZO-POXIZID.

**Figure 7:**
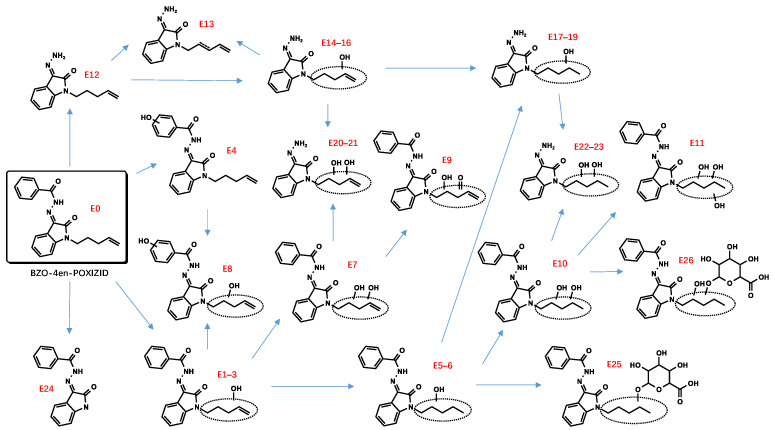
Proposed metabolic pathway of BZO-4en-POXIZID.

**Figure 8:**
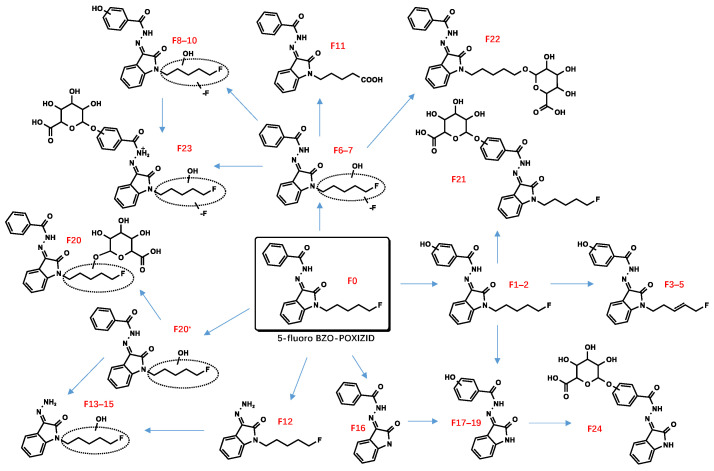
Proposed metabolic pathway of 5-fluoro BZO-POXIZID.

All metabolite structures were inferred based on their accurate molecular mass and MS/MS spectra. The interpretations of the mass spectra were based on product ions formed during collision-induced dissociation (CID). The extracted mass chromatograms and metabolites are provided in Supplementary Material (Figures S1–S6). The biotransformation, chemical formula, retention time, measured and exact masses of the protonated molecule, mass error, and peak area are shown in Supplementary Material (Tables S1–S6). The product ion mass spectrum and chemical structures of the studied synthetic cannabinoids and their identified metabolites are listed in Supplementary Material Figure S7–S12. Figure 2 shows the mass spectrum and fragment analysis of A1, a monohydroxylated metabolite of compound ADB-BUTINACA. The proposed metabolic pathways are provided in Figure 3, Figure 4, Figure 5, Figure 6, Figure 7 and Figure 8.

### 3.1. “Indazole-3-Carboxamide” Synthetic Cannabinoid Receptor Agonists (SCRAs)

#### 3.1.1. Parent Compounds

The LC-MS analysis confirmed that ADB-BUTINACA (*m*/*z* 331.2129) and MDMB-4en-PINACA (*m*/*z* 358.2125) were both “indazole-3-carboxamide” SCRAs. They both showed the same characteristic fragment ion at *m*/*z* 145.0398 for the indazole acylium cation, and the *m*/*z* 201.1025 and 213.1022 for the butyl/pentenyl indazole acylium cation. The ions at *m*/*z* 286.1916 and 298.1913 could be explained by alpha cleavage of ADB-BUTINACA and MDMB-4en-PINACA, respectively. In addition, the rearrangement reaction of the fragment ion of ADB-BUTINACA at *m*/*z* 219.1128 might be explained by an amide cleavage, followed by a nucleophilic attack of the oxygen at imidazole nitrogen in position 2, which is probably activated by the negative effect of the nitrogen in position 1, leading to a seventh ring [26]. The same reaction could explain the fragment ions of *m*/*z* 163.0499 and the fragment ions of MDMB-4en-PINACA (*m*/*z* 358.2125), *m*/*z* 231.1124 [27]. The fragment ions at *m*/*z* 69.0699 showed the pentenyl side chain of MDMB-4en-PINACA. These fragment ions were used as the basis for elucidating the metabolite structures.

#### 3.1.2. Phase I and II Metabolites

##### Hydroxylation

As with many indazole-3-carboxamide synthetic cannabinoids, the ADB-BUTINACA and MDMB-4en-PINACA metabolites were susceptible to hydroxylation, and the metabolites were integral multiples of 16 Da larger than the parent compounds. The hydroxylations of ADB-BUTINACA were mainly the monohydroxylation product of the butyl side chain (A1) and tert-butyl side chain (A2), and the dihydroxylation metabolites of both moieties (A4, 5). The product ion of A1 (*m*/*z* 347.2078) at *m*/*z* 217.0972 (loss of water from *m*/*z* 235.1077) and 235.1077 indicated that hydroxylation occurred at the N-butyl indazole moiety. The product ion at *m*/*z* 113.0597 of A2 (*m*/*z* 347.2078) indicated that hydroxylation occurred at the t-butyl moiety.

The hydroxylation of MDMB-4en-PINACA often occurred at the pentenyl (B1, B4, B5) and tert-butyl (B18, B19) side chains. The difference between the two “indazole-3-carboxamide” SCRAs was that no hydroxylation was found for ADB-BUTINACA at the benzene ring, whereas the metabolites of MDMB-4en-PINACA (B2, B3) did contain hydroxylations. The fragment ions of B1 (*m*/*z* 374.2074), at *m*/*z* 211.0866 and 67.0542, could be explained by a loss of water from a hydroxylated pentenyl side chain. The product ion of B2,3 (*m*/*z* 374.2074), at *m*/*z* 69.0699, suggested that hydroxylation did not occur at the pentenyl side chain. The product ion at *m*/*z* 229.0972 was 16 Da larger than the characteristic fragment ion at *m*/*z* 213.1022, indicating that hydroxylation occurred at the indazole ring. The product ion of B4,5 (*m*/*z* 390.2023) at *m*/*z* 101.0597 indicated that dihydroxylation occurred at the pentenyl side chain.

##### Dihydrodiol Formation

The pentenyl side chain of MDMB-4en-PINACA was susceptible to a classic reaction of carbon–carbon double bond oxidation. Further hydroxylation led to monohydroxylation (B12), dihydrodiol formation (B6, B7, and B20), and dihydrotriol formation (B9), with the dihydrodiol as the common metabolite of the pentenyl. The fragment ion at *m*/*z* 247.1075 of B6,7 (*m*/*z* 392.2180) was 2 Da larger than the ion at 245.0919 of B4, suggesting that a hydrogenation occurred at the pentenyl side chain. The fragment ion at *m*/*z* 245.0919 might be explained by a rearrangement, such as occurred for the ion at *m*/*z* 231.1124 of MDMB-4en-PINACA. Some literature reports indicate that this hydrogenation could only be generated by the oxidation of a double bond, leading to the formation of a hydroxyl functional group at the 5-position [27]. The positions of the other hydroxyl groups on the other pentyl side chains remain uncertain.

##### Hydrolysis

The hydrolysis of ADB-BUTINACA and MDMB-4en-PINACA was a terminal amide hydrolysis (A7, *m*/*z* 332.1969) and a terminal ester hydrolysis (B10, *m*/*z* 344.1969), resulting in carboxylic acid metabolites. The hydrolysis metabolites could be further hydroxylated to obtain B11, B16, and B17, or combined with hydrogenation to form B12 and B20. Apart from the characteristic ions at *m*/*z* 86.0964 (unchanged t-butyl moiety) and *m*/*z* 131.0601 (unchanged methyl-indazole), the ions at *m*/*z* 67.0542 (loss of water by *m*/*z* 85.0648, hydroxylated pentenyl side chain) could also assist in identifying the position of the hydroxyl group. Further hydrolysis reactions were also apparent. Although hydrolysis of the MDMB-4en-PINACA linked group amide bond was not detected in this study, monohydroxylated metabolites of MDMB-4en-PINACA bonded to the linked group amide hydrolysis (B21–23) were identified.

##### Dehydrogenation and Oxidate-to-Ketone and Carboxylate Conversions

Further dehydrogenation of hydroxylated metabolites could result in ketone metabolites, such as A3, B15, B18, and B19, and carboxylic acid metabolites A6 and B9, from terminal alkyl or alkenyl groups. For example, A3 (*m*/*z* 345.1921) was 2 Da smaller than A1 (*m*/*z* 347.2078). Excluding the unchanged butyl indazole acyl moiety (*m*/*z* 163.0502, 201.1022, and 219.1129), the *m*/*z* 300.1706 was 45 Da (CH3NO+, formamide) smaller than the parent ion (*m*/*z* 345.1921), indicating that the ketone occurred at the tert-butyl moiety. In addition, a C-N double bond metabolite was indicated (B13, B14).

##### N-Dealkylation

A8 (*m*/*z* 275.1503) and B24 (*m*/*z* 290.1499) were 56 Da (C4H8+) and 68(C5H8+) Da less than ADB-BUTINACA-BUTINACA and MDMB-4en-PINACA-4en-PINACA, respectively. Other characteristic fragments were consistent with the original drug.

##### Glucuronidation

The hepatocyte microsomes contain glucuronic transferase, which can transfer a glucuronic acid group to the hydroxyl, amino, and carboxyl groups of poisons or other active substances, forming a glucuronic side group, with a molecular weight increase of 176 Da. (A9 *m*/*z* 523.2399, B25–27 *m*/*z* 550.2395, B28 *m*/*z* 520.2290, B29 *m*/*z* 568.2501). The other characteristic fragment remains unchanged.

### 3.2. “Isatin Acyl Hydrazone” SCRAs

Incubation of BZO-HEXOXIZID, BZO-POXIZID, BZO-4en-POXIZID, and 5-fluoro BZO-POXIZID with pHLMs resulted in the identification of 34 (C1–C34), 30 (D1–D30), 26 (E1–E26), and 24 (F1–F24) phase I and II metabolites, respectively. The metabolites eluted between 5.10 and 11.67 min, with the parent drugs eluting at 11.67, 11.11, 10.53, and 10.01 min, respectively. The metabolites were formed via hydroxylation, dehydrogenation, hydrogenation, amido hydrolysis, N-dealkylation, oxidative defluorination, and glucuronidation, either alone or in combination.

#### 3.2.1. Parent Compounds

Four different substituents distinguished these four structurally similar compounds. The BZO-POXIZID ion (*m*/*z* 336.1707, hexyl) was 14 Da (CH4) smaller than the BZO-HEXOXIZID ion (*m*/*z* 350.1863, pentyl), whereas the BZO-4en-POXIZID ion (*m*/*z* 334.1550, pentenyl) was 2 Da smaller than the BZO-POXIZID ion (*m*/*z* 336.1707, pentyl). The 5-fluoro BZO-POXIZID ion (*m*/*z* 354.1612, fluoropentyl) contained a fluorine atom. The characteristic fragment ions were found at *m*/*z* 145.0396 for the oxoindoline structure formed by nitrogen–nitrogen bond cleavage, at *m*/*z* 105.0335 for the benzyl oxonium ion, and at *m*/*z* 77.0386 for the phenyl ion. These fragment ions were used as the basis for elucidating the metabolite structures.

#### 3.2.2. Phase I and II Metabolites

##### Hydroxylation

The alkyl side chain and the benzene ring of the benzoyl moiety were susceptible to hydroxylation, resulting in monohydroxylation (C1–4, D1–4, E1–4, F1–2), dihydroxylation (C8–9, C32*, D7–8, E7–8), and trihydroxylation (C13–14) metabolites. No hydroxylations were detected occurring at the isatin acyl hydrazone.

The hydroxylated metabolites were integral multiples of 16 Da larger than the parent compounds. The fragment ion at *m*/*z* 174.0662 resulted from the breaking of a carbon–carbon bond and showed an unchanged isatin acyl hydrazone ion. The product ion at *m*/*z* 121.0284, 16 Da larger than *m*/*z* 105.0335 for the benzyl oxonium ion, suggested that the hydroxylation occurred at the benzene ring of the benzoyl moiety.

##### Dehydrogenation and Oxidate-to-Ketone and Carboxylate Conversions

The metabolites (C5–7, C21–22, D5–6, D9–11, F3–5) indicated carbon–carbon double bonds. For example, the fragment ion at *m*/*z* 346.1550 of C5–7 (*m*/*z* 364.1656) was explained by a loss of water after hydroxylation and dehydrogenation of the hexyl side chain, confirming the existence of a carbon–carbon double bond. Ketone metabolites (C10–12, E9) were posited based on the accurate mass shift of +14 Da of each parent ion. The fragment ion at *m*/*z* 258.1237 was the diagnostic fragment for confirming ketone identities. Metabolites characterized by a mass shift of +30 Da were identified as either carboxylates or monohydroxylated metabolites plus a carbonyl group (C24–26). In synthetic organic chemistry, ketone and carboxylate could be formed from alcohol via an oxidation reaction with one or more steps. Similarly, ketone metabolites are generally formed from an alcohol via dehydrogenation, and carboxylates are formed often from CH3 terminal through 3 successive cytochrome P450 oxidations [28,29].

##### Defluorination

The metabolites F6 and F7 (*m*/*z* 352.1656) were 2 Da smaller than 5-fluoro BZO-POXIZID (*m*/*z* 354.1612). We speculated that F6 could possibly be a monohydroxylated metabolite at the N2/N3/N4 position, resulting from a combination of nonoxidative defluorination and hydroxylation. F7, with a high peak area, was likely to be the oxidative defluorinated metabolite and was further oxidized to carboxyl (F11). F8–10 were identified as combinations with the monohydroxylation at the benzene ring of the benzoyl group.

##### Dihydrodiol Formation

The pentenyl side chain of BZO-4en-POXIZID was susceptible to the classic double-bond oxidation reaction. After further hydroxylation, monohydroxylation (E5–6), dihydrodiol (E10), and dihydrotriol (E11) metabolites were indicated.

##### Amido Hydrolysis

The metabolites C16, D16, E12, and F12 were indicated as amido hydrolysis products, combined with other reactions. The *m*/*z* 145.0396 product ion was indicative of the oxoindoline structure. Distinguishing the order of the biotransformations was difficult; howeverer, the metabolites (C17–20, C23, D17–20, E14–16, E20–21, F13–15) were ultimately indicated to represent hydrolysis combined with hydroxylation, hydrolysis combined with hydroxylation and further dehydrogenation (C21–22, D21–25, E13), and hydrolysis combined with hydroxylation and further double bond reductive hydrogenation (E17–19, E22–23).

##### N-Dealkylation

The N-dealkylated metabolites, identical for all 4 OXIZIDs, were characterized by a parent ion of *m*/*z* 266.0294, at the same retention time, 6.85 min. In addition, we also observed the combination reaction of N-dealkylation and hydroxylation (D13–15, F17–19).

##### Glucuronidation

Many phase II metabolites (C27–34, D26–30, E25–26, and F20–24) were 176 Da larger than the corresponding phase I metabolites, indicating the presence of a glucuronide. In addition, some of them did not show phase I metabolites, showing instead the corresponding glucuronic acid-bound metabolites of phase I (C33*–C33, F20*–F20), which might be the result of their lower concentrations.

### 3.3. Suggested Biomarkers

We quantified the peak area ratios of the top three metabolites of the six SCs after a 180 min incubation in the pHLM incubation system and then compared the data of the peak area ((Tables S1–S6)). This led us to propose the following metabolites, combined with the parent drugs, as SC biomarkers.

For ADB-BUTINACA, the monohydroxylation (N-butyl chain) (A1), monohydroxylation (t-butyl chain) (A2), and the ADB-BUTINACA acid metabolite (A7) were appropriate biomarkers. The dihydroxylation (pentenyl chain) (B6, B7) and monohydroxylation (indazole ring) (B2) were suggested for MDMB-4en-PINACA. We dispensed with the hydroxylated metabolites with the unreserved ester moiety, as the same metabolites were encountered with ADB-4en-BUTINACA [22].

Due to the overlapping metabolite profiles of the OXIZIDs, we were prudent in selecting biomarkers for each OXIZID. For BZO-HEXOXIZID, the monohydroxylation combined with dehydrogenation (hexyl chain) (C6), monohydroxylation (hexyl chain) (C3), and the ketone metabolites (C11) were suggested. The key Phase I metabolites of BZO-POXIZID and BZO-4en-POXIZID were D6 (monohydroxylation combined with hydrogenation at the pentyl chain), D2 (monohydroxylation at the pentyl chain), D3 (monohydroxylation at the benzene ring of benzoyl), D8 (dihydroxylation at the pentyl chain), E10 (dihydroxylation combined with hydrogenation at the pentenyl chain), E3 (monohydroxylation at the pentenyl chain), and E8 (monohydroxylation at benzene ring of the benzoyl combined with monohydroxylation at the pentenyl chain). The key metabolites of 5-fluoro BZO-POXIZID were F7 (defluorination combined with hydroxylation), F11 (pentanoic acid), F2 (monohydroxylation at the benzene ring of benzoyl), and F1 (monohydroxylation at the benzene ring of benzoyl).

The four OXIZID SCs also contained copious overlapping metabolites (D5–6/E1–3, D7–8/E10, D1–2/E5–6/F6–7), an awkward situation that is frequently encountered in analysis. Therefore, when determining biomarkers, researchers often use the parent drug and several special metabolites as markers to ensure the accuracy of identification [30]. Here, we suggested that the SCs could not be distinguished by their metabolites but instead by their parent compounds. To distinguish 5-fluoro BZO-POXIZID from BZO-POXIZID and BZO-4en-POXIZID, the monohydroxylation products (fluoropentyl chain) (F1–2) were suggested as biomarkers.

## 4. Discussion

The results presented here showed that the biotransformation pathways of the six SCs were the same as those of other SCs with similar structures, with the metabolic sites frequently occurring at the alkyl, alkenyl, and fluoropentyl side chains and benzene ring moieties. These findings were also consistent with previously published metabolic products of SCs [31,32,33]. BZO-HEXOXIZID, BZO-POXIZID, and ADB-BUPINACA were straight-chain hydrocarbon analogues with comparable metabolic patterns. The most abundant metabolites generated in vitro, particularly those resulting from oxidative reactions at the N-alkyl chain, concurred with those for the straight-chain SCs JWH-018, JWH-073, and AB-PINAC and included hydroxylation, dehydrogenation, N-dealkylation, ketone formation, and carboxylation [34,35]. In addition, the 5-fluoro BZO-POXIZID with a fluoropentyl group most frequently had the fluorine atom removed at the 5-position, with a subsequent hydroxylation. According to our results, the biotransformation is likeliest to involve oxidation of the terminal fluorine atom to a hydroxyl group, followed by continuous oxidation reactions of the fluoropentyl group of 5-fluoro BZO-POXIZID to obtain valeric acid (F11); this would be consistent with the metabolism rule for a fluoropentyl group reported in the literature [36,37,38,39]. It is worth noting that under the action of hydroxylase, the fluorine atoms might shift to adjacent positions during hydroxylation similar to the NIH shift, which provided a possibility for derivation of the hydroxylation position of F20* [40,41,42].

For MDMB-4en-PINACA and BZO-4en-POXIZID, which have olefinic side chains, the special metabolite was the formation of a dihydrodiol (the oxidation of the double bond of the olefinic side chain) and even a dihydrotriol. This kind of biotransformation commonly occurs in many compounds with similar structures, such as ADB-4en-PINACA and MMB-4en-PINACA, for which the dihydrodiol has been listed as a biomarker to identify ingestion [22,43]. Dihydrodiol formation from metabolism of alkenes via epoxidation was a common biotransformation [44,45]. Epoxidation was catalyzed by cytochrome P450 enzymes, followed by spontaneous hydration of the epoxide or facilitated by epoxide hydrolase [24]. In addition, previous studies had shown that terminal alkene groups could cause cytochrome P450 enzymes inactivation by heme alkylations and have hepatotoxicity. Therefore, carrying out a deep investigation of the relationship between these compounds and cytochrome P450 enzymes was of great significance for understanding drug–drug interaction and toxicity [46,47,48,49]. All six SCs underwent amide hydrolysis, with MDMB-4en-PINACA also including amide hydrolysis combined with monohydroxylation products (B21–23), which were not detected in previous studies. Therefore, we speculated that an amide hydrolysis metabolite should be present at a very low abundance, although information-dependent acquisition (IDA) did not lead to the generation of spectra.

Compared with the literature, we identified few metabolites of ADB-BUPINACA; however, the results indicated several special metabolites, such as an N-butanoic acid metabolite (A6), a hydrolysis product (A7), and a hydroxylated metabolite (A1) [10]. We also provide the first report of the N-alkyl carboxylate metabolite A6 in liver microsomes. We did not detect hydroxylation occurring at the benzene ring of the parent core, which was consistent with the small amount detected and reported in the literature [20]. We did not detect the dihydrodiol products occurring on the benzene ring; however, the research results of Kronstrand et al. and Kavanagh et al. both detected many dihydrodiol metabolites arising from the benzene ring of the parent core after liver cell incubation and in biological samples [22,50]. This indicated that liver microsomes might lack the enzyme of this biotransformation pathway. Similarly, N-C oxidative dehydrogenation metabolites were reported after liver cell incubation, whereas they were not detected by Sia et al. or in the present study. Kavanagh et al. did not detect the metabolites of partial monohydroxylation of the tert-leucine amide [21,22]. The metabolites of MDMB-4en-PINACA, such as the dihydrodiol and the hydroxylated and dehydrogenated metabolites, were mentioned in previous literature [23,24,27]. Unlike previous studies, we identified some ketone metabolites occurring at the pentenyl (B18–19) or the tert-butyl (B15) moieties, as well as special phase II metabolites, including those produced by ester hydrolysis to carboxylic acid and further glucuronidation (B28). These results suggested that we should combine multiple metabolic models to improve the metabolic spectrum of SC compounds. The human liver microsomes have a sufficient variety of metabolic enzymes (especially cytochrome P450 enzymes); meanwhile, hepatocytes are also widely used in drug metabolism because, since they contain more complete enzymes and cofactors, such as aldehyde oxidase (AO), xanthine oxidase (XO) and sulfotransferases, a wider variety of metabolites can be identified [51,52,53]. This study is the first to report the hydrolysis of the MDMB-4en-PINACA ester to butyrate in liver microsomes.

Overall, 12–16 metabolites were previously identified for three OXIZIDs (BZO-HEXOXIZID, BZO-POXIZID, and 5F-BZO-POXIZID) by Lee et al. [25]. The major metabolic pathways were consistent with our findings, and the detected compounds included hydroxylated, ketonic, carboxylated, amide-hydroxylated, and N-dealkylated metabolites. Our study indicated that hydroxylation occurred more frequently at the N-alkyl moiety than at the benzene ring, while the F7 (terminal fluorine atom was oxidized to a hydroxyl group) of 5F-BZO-POXIZID was the most frequently modified group, consistent with some other research results for 5F-AB-PINACA, 5F-MN-18, and MAM-2201 [34,39,54]. We considered that C5–7 and D5–6 represented oxidative dehydrogenation combined with hydroxylation (346.1554 and 332.1392 resulting from hydroxyl dehydration), but H11, H13, P8, and P10 were identified as ketone formations by Lee et al. [25], although they only provided one fragment ion. In addition, we did not detect H8 (hydroxylation + ketone) reported by Lee et al. [25], although we did identify the metabolites (C24–26) of dihydroxylation combined with dehydrogenation after hydrolysis of BZO-HEXOXIZID. In view of these discrepancies, we recommend further research to confirm the structures of these SCs.

Unlike the research conducted by Lee et al. [25], for each of the four OXIZIDs, we identified the hydrolysis products and the metabolites that undergo further biotransformation after hydrolysis, which was a common fate of compounds with amide bonds. In our study, many two-phase metabolites and trihydroxylation products were also indicated. In addition, for BZO-POXIZID and 5F-BZO-POXIZID, we identified a metabolite arising from N-dealkylation followed by hydroxylation. For some metabolites, the report by Lee et al. [25] failed to provide detailed ionic fragments; however, we carried out detailed fragment structure assignments, which were of great significance for structure inference. We did not identify the metabolites that occurred in the maternal nucleus, and our results did not identify the metabolites of the OXIZID maternal nucleus. Lee et al. [25] indicated that only one hydroxylated metabolite occurred at the maternal nucleus, but was not present in their urine sample. In future studies, researchers could use other metabolic models to confirm whether this maternal core structure is prone to biotransformation.

Deventer et al. [9] found that BZO-HEXOXIZID, BZO-POXIZID, BZO-4en-POXIZID, and 5-fluoro BZO-POXIZID had different binding affinities for CB1 and CB2. BZO-HEXOXIZID, with an N-hexyl group, had the lowest potency and efficacy, followed by BZO-4en-POXIZID with an N-pentenyl group. The pentyl and fluoropentyl analogs BZO-POXIZID and 5F-BZO-POXIZID exhibited higher but quite similar potencies. BZO-POXIZID and 5F-BZO-POXIZID, with pentyl and fluoropentyl groups, respectively, exhibiting higher but quite similar potencies. All compounds examined in this study underwent C-N bond cleavage (N-dealkylation, etc.), whereas, unlike the other two OXIZIDs, BZO-POXIZID and 5-fluoro BZO-POXIZID underwent N-dealkylation combined with hydroxylation. In this study, in contrast to the other two SCRAs, the common characteristic metabolite of BZO-POXIZID and 5F-BZO-POXIZID was the product of N-dealkylation combined with hydroxylation occurring at the benzene ring of the parent nucleus, whereas the structures of these four compounds were the same after removing the side chain. We speculated that the difference resulted from the difficulty of C-N bond cleavage, which might be an effect of the enzyme responsible for this reaction.

Other common products of phase II metabolism arose from UDP glucuronic acid combined with hydroxyl or carboxyl groups. After 3 h of incubation with human liver microsomes, the peak areas of A9, D28, and F21–22 (glucose-bound metabolites) were relatively large. We recommend treatment of urine samples with β-glucuronidase to facilitate the detection of Phase I metabolites and reduce false negative results in actual cases [55].

## 5. Conclusions

Using the same experimental conditions, our study identified and compared the metabolic characteristics of two scaffold types of synthetic cannabinoids: indazole-3-carboxamides and isatin acyl hydrazones. The metabolic spectra of ADB-BUPINACA, MDMB-4en-PINACA, and three OXIZIDs were enhanced, and the metabolites of BZO-4en-POXIZID were identified for the first time.

## Data Availability

The data presented in this study are available in the main article and the supplementary materials.

## Supplementary Materials

The following supporting information can be downloaded at: https://www.mdpi.com/article/10.3390/metabo13040576/s1, Figure S1: Extracted ion chromatograms of ADB-BUTINACA and its metabolites; Figure S2: Extracted ion chromatograms of MDMB-4en-PINACA and its metabolites; Figure S3: Extracted ion chromatograms of BZO-HEXOXIZID and its metabolites; Figure S4: Extracted ion chromatograms of BZO-POXIZID and its metabolites; Figure S5: Extracted ion chromatograms of BZO-4en-POXIZID and its metabolites; Figure S6: Extracted ion chromatograms of 5-fluoro BZO-POXIZID and its metabolites; Figure S7: Mass spectrometry spectra and fragmentation of ADB-BUTINACA and its metabolites; Figure S8: Mass spectrometry spectra and fragmentation of MDMB-4en-PINACA and its metabolites; Figure S9: Mass spectrometry spectra and fragmentation of BZO-HEXOXIZID and its metabolites; Figure S10: Mass spectrometry spectra and fragmentation of BZO-POXIZID and its metabolites; Figure S11: Mass spectrometry spectra and fragmentation of BZO-4en-POXIZID and its metabolites; Figure S12: Mass spectrometry spectra and fragmentation of 5-fluoro BZO-POXIZID and its metabolites; Table S1: UHPLC-QE-Orbitrap MS data of ADB-BUTINACA and its metabolites; Table S2: UHPLC-QE-Orbitrap MS data of MDMB-4en-PINACA and its metabolites; Table S3: UHPLC-QE-Orbitrap MS data of BZO-HEXOXIZID and its metabolites; Table S4: UHPLC-QE-Orbitrap MS data of BZO-POXIZID and its metabolites; Table S5: UHPLC-QE-Orbitrap MS data of BZO-4en-POXIZID and its metabolites; Table S6: UHPLC-QE-Orbitrap MS data of 5-fluoro BZO-POXIZID and its metabolites.
