# Supplementary material for: Study of the Metabolic Profiles of “Indazole-3-Carboxamide” and “Isatin Acyl Hydrazone” (OXIZID) Synthetic Cannabinoids in a Human Liver Microsome Model Using UHPLC-QE Orbitrap MS
Source: Metabolites. 2023 Apr 18;13(4):576. doi: 10.3390/metabo13040576 (PMC10141538; doi:10.3390/metabo13040576)

Figure S1 Extracted ion chromatograms of ADB-BUTINACA and its metabolites

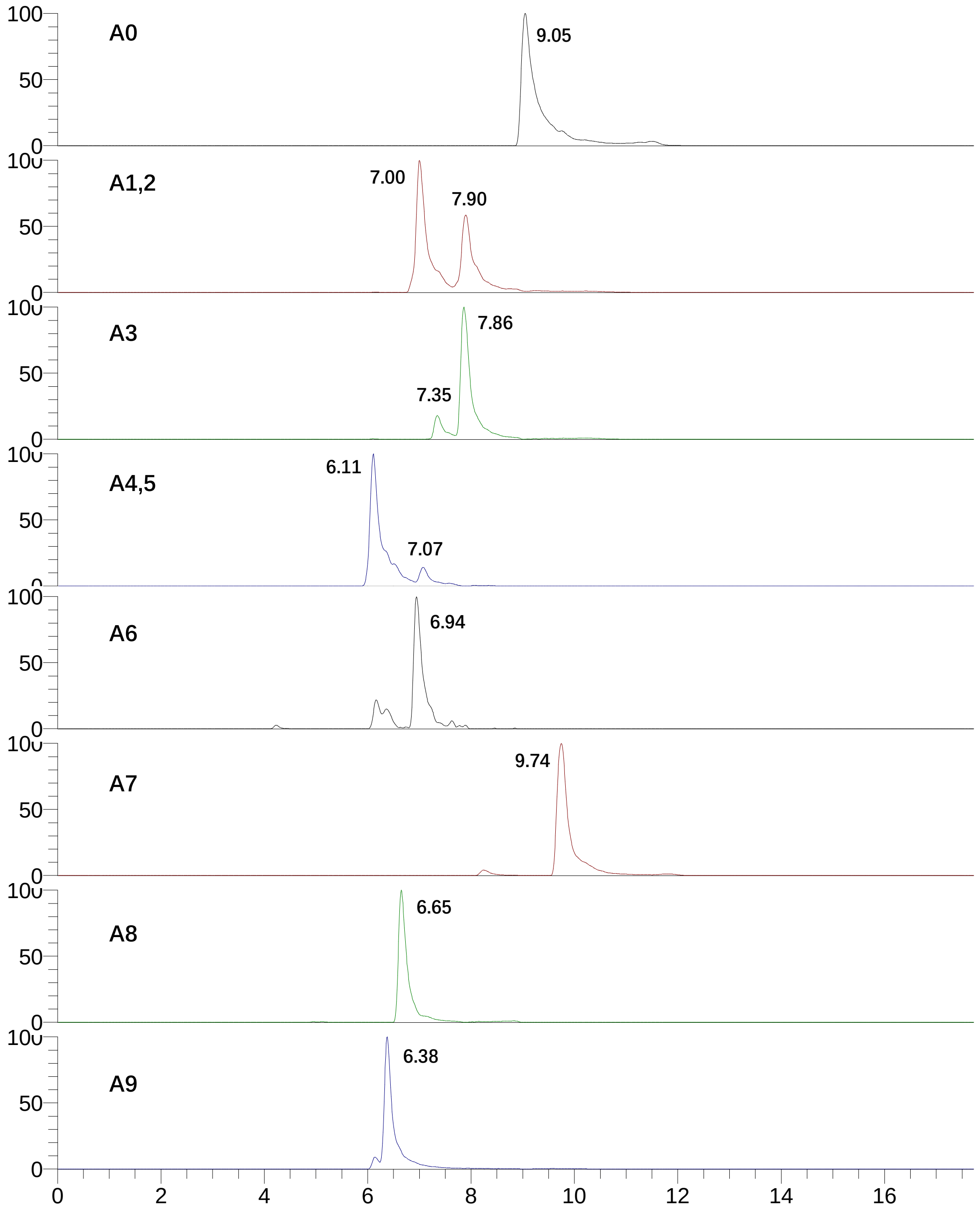

Figure S2 Extracted ion chromatograms of MDMB-4en-PINACA and its metabolites

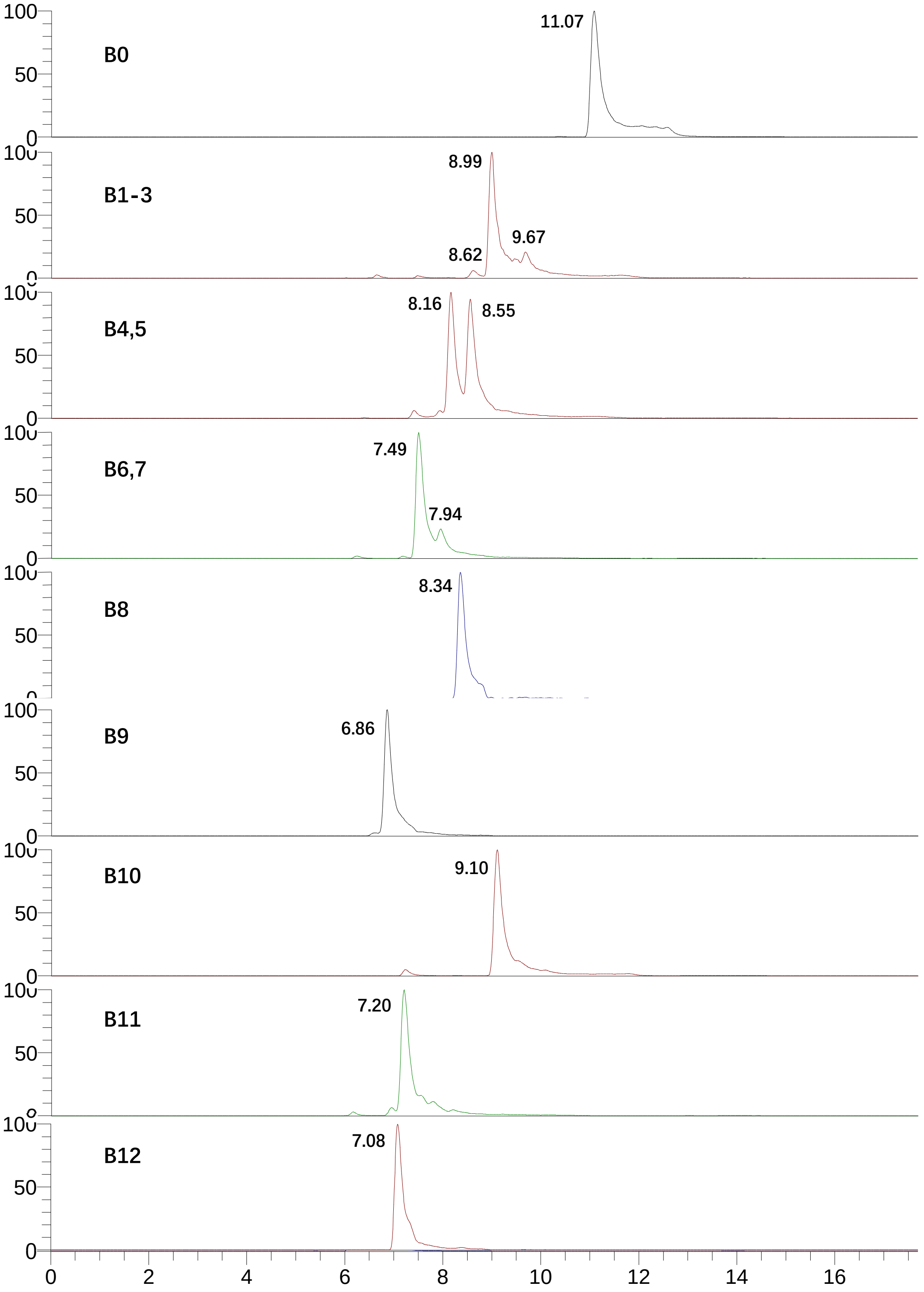

Figure S2 (continued)

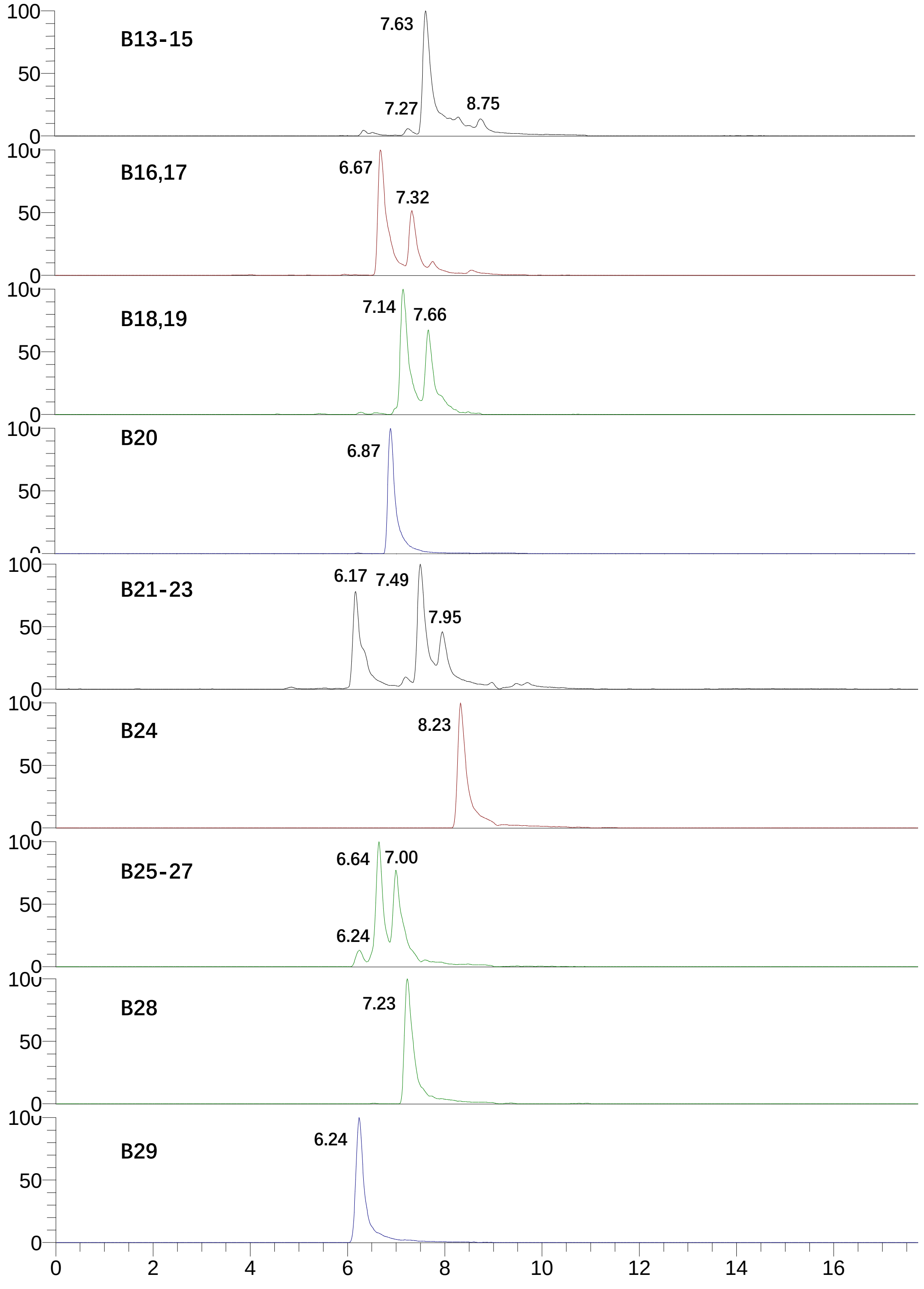

Figure S3 Extracted ion chromatograms of BZO-HEXOXIZID and its metabolites

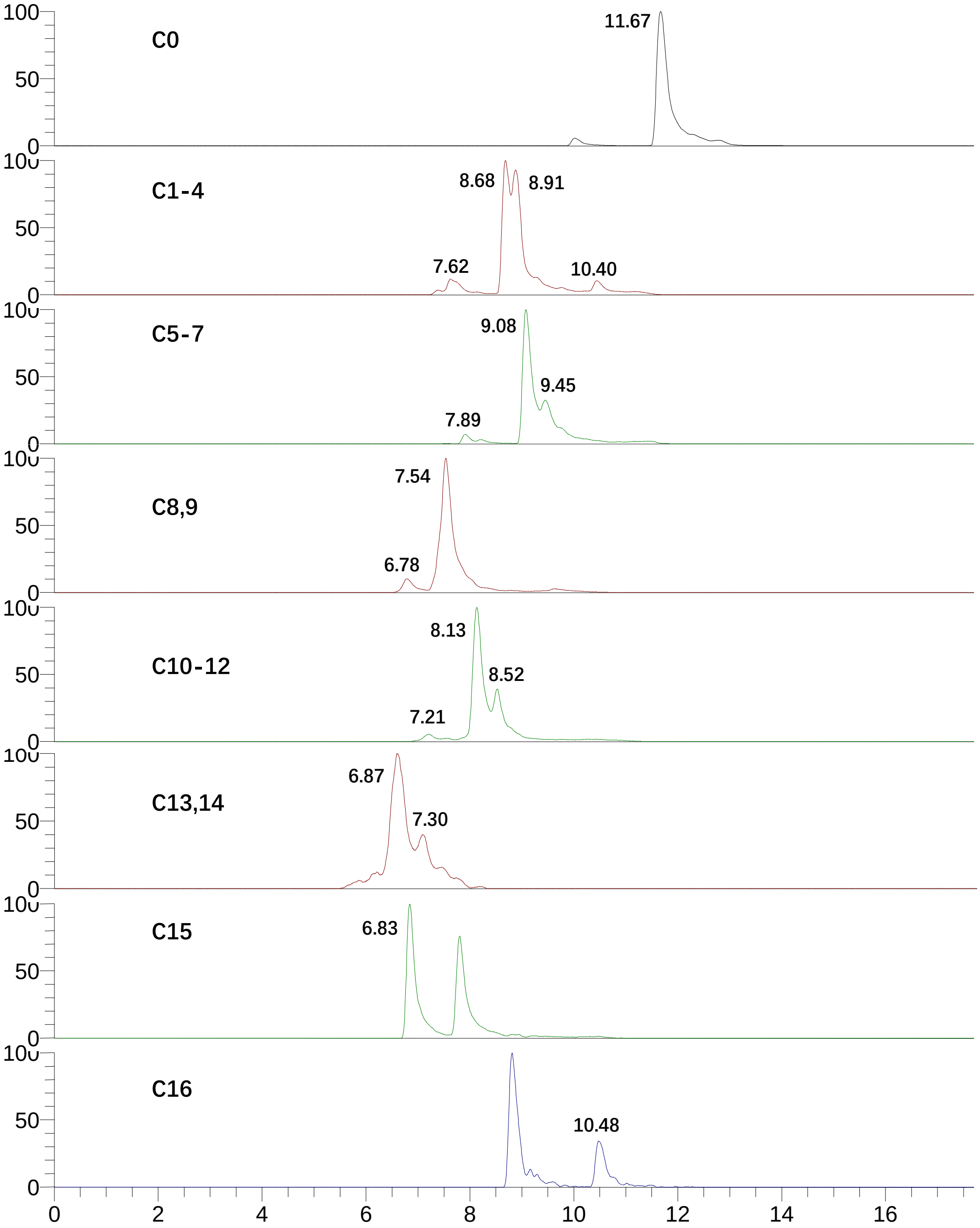

Figure S3 (continued)

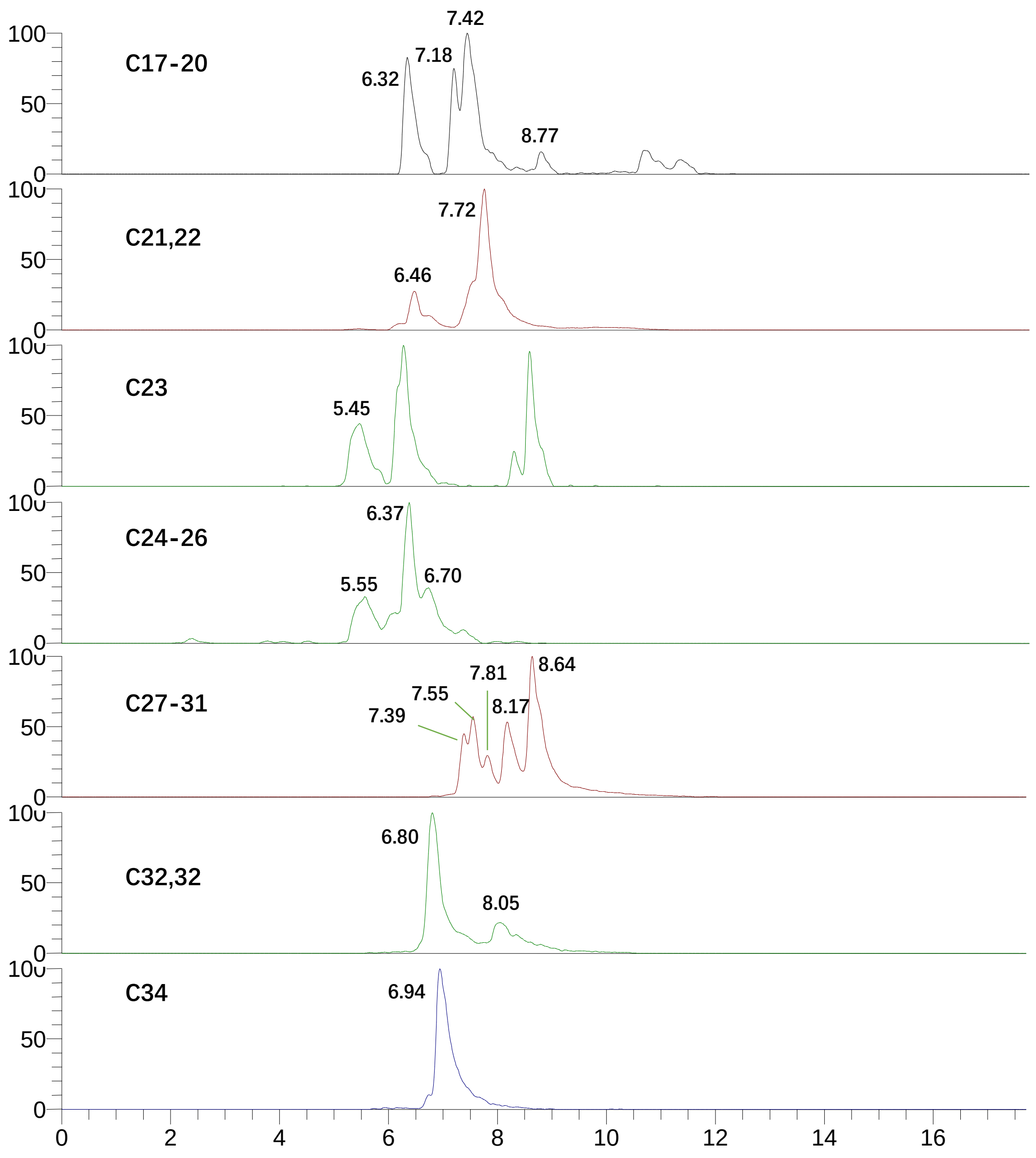

Figure S4 Extracted ion chromatograms of BZO-POXIZID and its metabolites

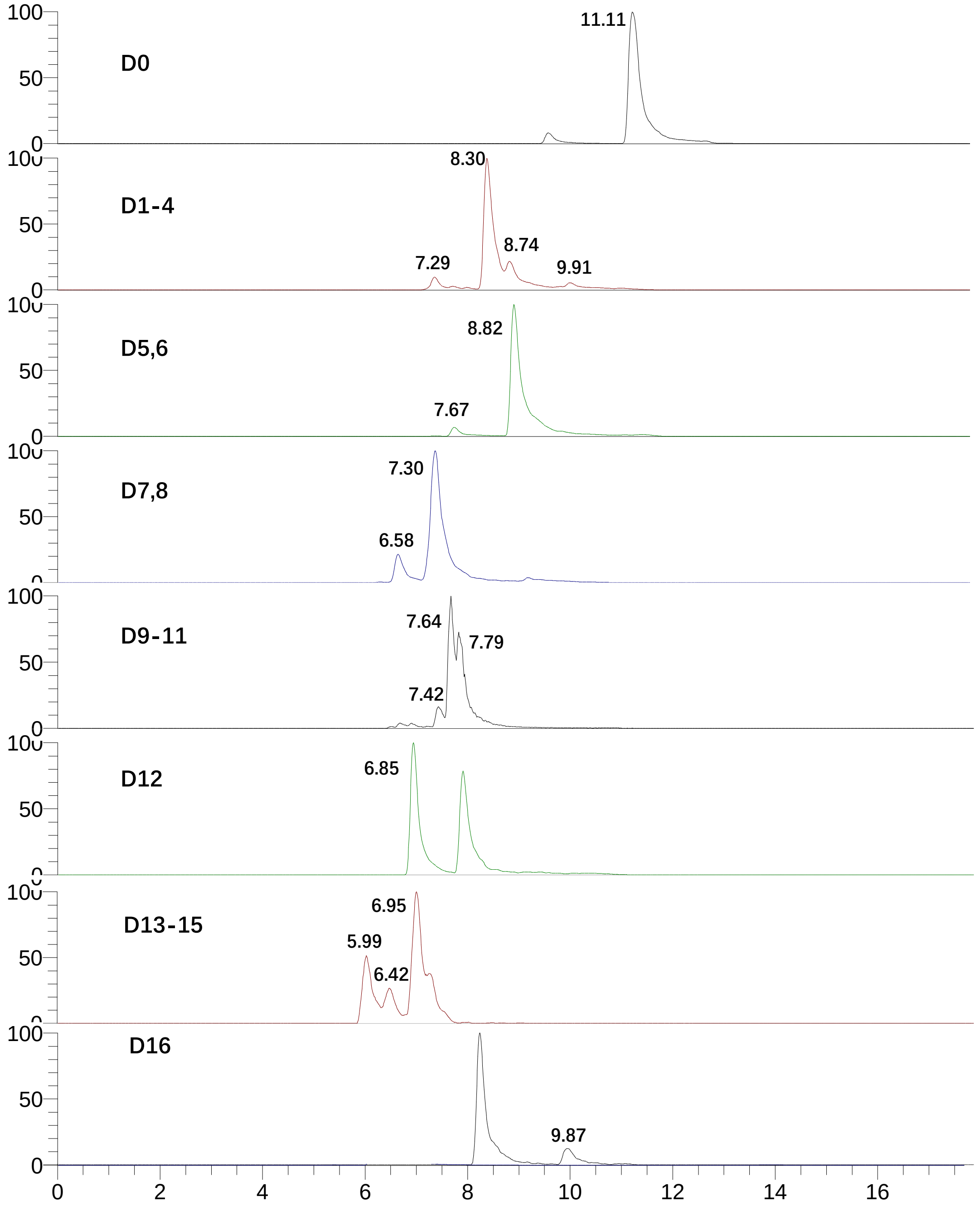

Figure S4 (continued)

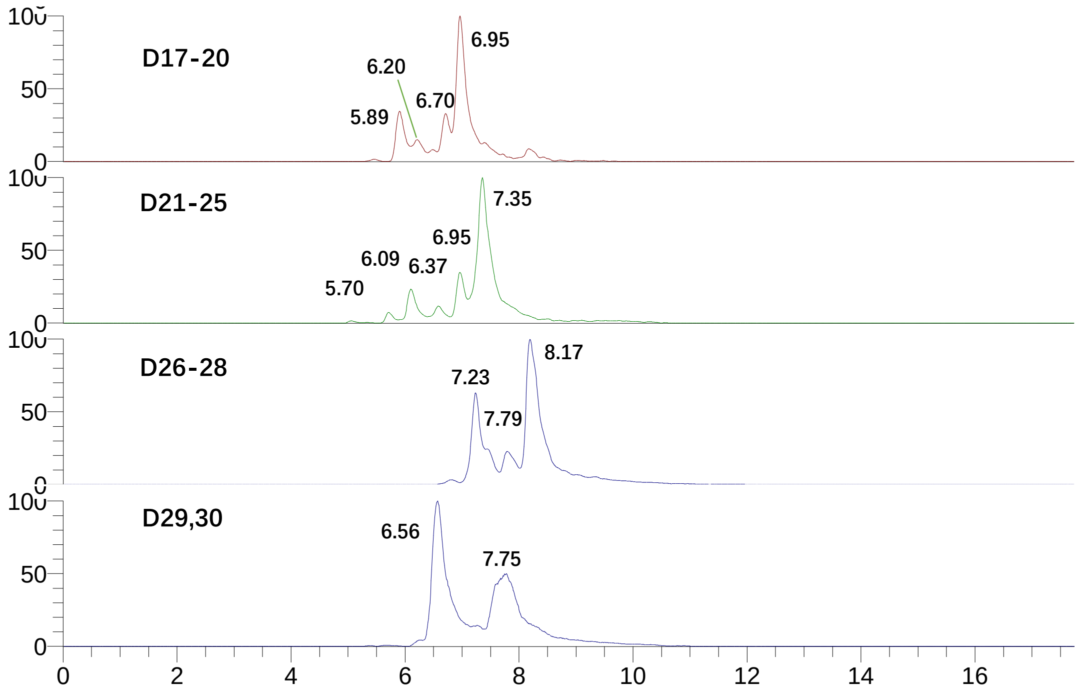

Figure S5 Extracted ion chromatograms of BZO-4en-POXIZID and its metabolites

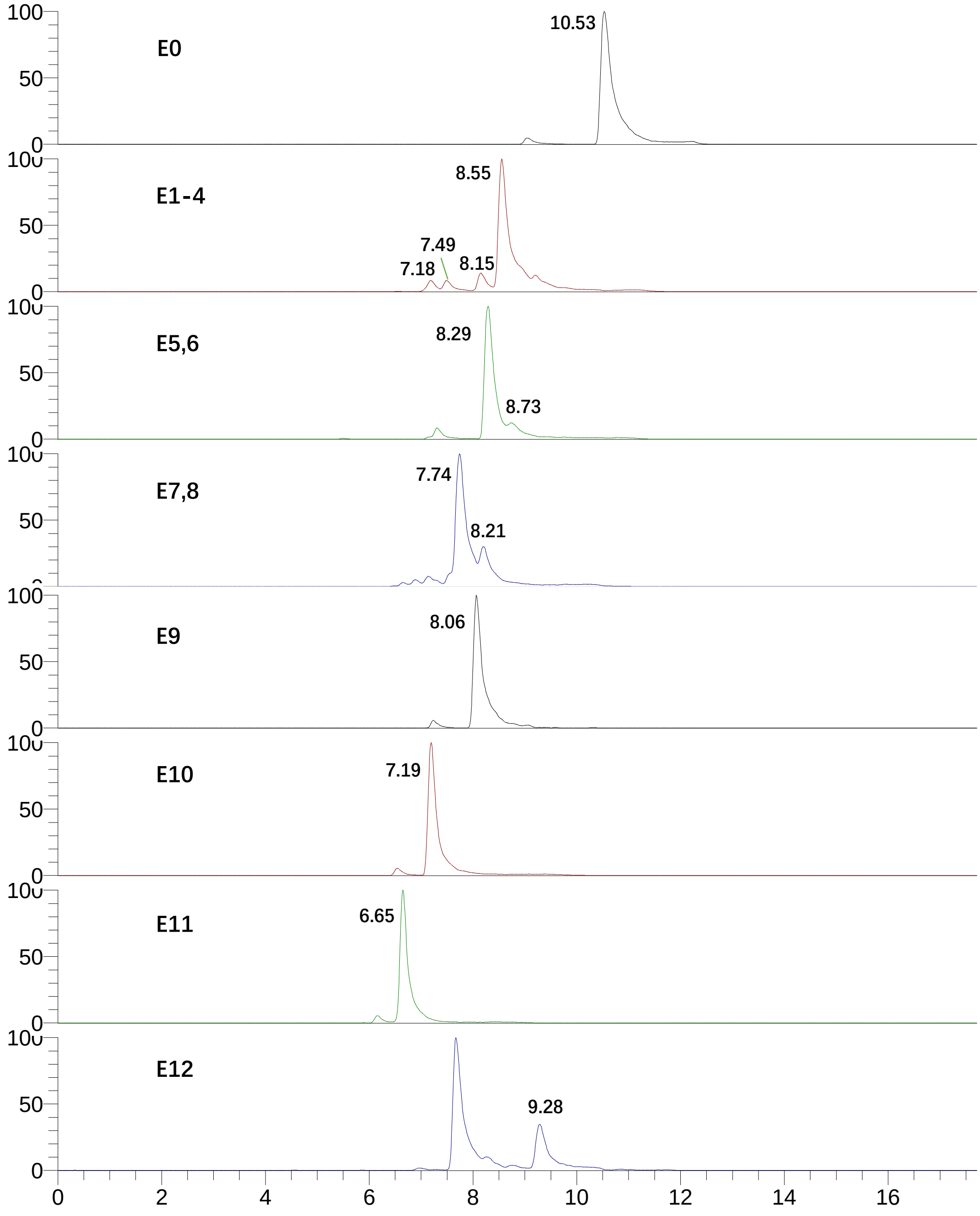

Figure S5 (continued)

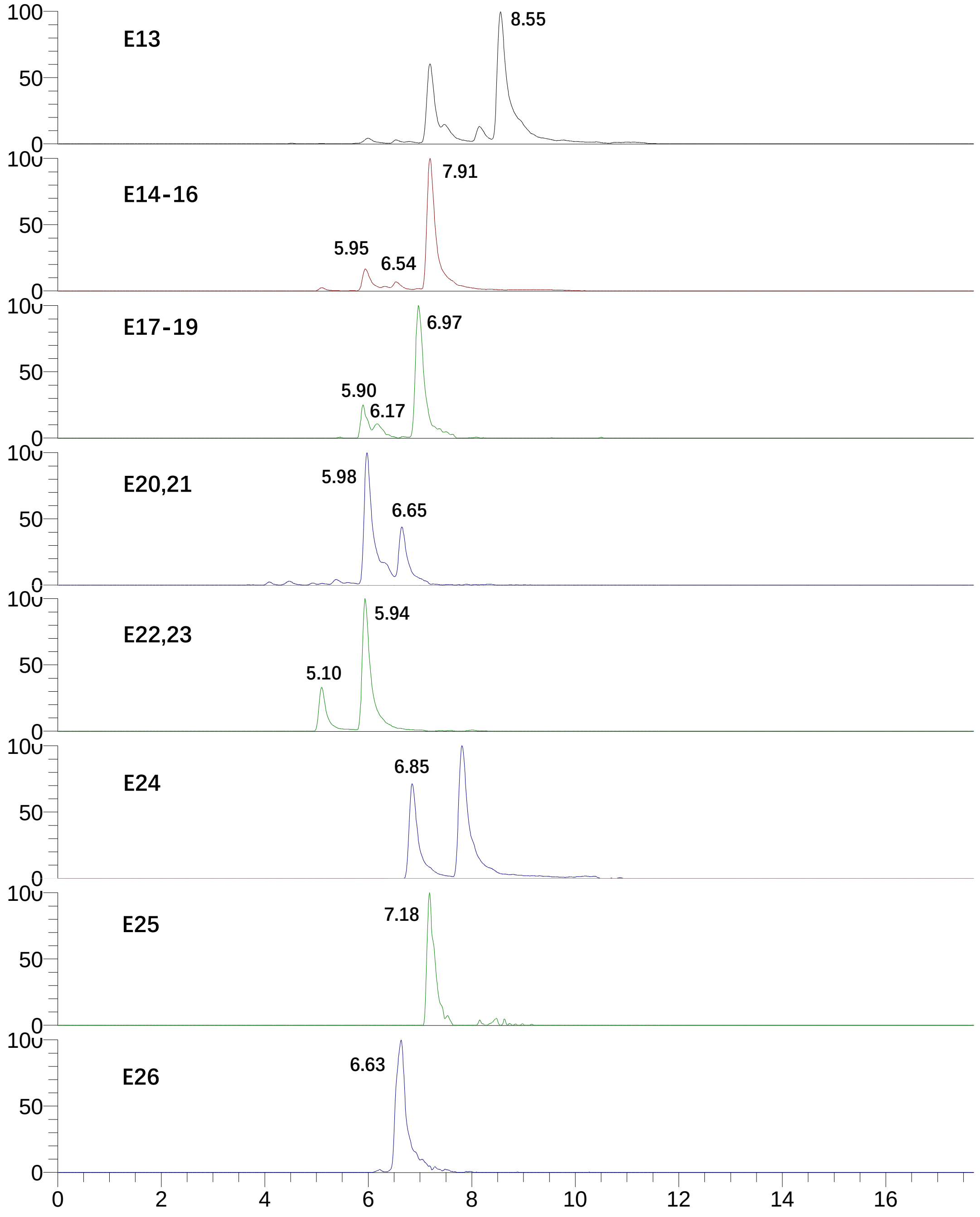

Figure S6 Extracted ion chromatograms of 5-fluoro BZO-POXIZID and its metabolites

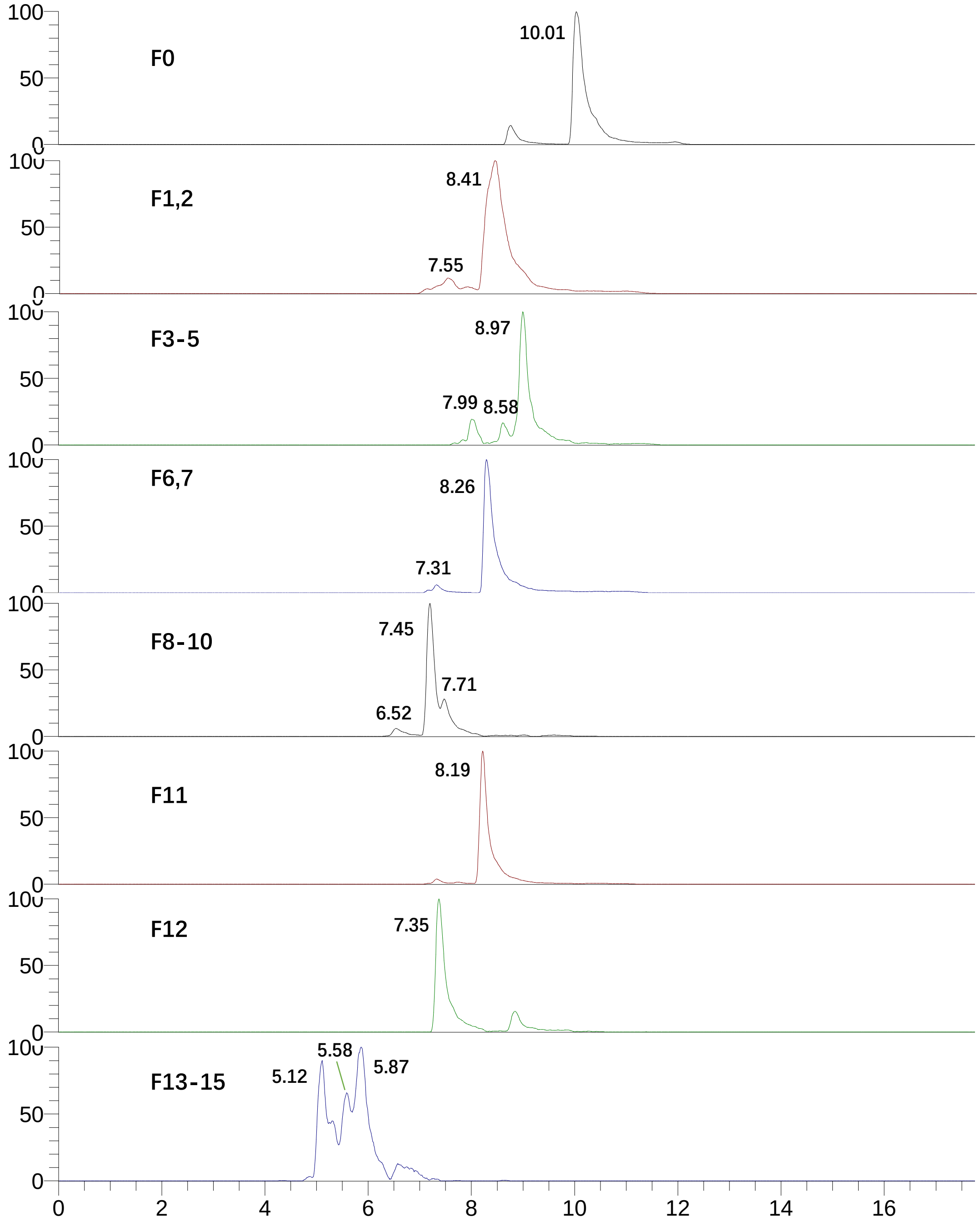

Figure S6 (continued)

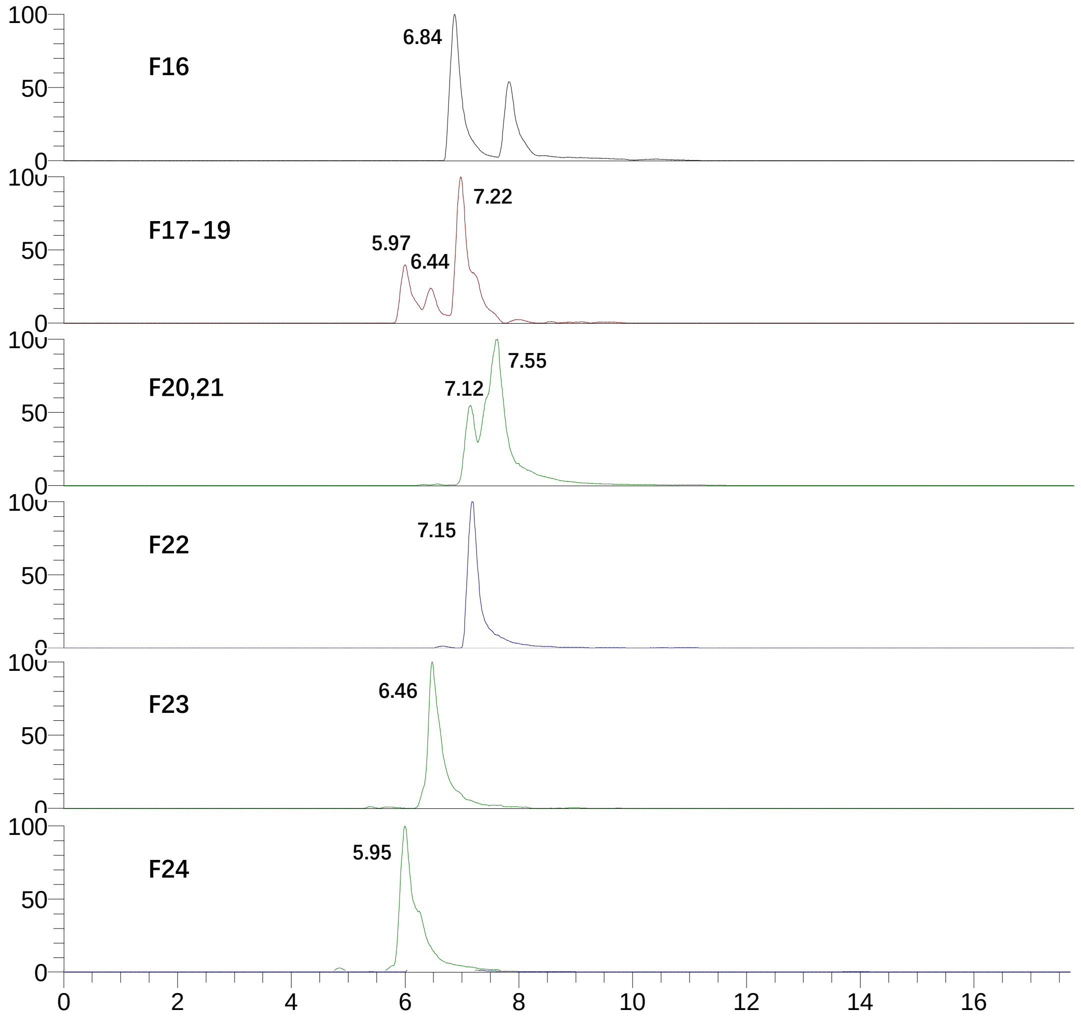

Supplement: Supplementary file 1 [file metabolites-13-00576-s001.zip › Figures S1-S6.pdf]
