# Supplementary material for: Study of the Metabolic Profiles of “Indazole-3-Carboxamide” and “Isatin Acyl Hydrazone” (OXIZID) Synthetic Cannabinoids in a Human Liver Microsome Model Using UHPLC-QE Orbitrap MS
Source: Metabolites. 2023 Apr 18;13(4):576. doi: 10.3390/metabo13040576 (PMC10141538; doi:10.3390/metabo13040576)
Supplement: Supplementary file 1 [file metabolites-13-00576-s001.zip › Figures S7-S12.pdf]

Figure S7. Mass spectrometry spectra and fragmentation of ADB-BUTINACA and its metabolites.

A0

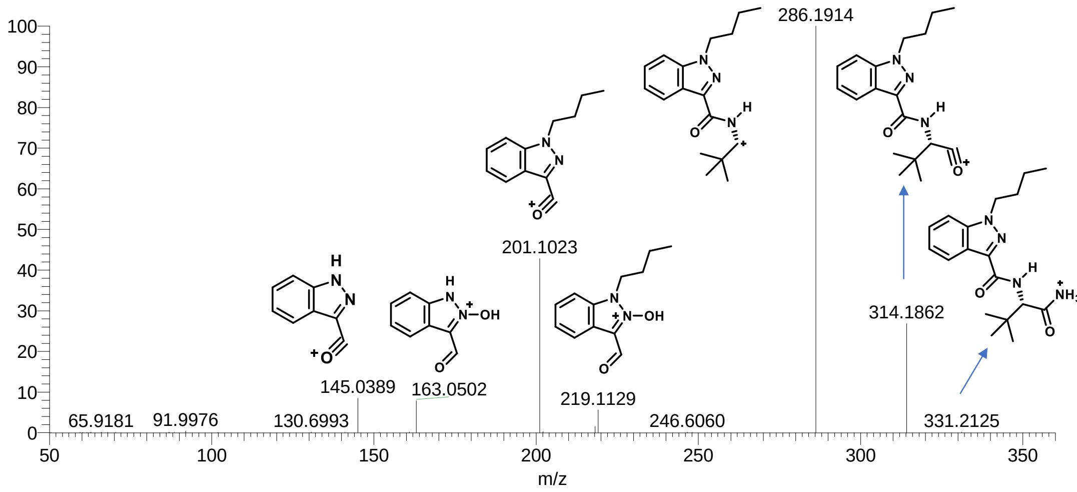

A1

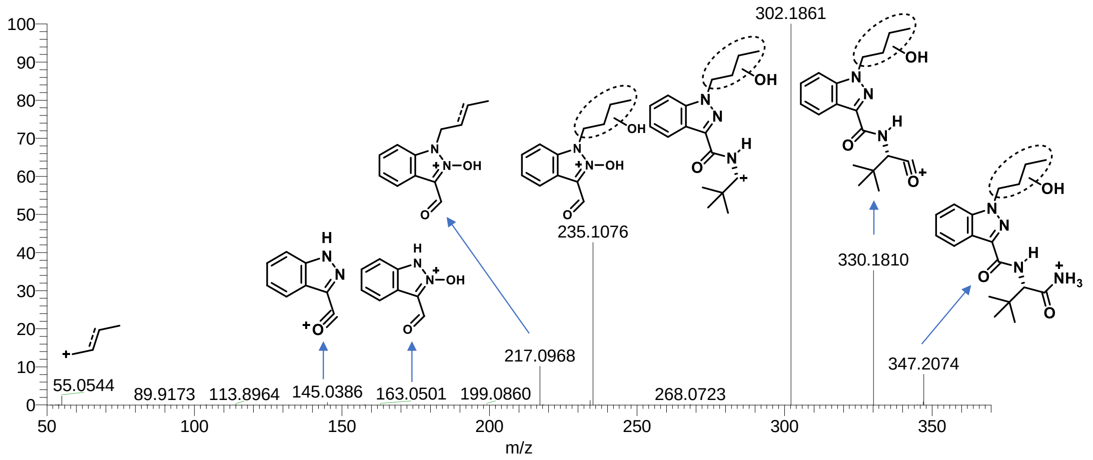

A2

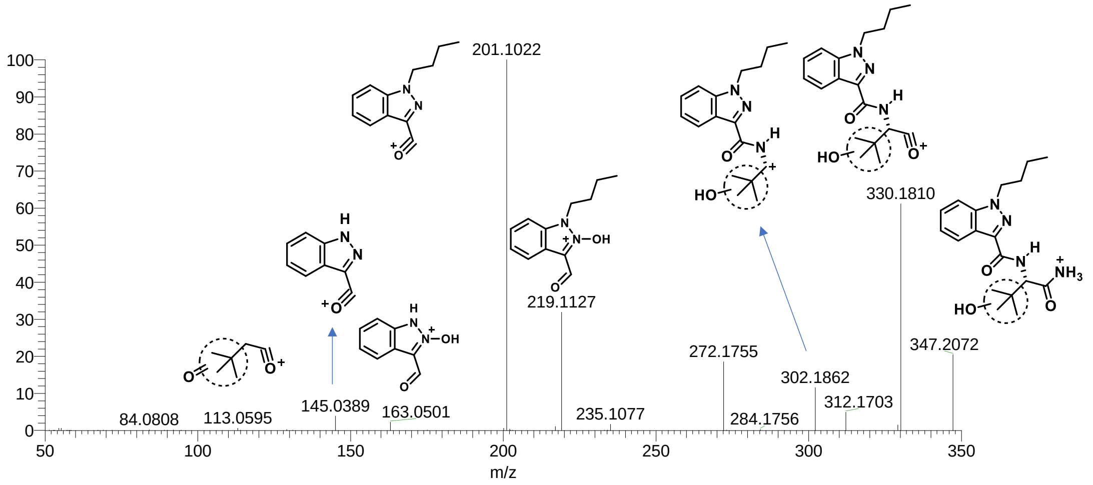

A3

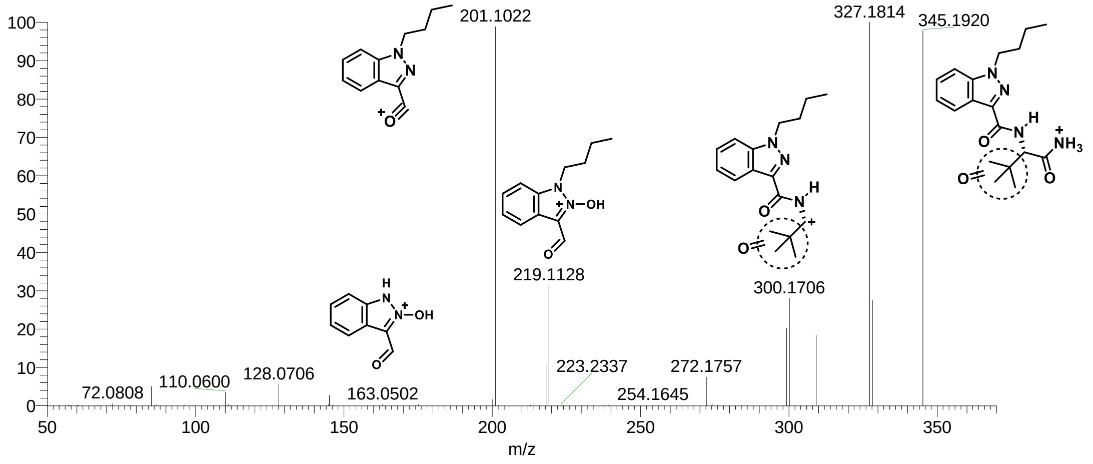

100
90
80
70
60
50
40
30
20
10
0
50
100
150
200
250
300
350
m/z
201.1022
327.1814
345.1920
219.1128
300.1706
223.2337
272.1757
72.0808
110.0600
128.0706
163.0502
254.1645
N
N
O
N-OH
OH
H
NH3

A4,5

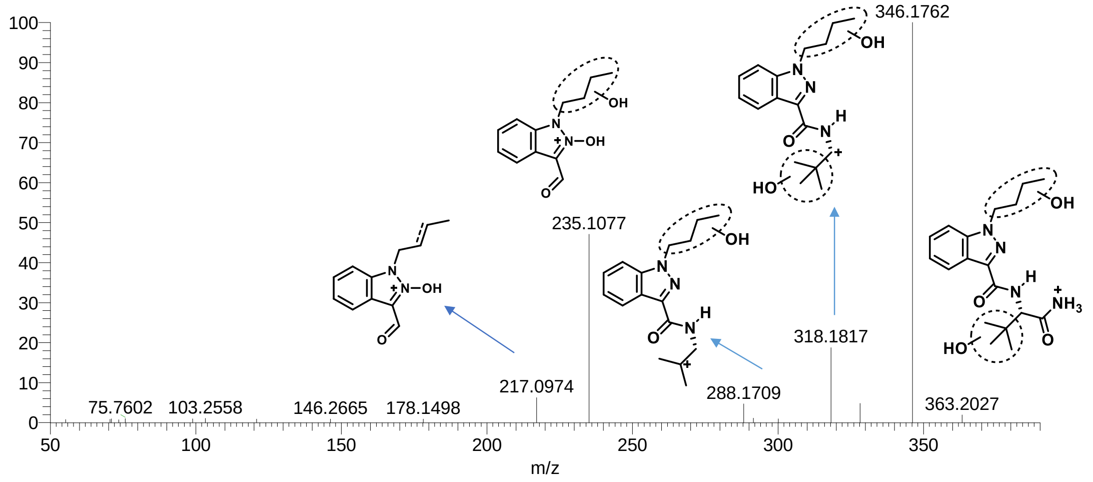

100
90
80
70
60
50
40
30
20
10
0
50
100
150
200
250
300
350
m/z
346.1762
235.1077
318.1817
217.0974
288.1709
363.2027
75.7602
103.2558
146.2665
178.1498
OH
N-OH
HO
NH3

A6

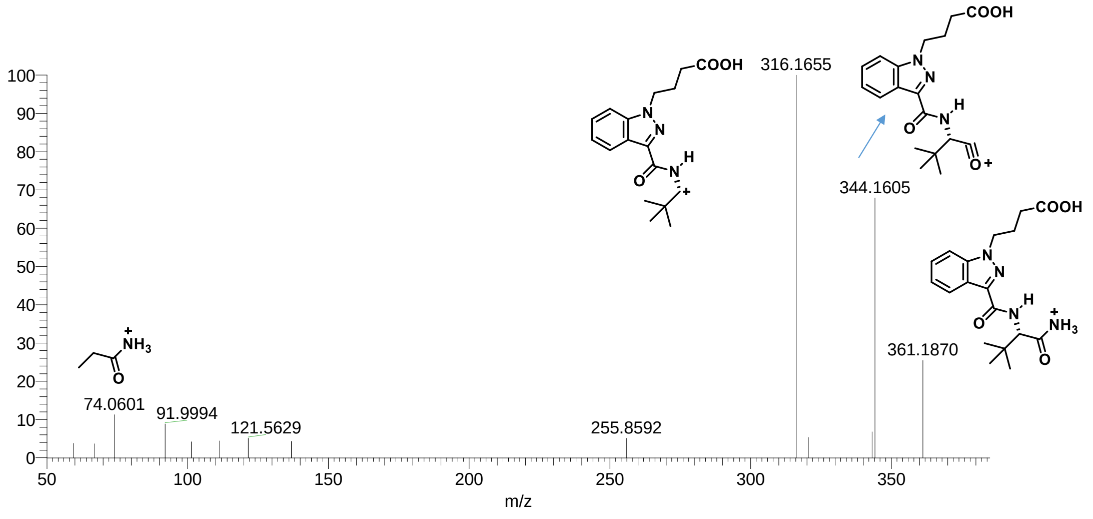

100
90
80
70
60
50
40
30
20
10
0
50
100
150
200
250
300
350
m/z
316.1655
344.1605
361.1870
74.0601
91.9994
121.5629
255.8592
COOH
NH3
O

A7

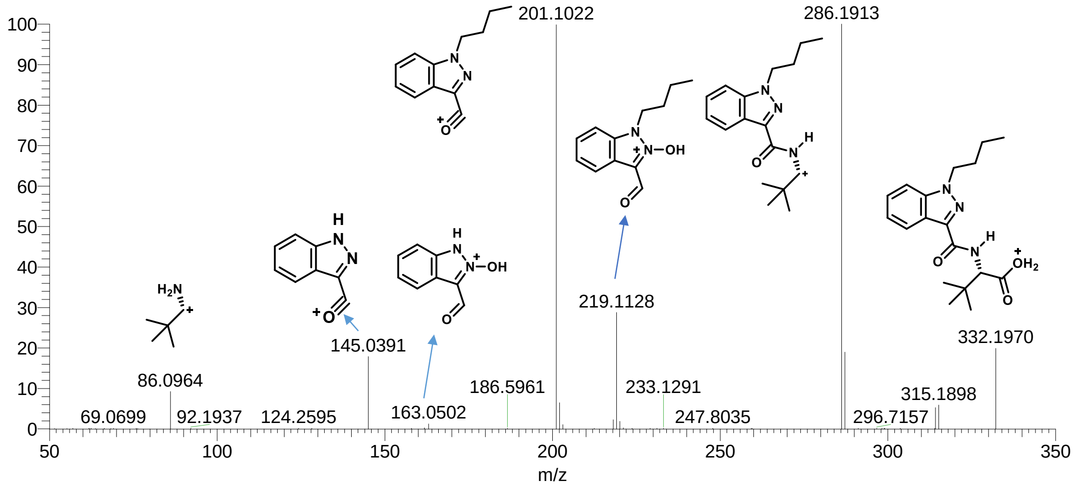
201.1022
286.1913
219.1128
145.0391
332.1970
86.0964
163.0502
186.5961
233.1291
315.1898
69.0699
92.1937
124.2595
247.8035
296.7157
m/z

A8

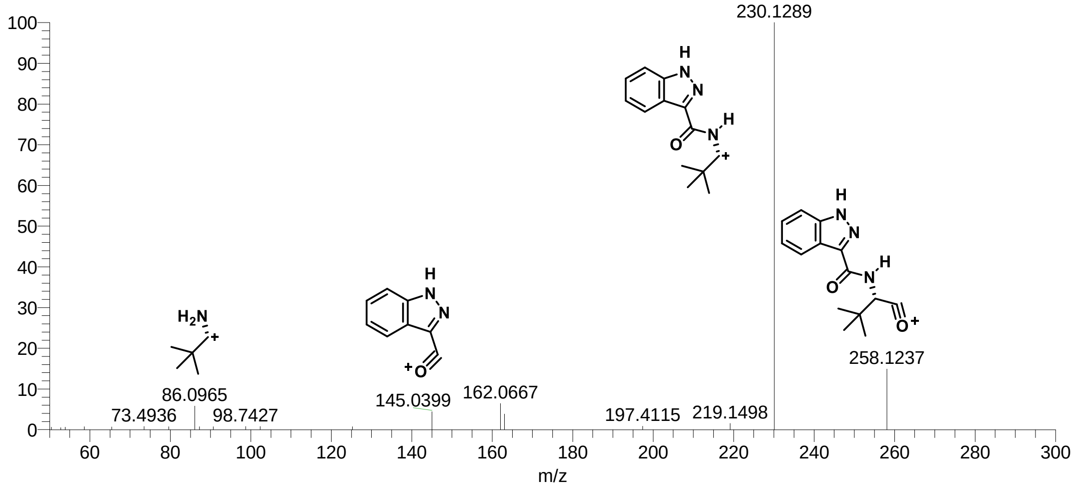
230.1289
258.1237
86.0965
145.0399
162.0667
73.4936
98.7427
197.4115
219.1498
m/z

A9

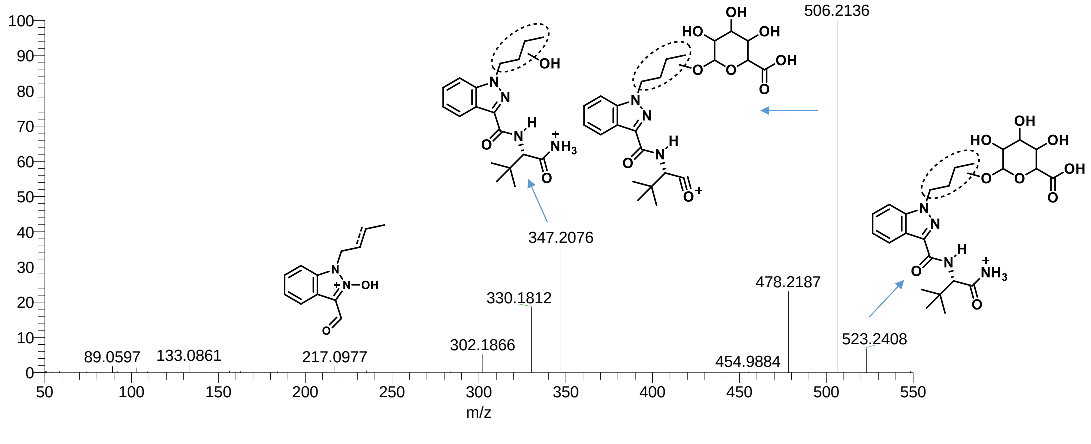
506.2136
347.2076
478.2187
330.1812
523.2408
302.1866
89.0597
133.0861
217.0977
454.9884
m/z

Figure S8. Mass spectrometry spectra and fragmentation of MDMB-4en-PINACA and its metabolites.

B0

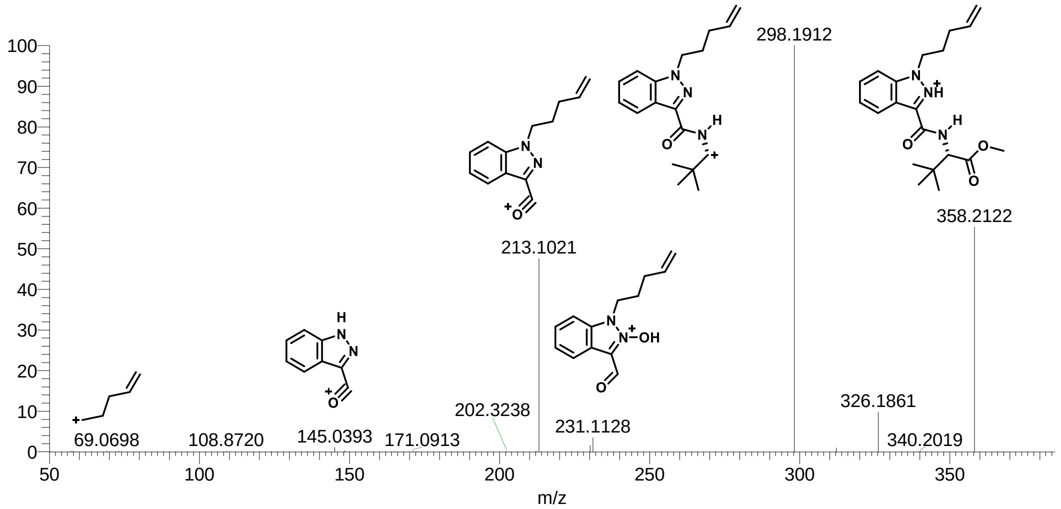

B1

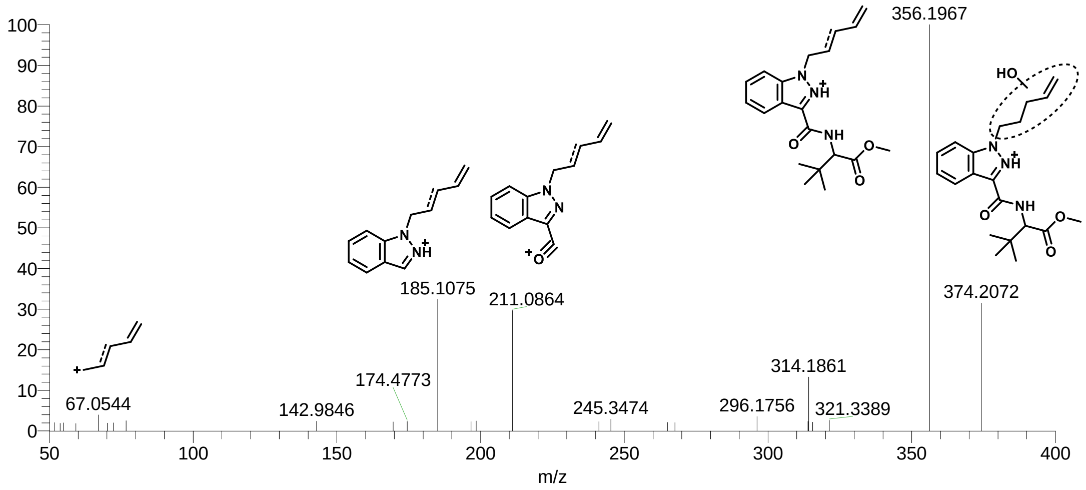

B2,3

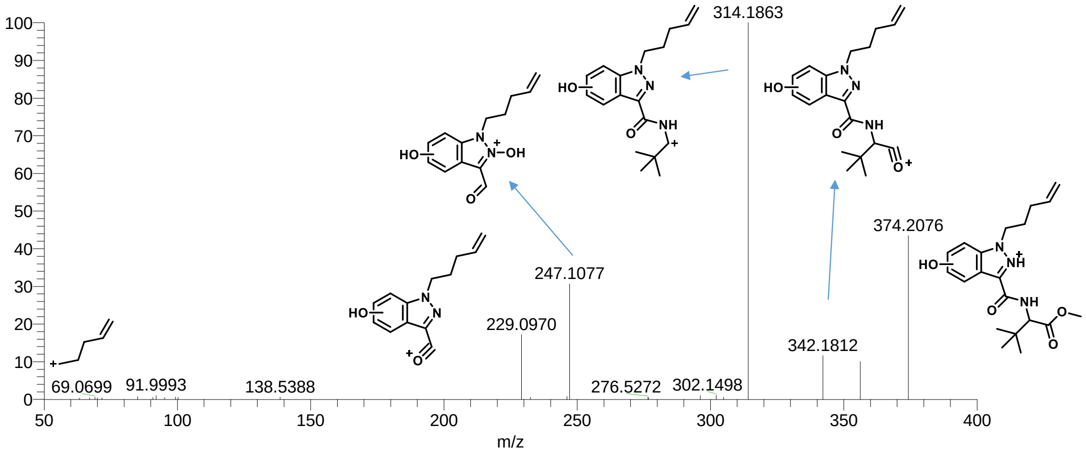

B4,5

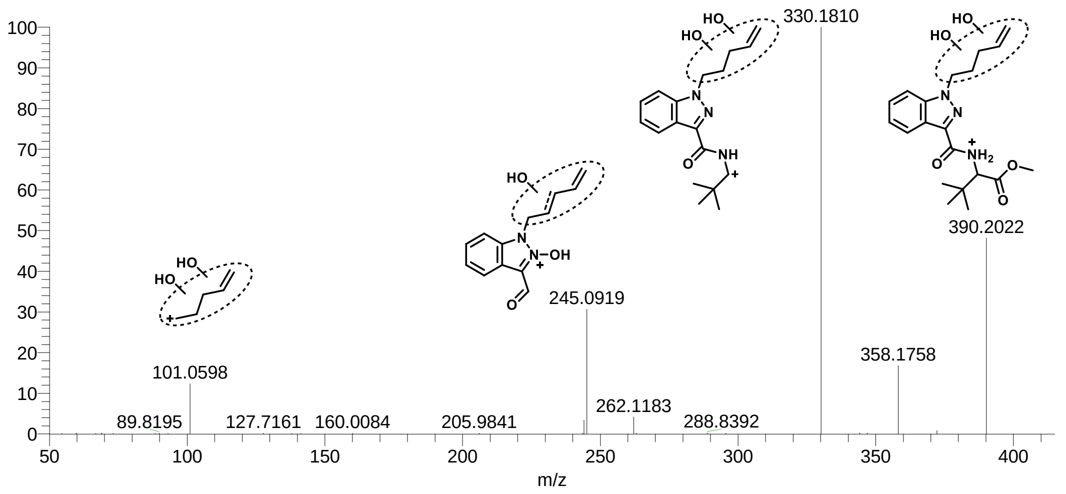
330.1810
390.2022
245.0919
101.0598
358.1758
262.1183
89.8195
127.7161
160.0084
205.9841
288.8392
m/z

B6,7

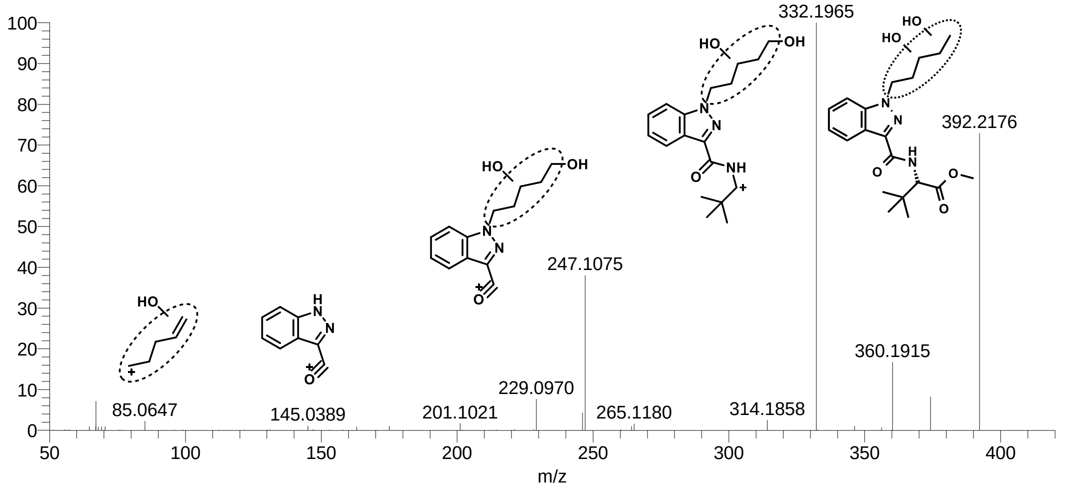
332.1965
392.2176
247.1075
360.1915
229.0970
85.0647
145.0389
201.1021
265.1180
314.1858
m/z

B8

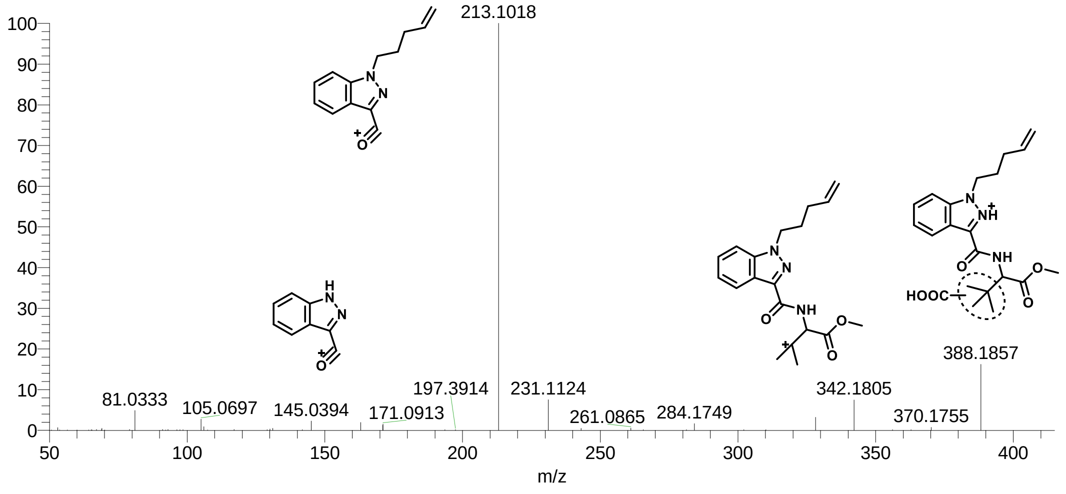
213.1018
388.1857
81.0333
105.0697
145.0394
171.0913
197.3914
231.1124
261.0865
284.1749
342.1805
370.1755
m/z

B9

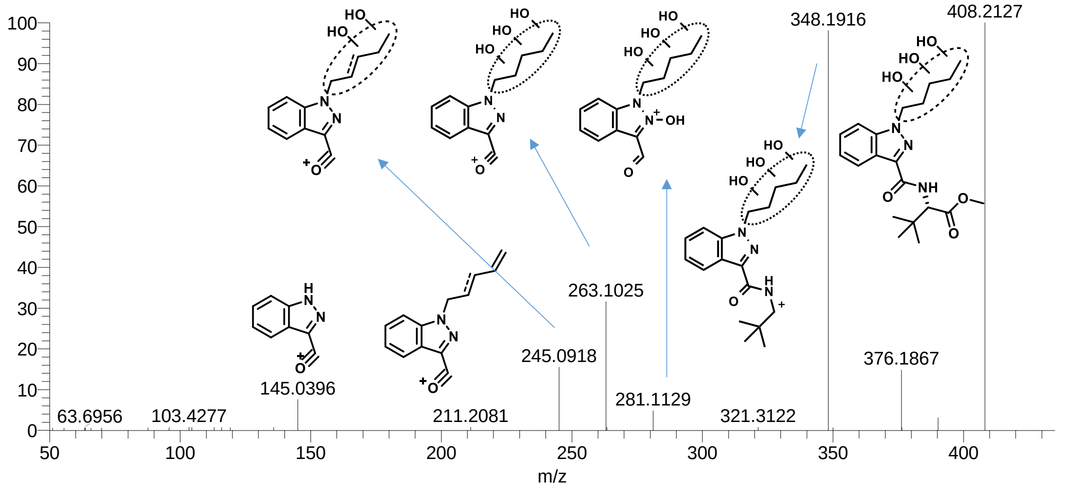

408.2127
348.1916
263.1025
245.0918
145.0396
376.1867
281.1129
63.6956
103.4277
211.2081
321.3122
m/z

B10

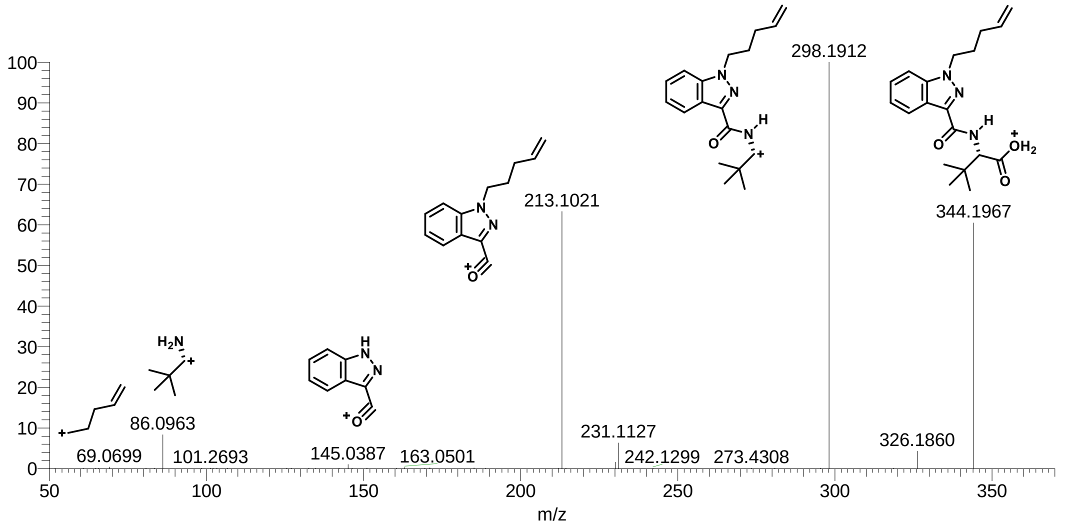

298.1912
213.1021
344.1967
86.0963
231.1127
326.1860
69.0699
101.2693
145.0387
163.0501
242.1299
273.4308
m/z

B11

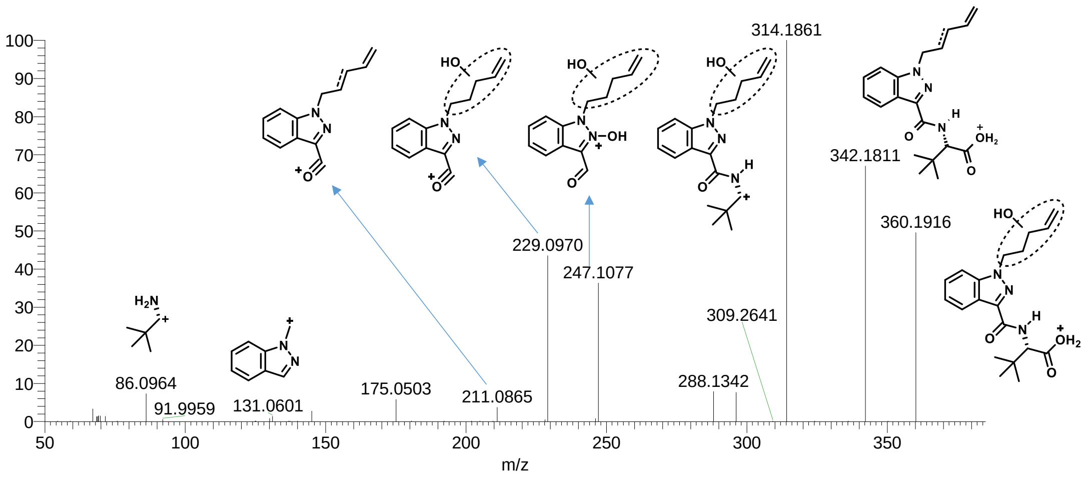

314.1861
342.1811
360.1916
229.0970
247.1077
309.2641
86.0964
91.9959
131.0601
175.0503
211.0865
288.1342
m/z

B12

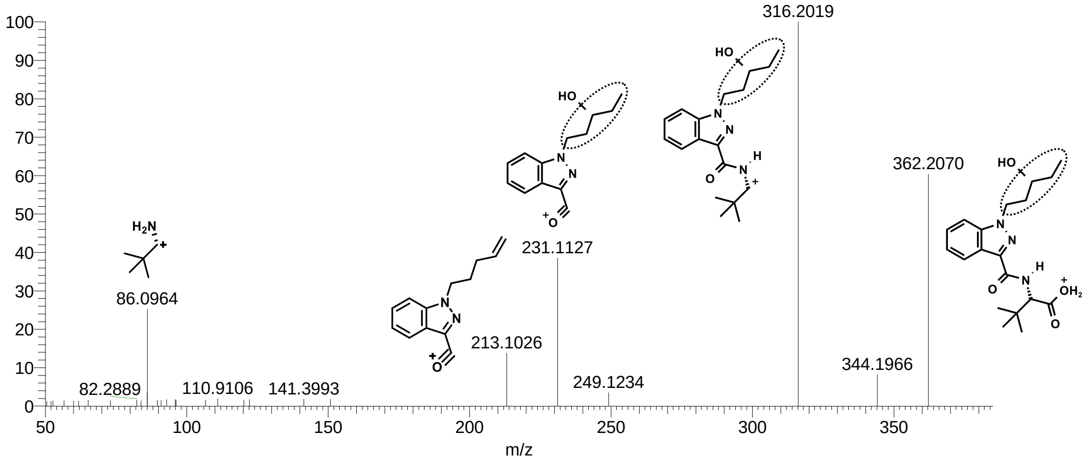

B13,14

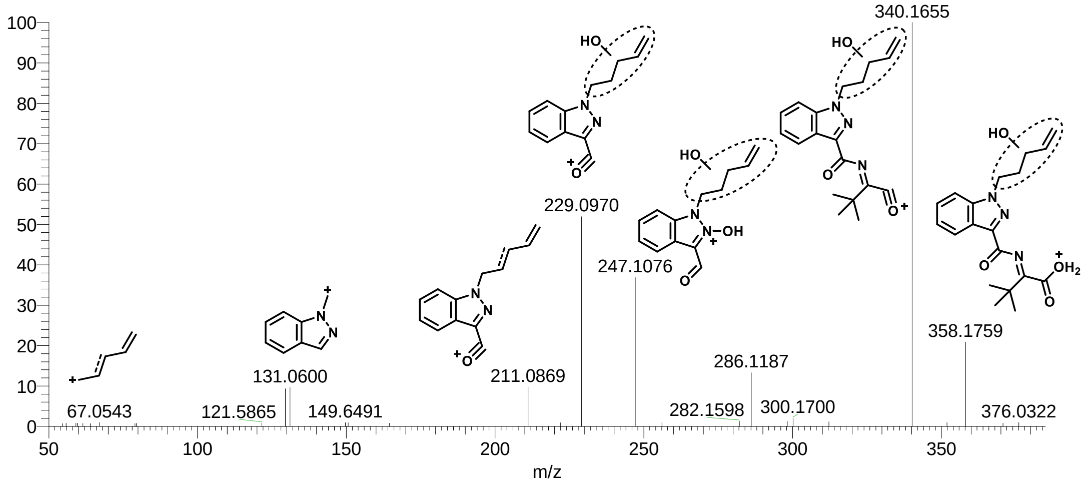

B15

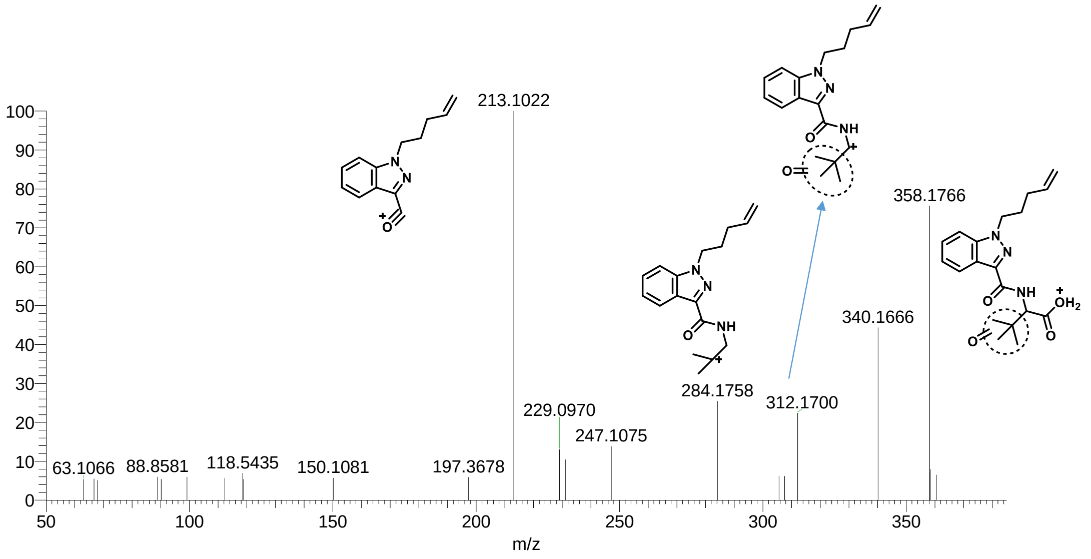

B16,17

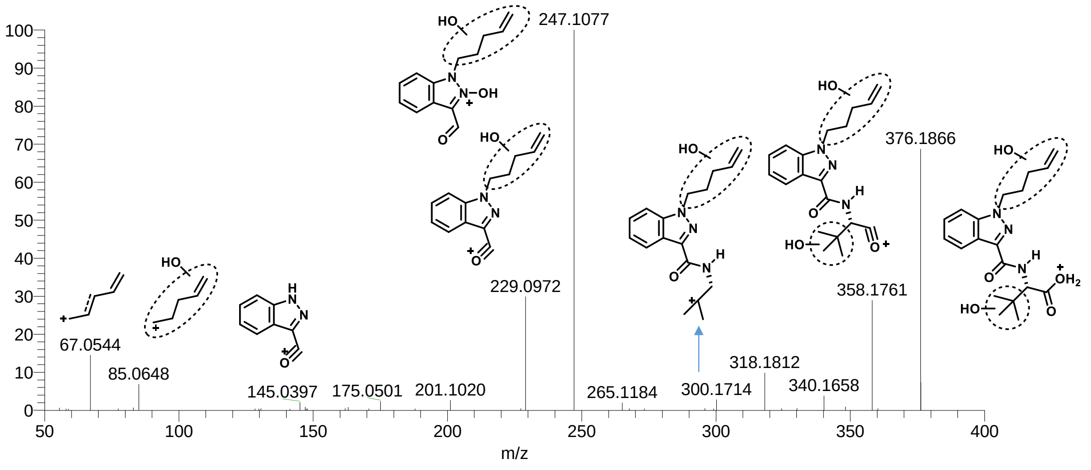

B18,19

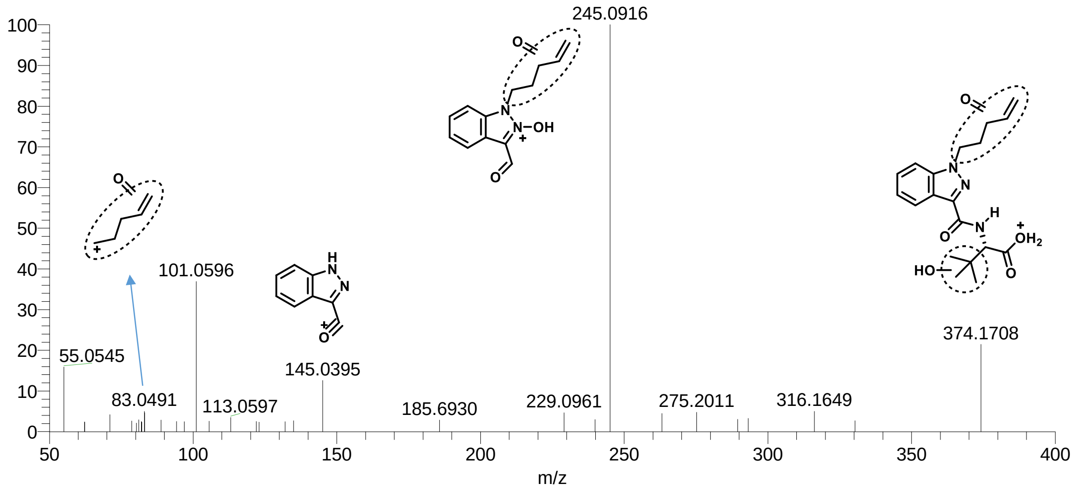

B20

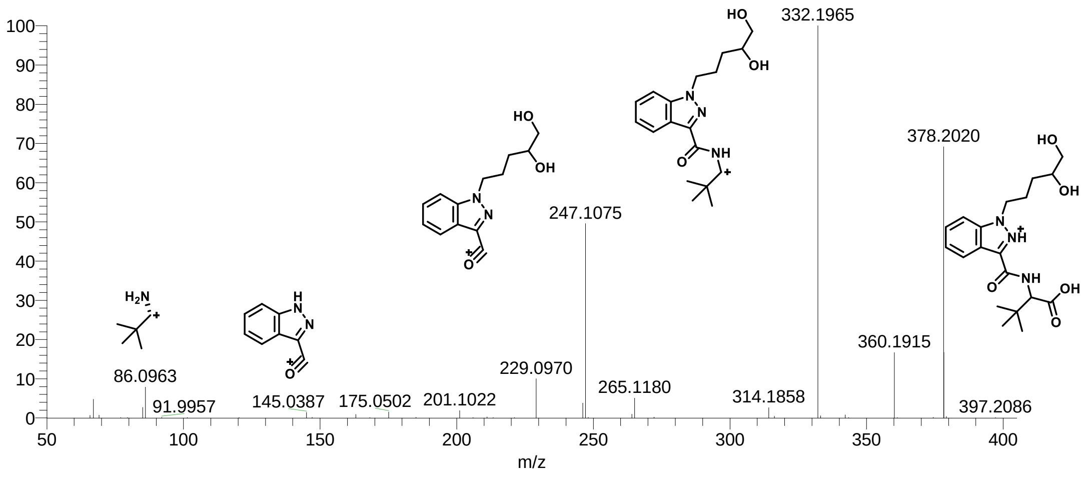

B21-23

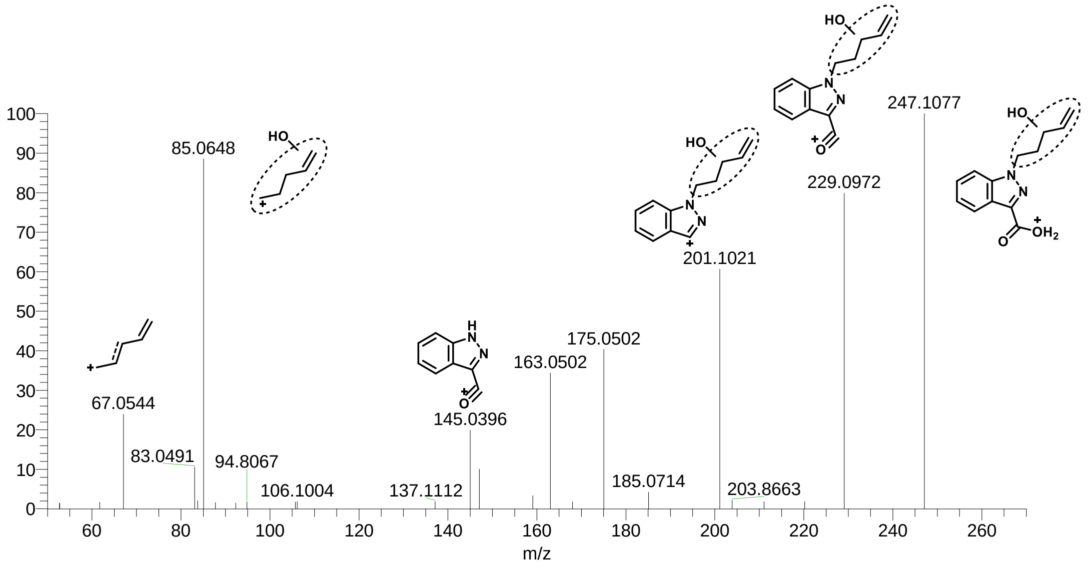

B24

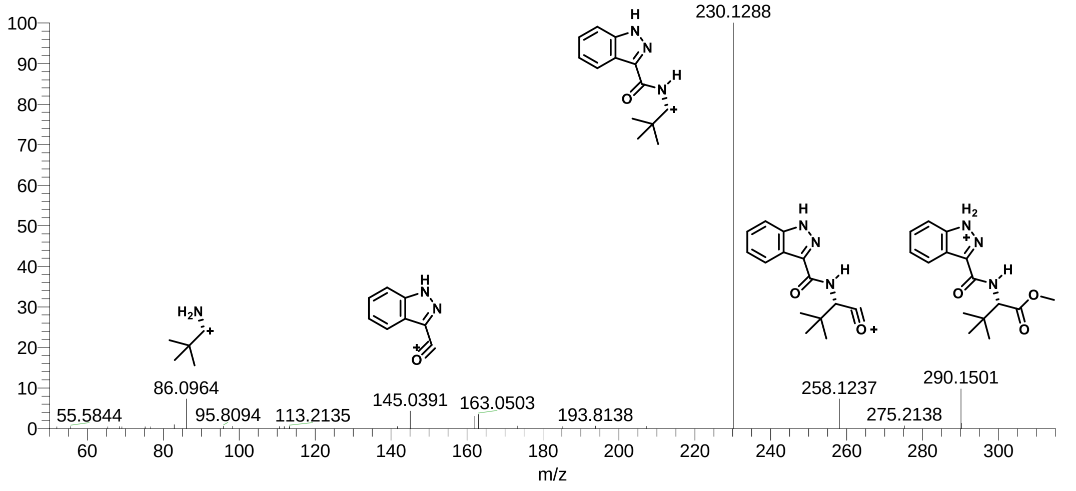

B25-27

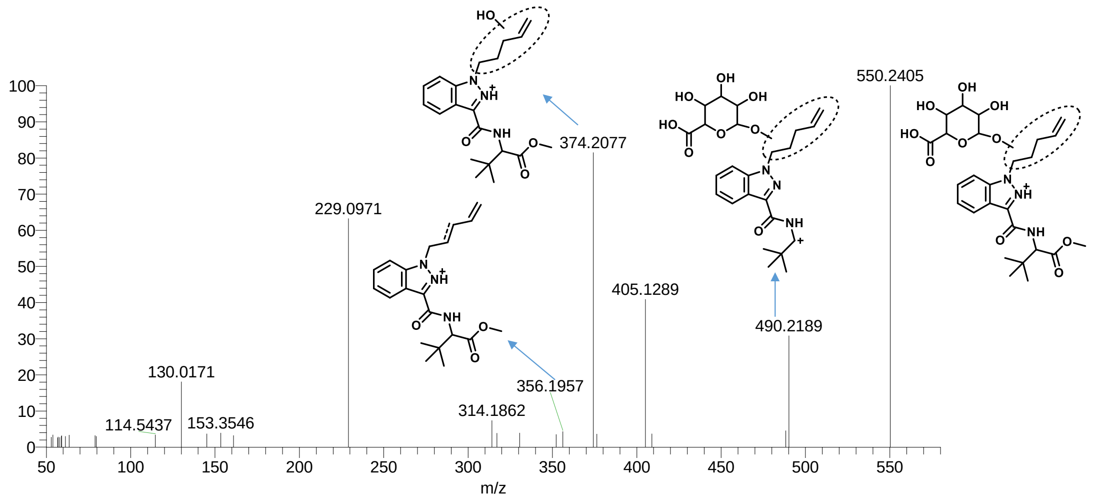

B28

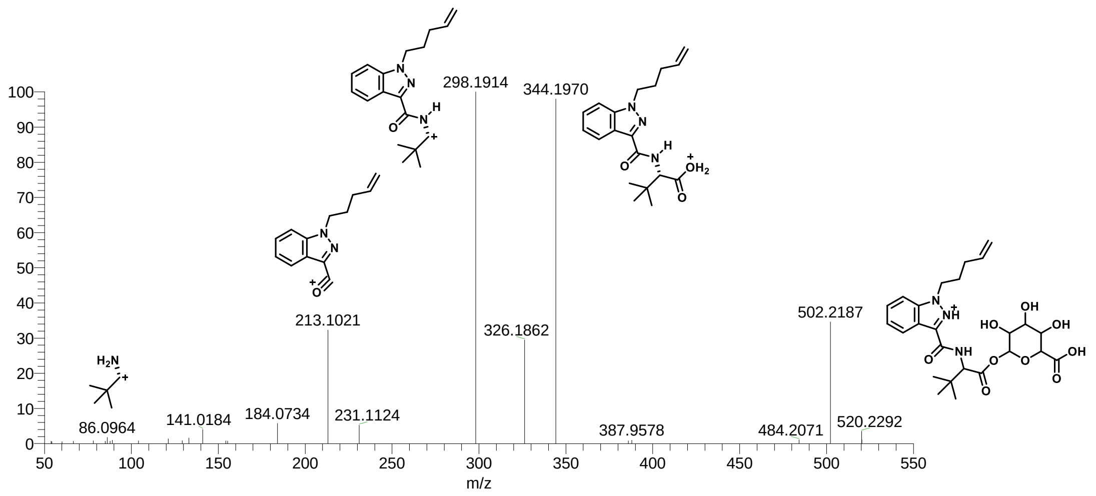

298.1914
344.1970
213.1021
326.1862
502.2187
86.0964
141.0184
184.0734
231.1124
387.9578
484.2071
520.2292
m/z

B29

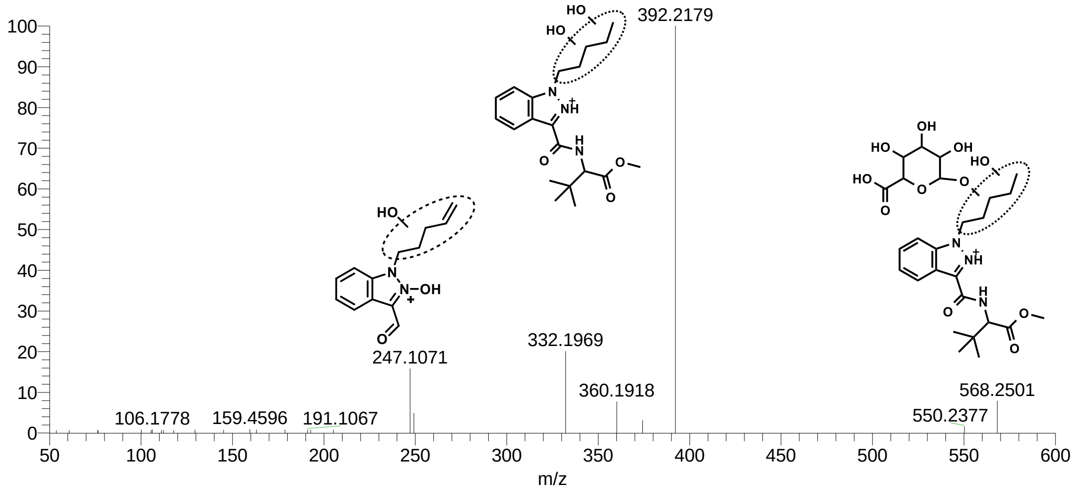

392.2179
332.1969
247.1071
360.1918
568.2501
550.2377
106.1778
159.4596
191.1067
m/z

Figure S9. Mass spectrometry spectra and fragmentation of BZO-HEXOXIZID and its metabolites.

C0

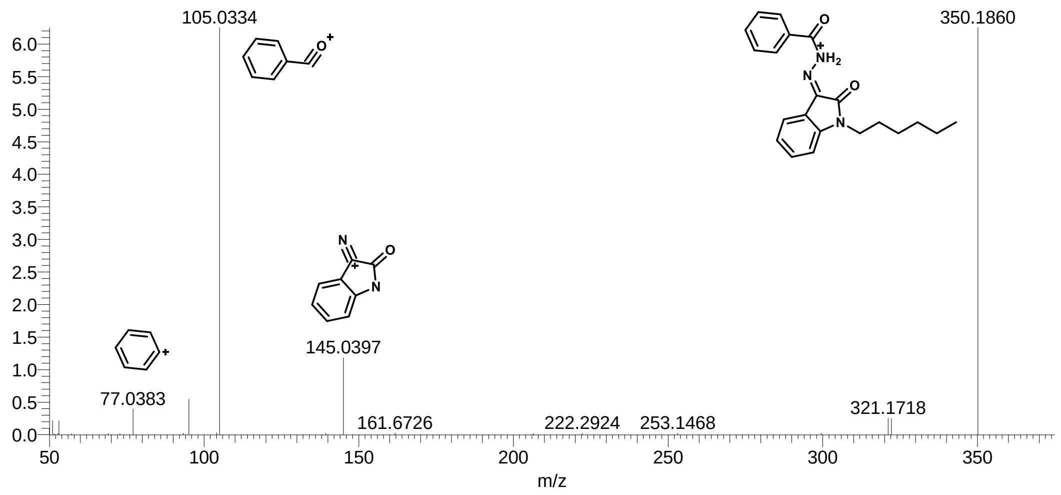

C1-3

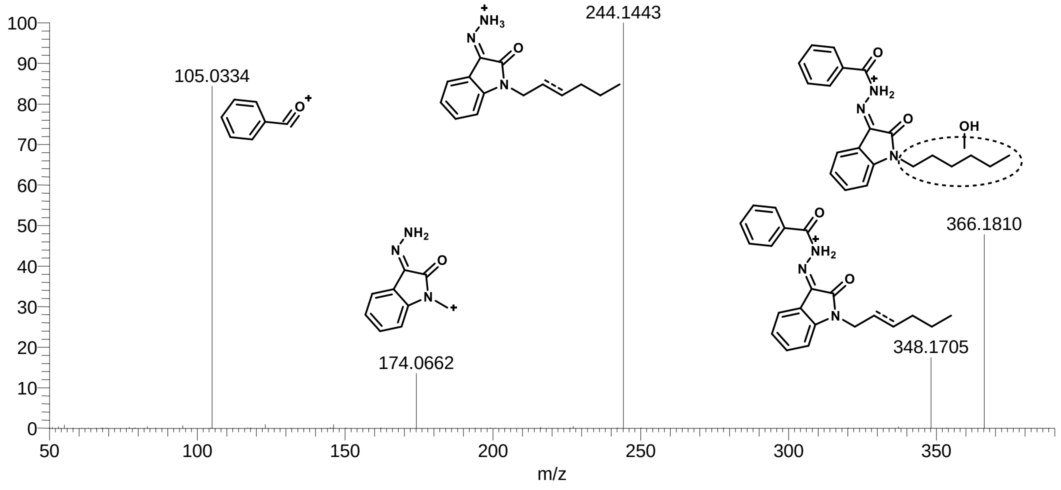

C4

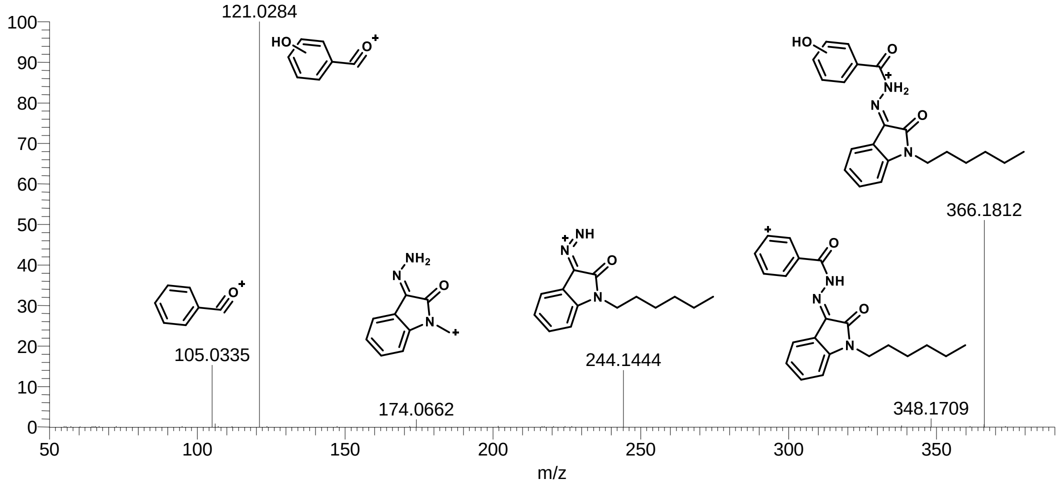

C5-7

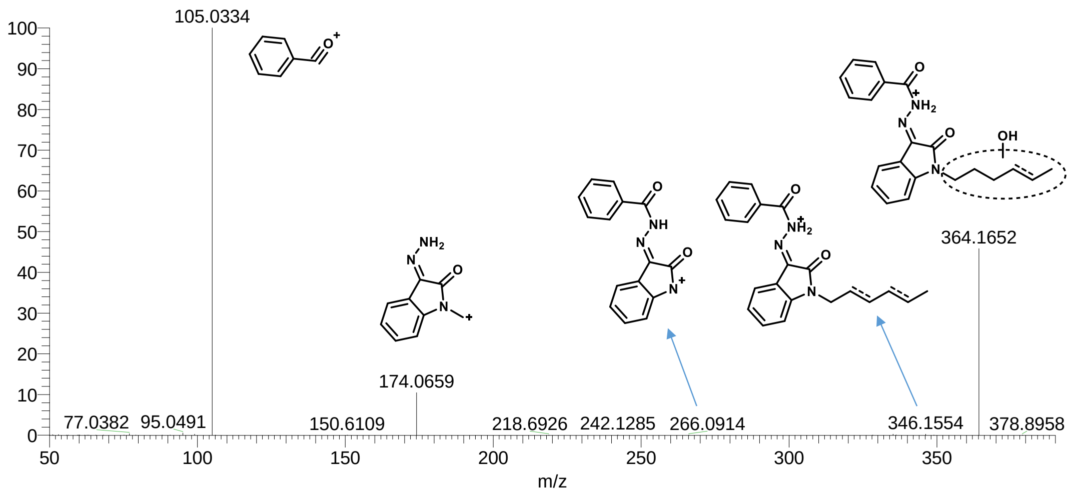

C8,9

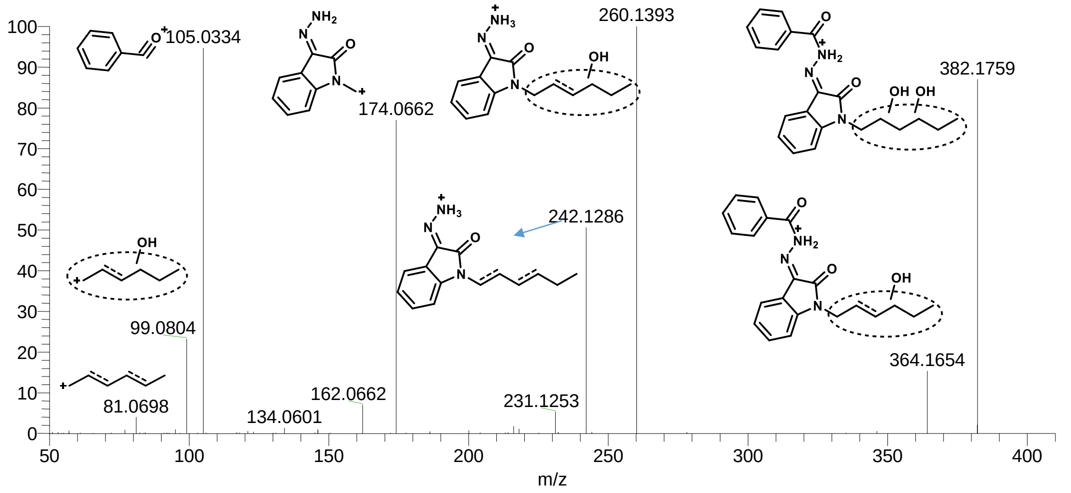

C10-12

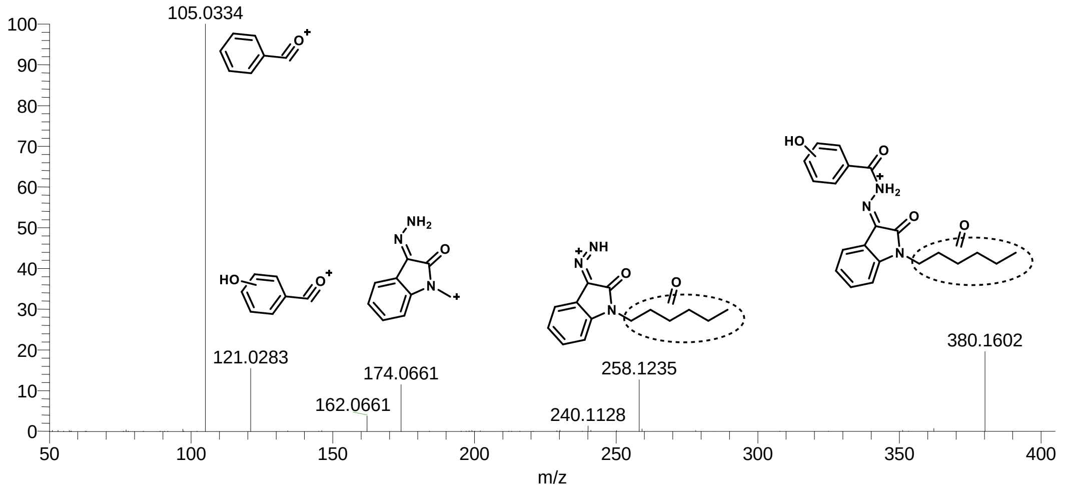

C13,14

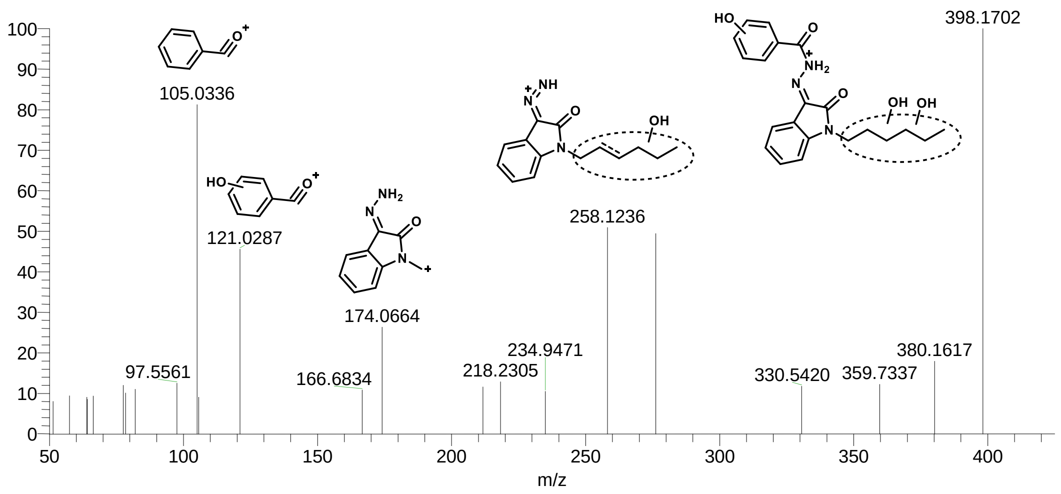

C15

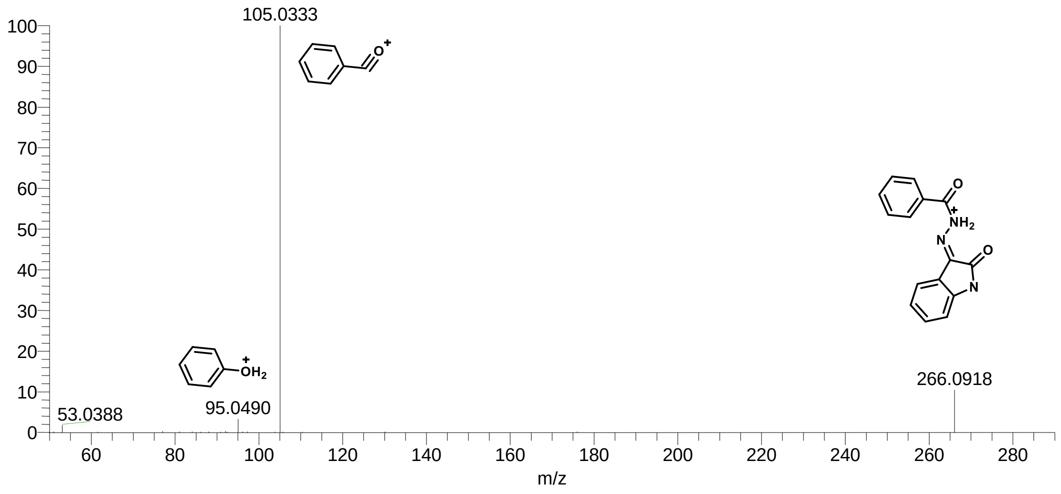

C16

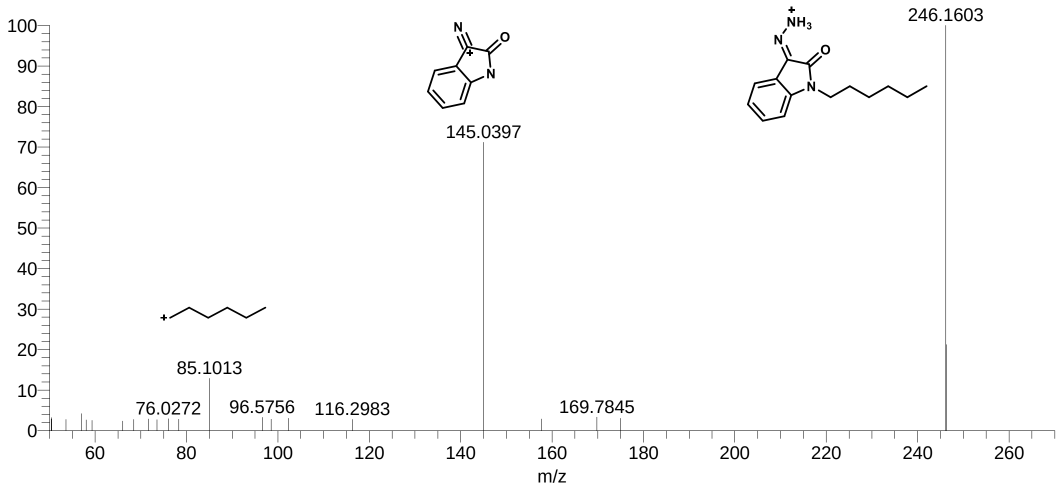

C17-20

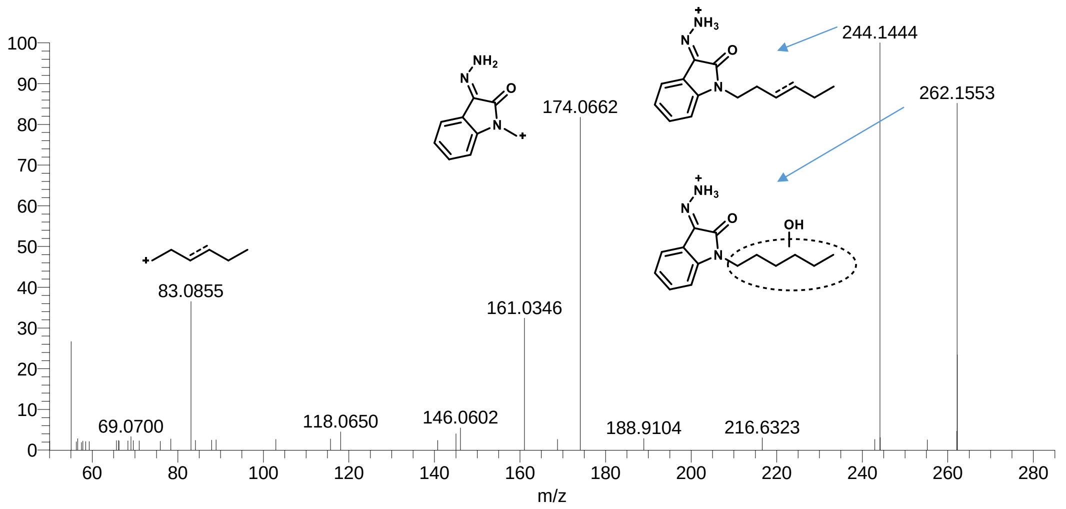

C21,22

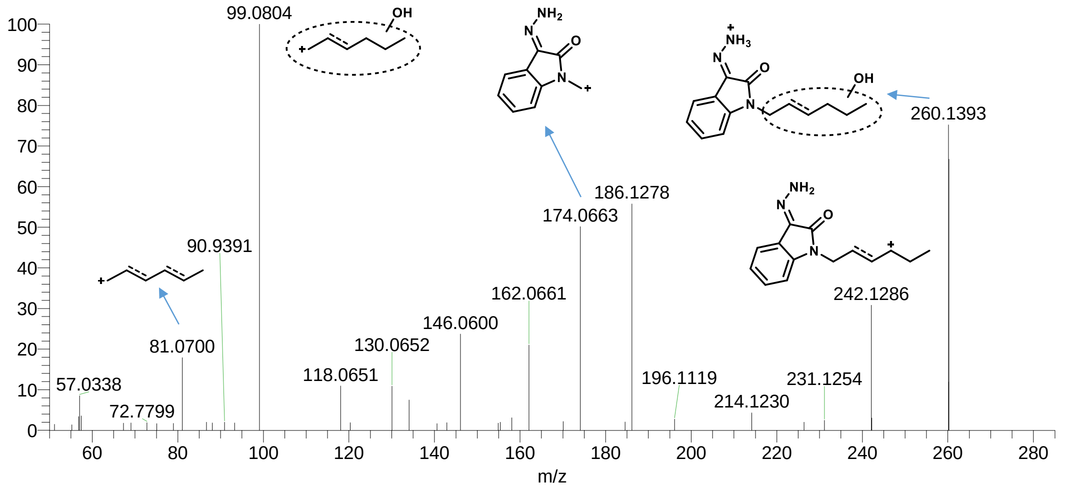

C23

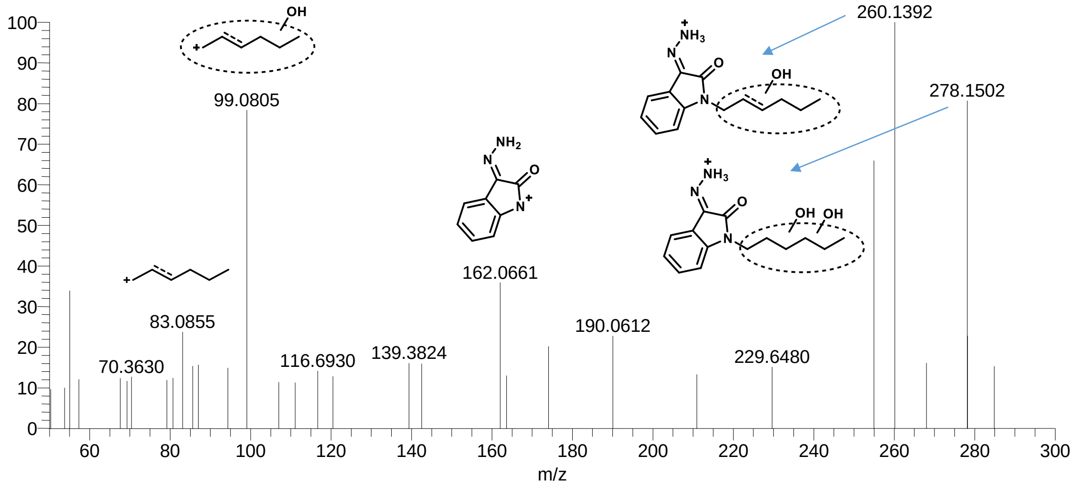

C24-26

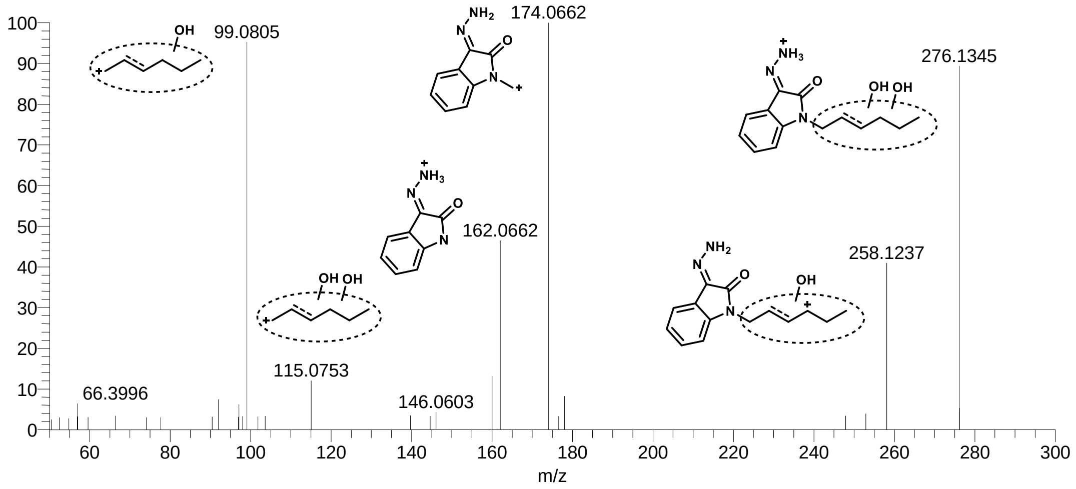

C27-30

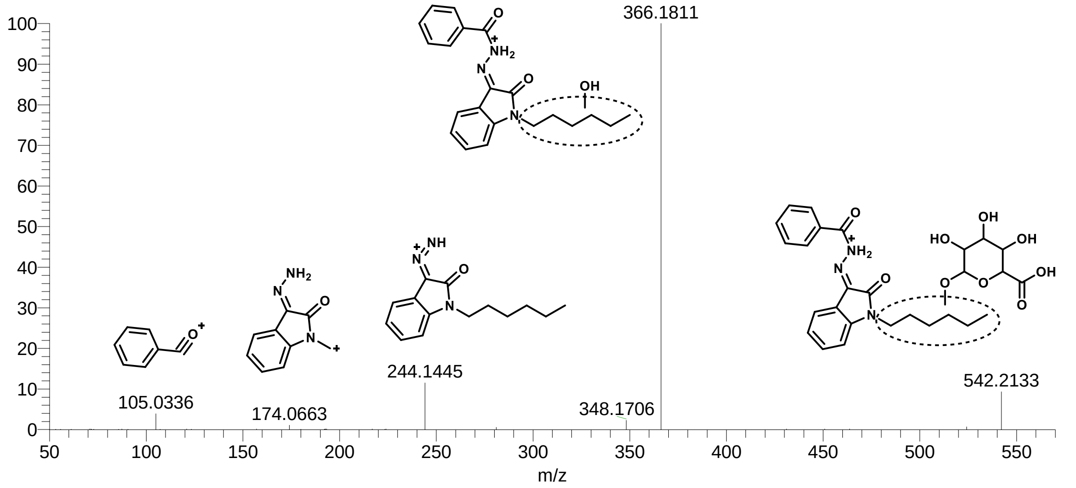

C31

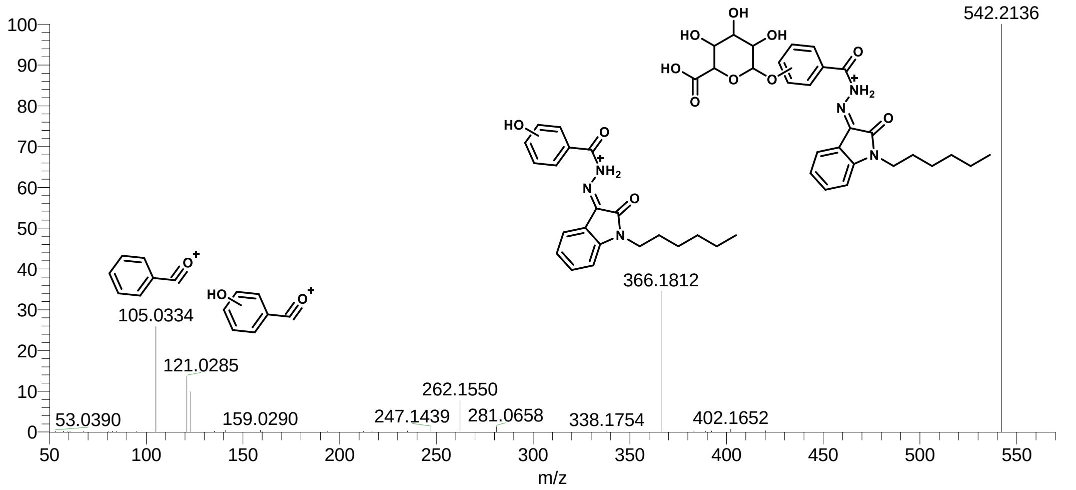

C32

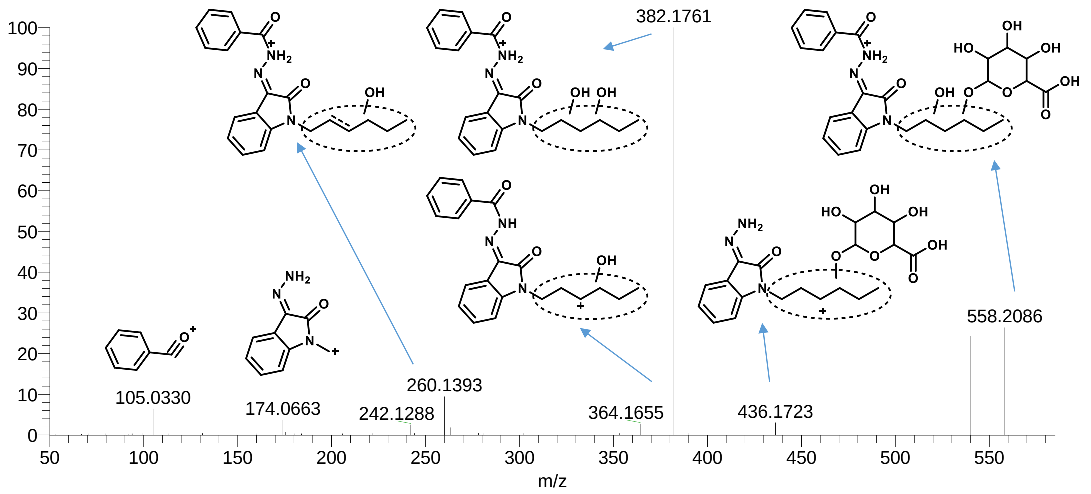
382.1761
558.2086
260.1393
105.0330
174.0663
242.1288
364.1655
436.1723
m/z

C33

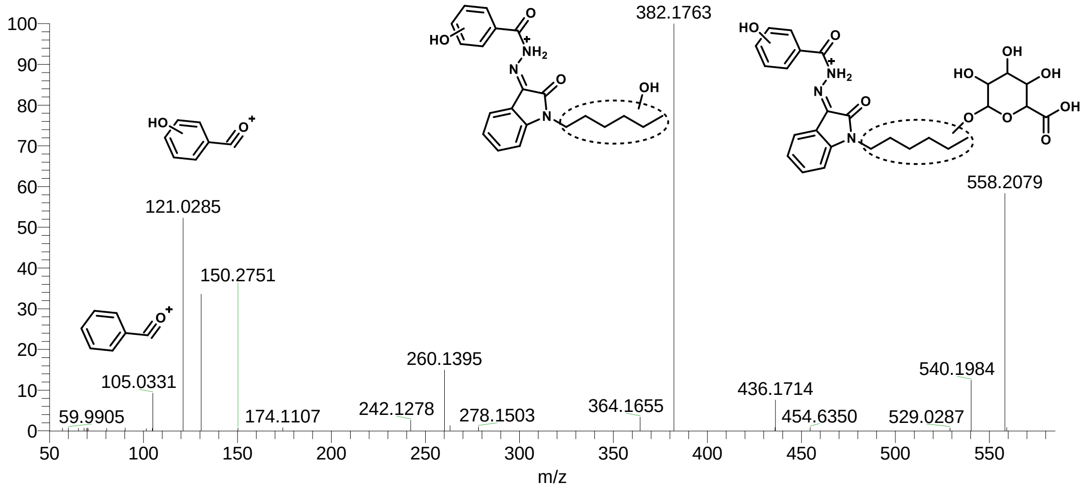
382.1763
558.2079
121.0285
150.2751
105.0331
59.9905
174.1107
242.1278
260.1395
278.1503
364.1655
436.1714
454.6350
529.0287
540.1984
m/z

C34

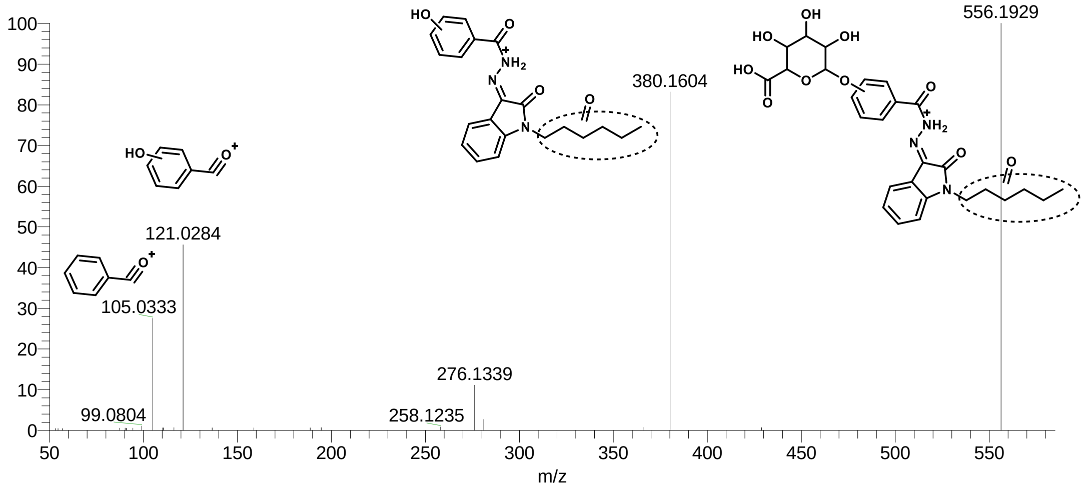
556.1929
380.1604
121.0284
105.0333
99.0804
258.1235
276.1339
m/z

Figure S10. Mass spectrometry spectra and fragmentation of BZO-POXIZID and its metabolites.

D0

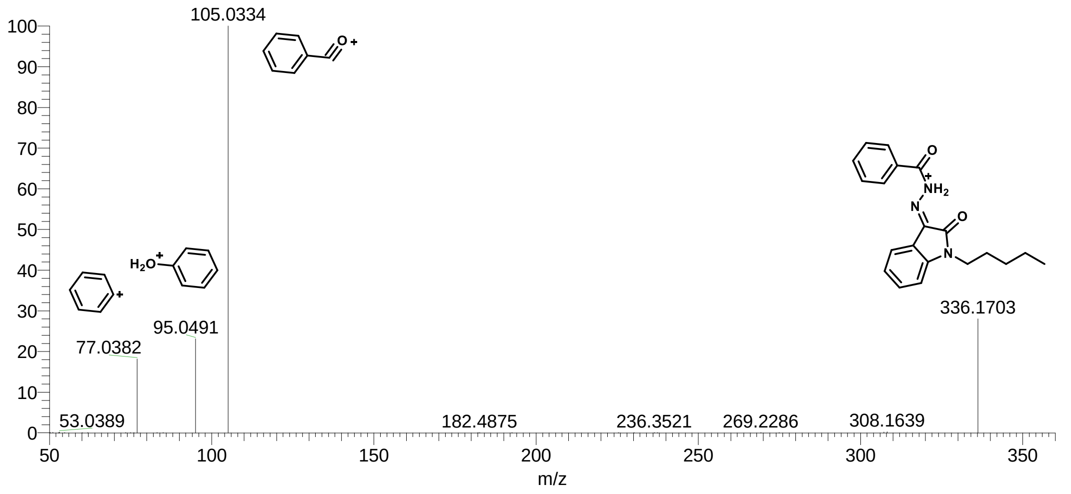

D1,2

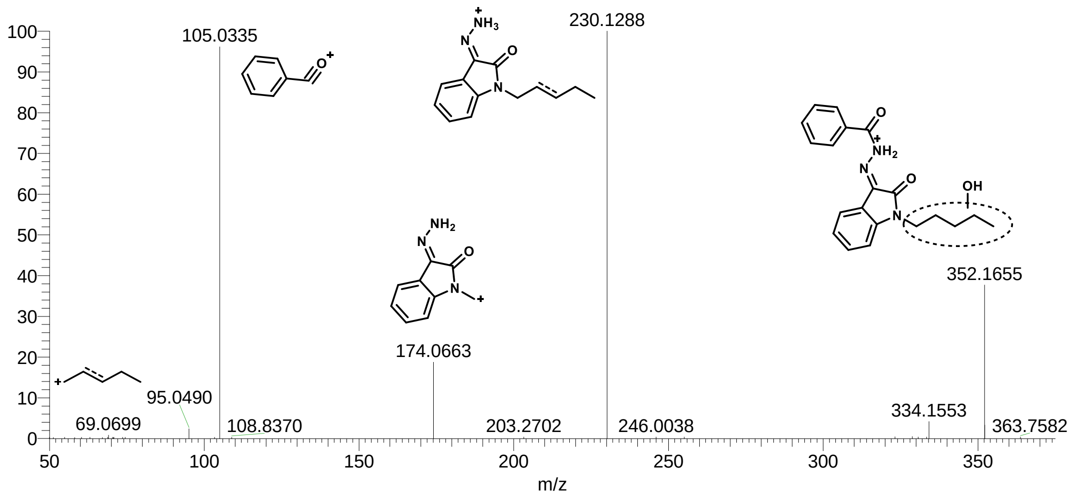

D3,4

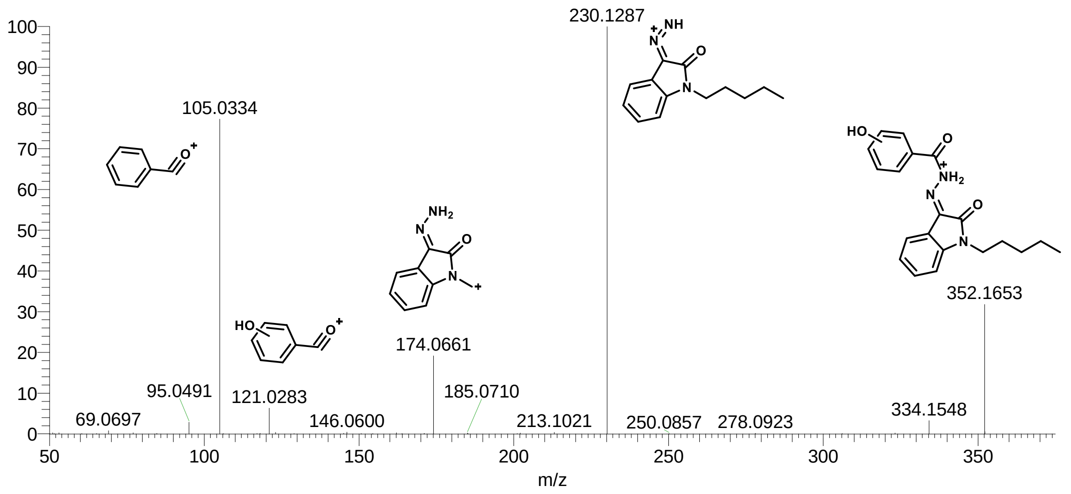

D5,6

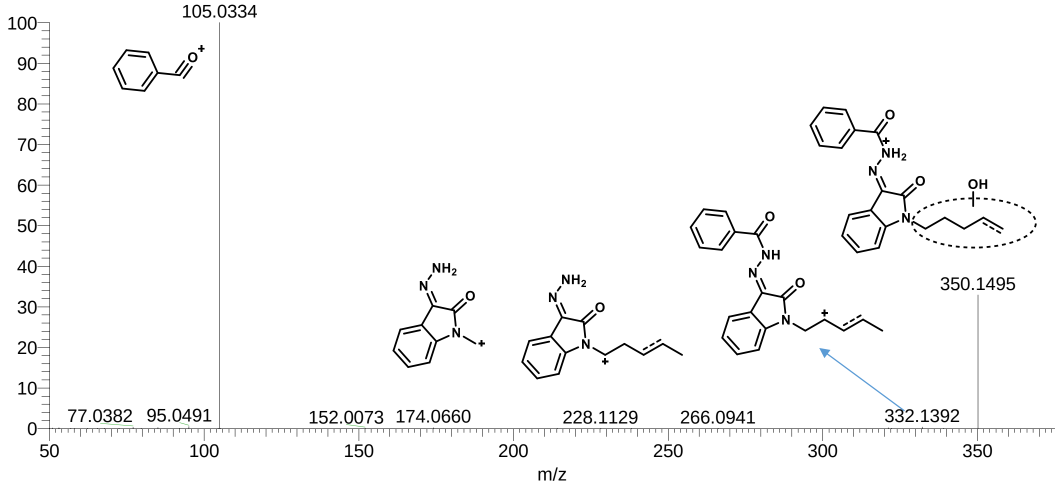
105.0334
350.1495
77.0382
95.0491
152.0073
174.0660
228.1129
266.0941
332.1392
m/z

D7,8

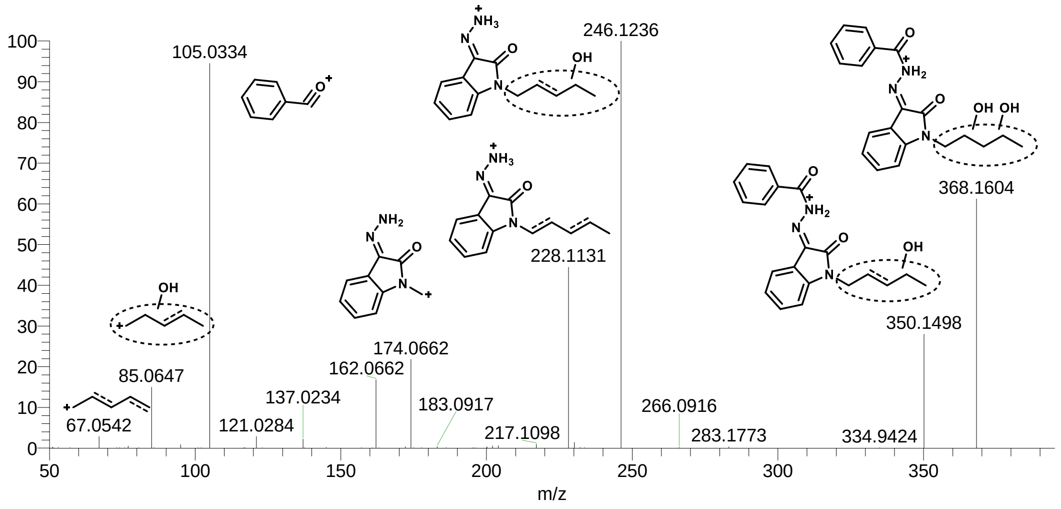
105.0334
246.1236
228.1131
368.1604
350.1498
85.0647
67.0542
121.0284
137.0234
162.0662
174.0662
183.0917
217.1098
266.0916
283.1773
334.9424
m/z

D9-11

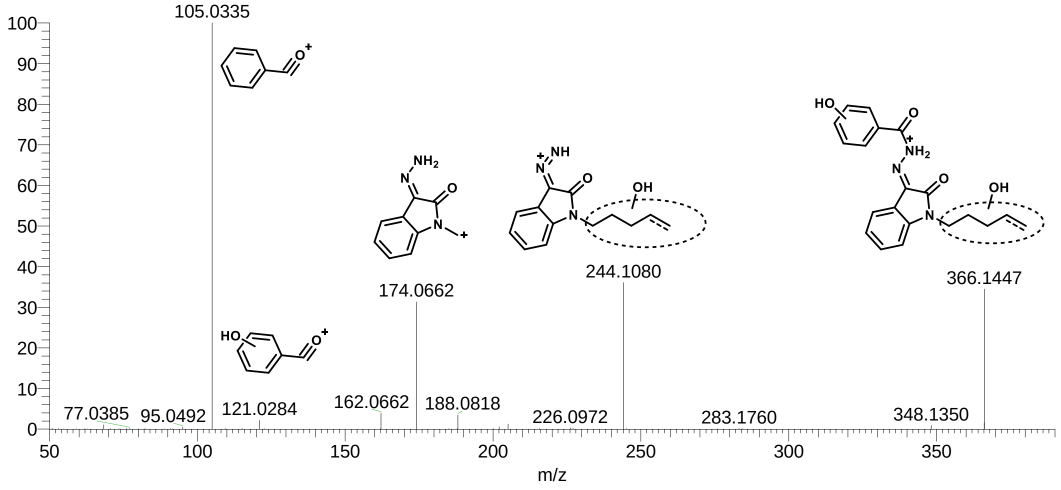
105.0335
174.0662
244.1080
366.1447
77.0385
95.0492
121.0284
162.0662
188.0818
226.0972
283.1760
348.1350
m/z

D12

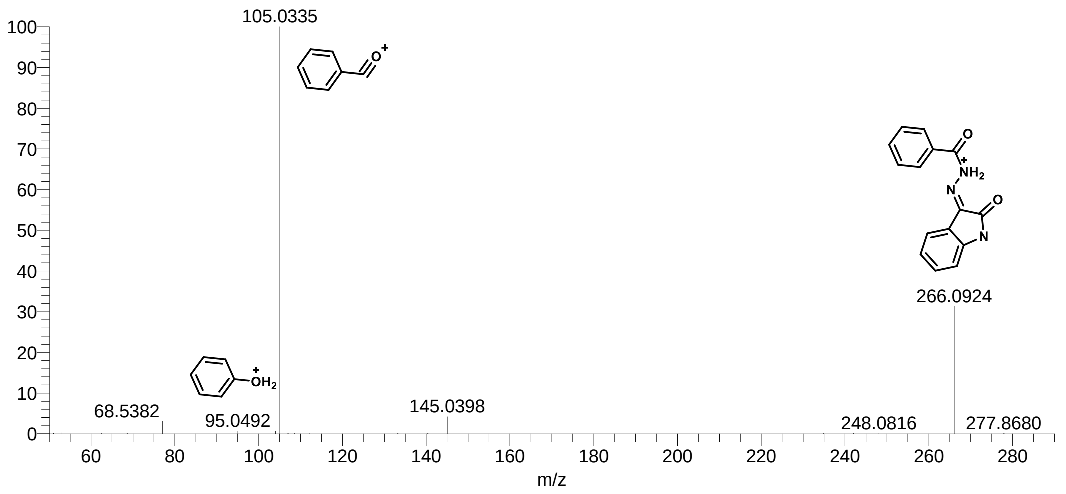

D13-15

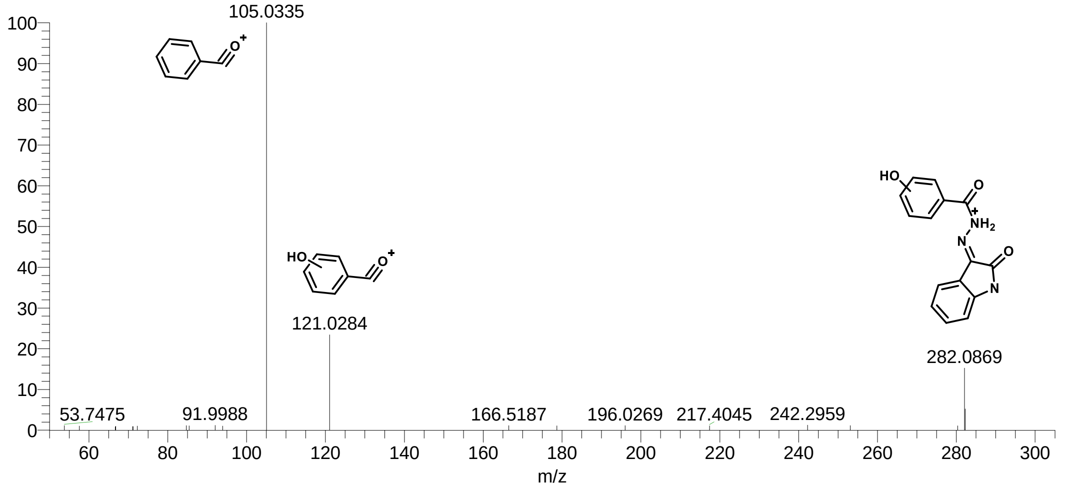

D16

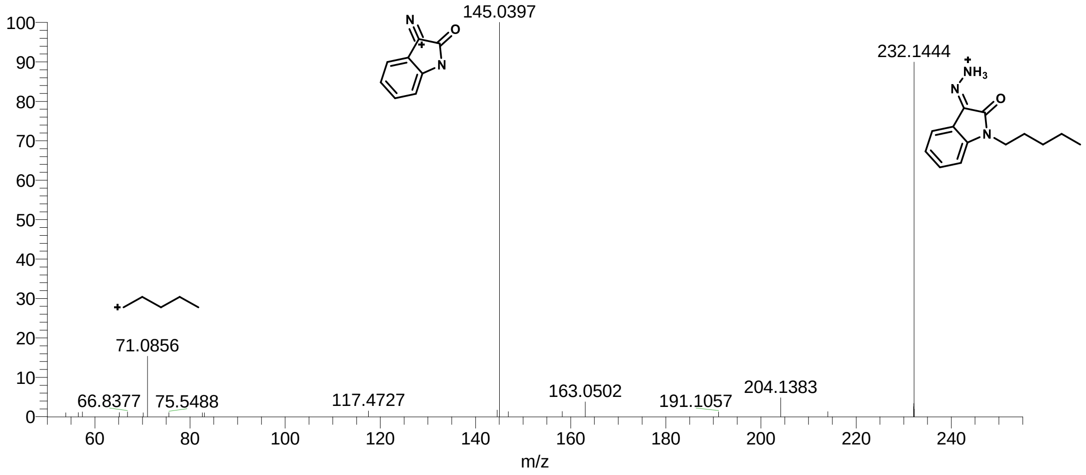

D17-20

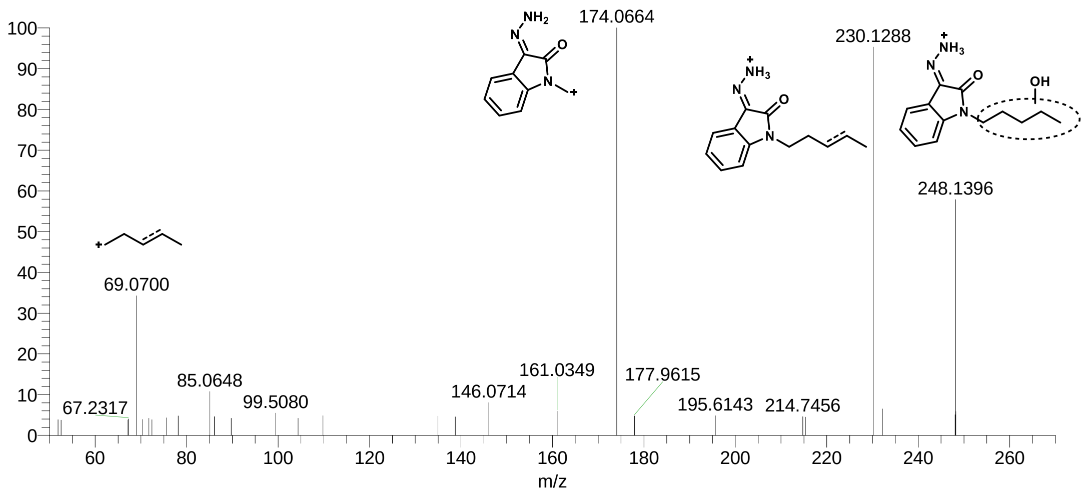

D21-25

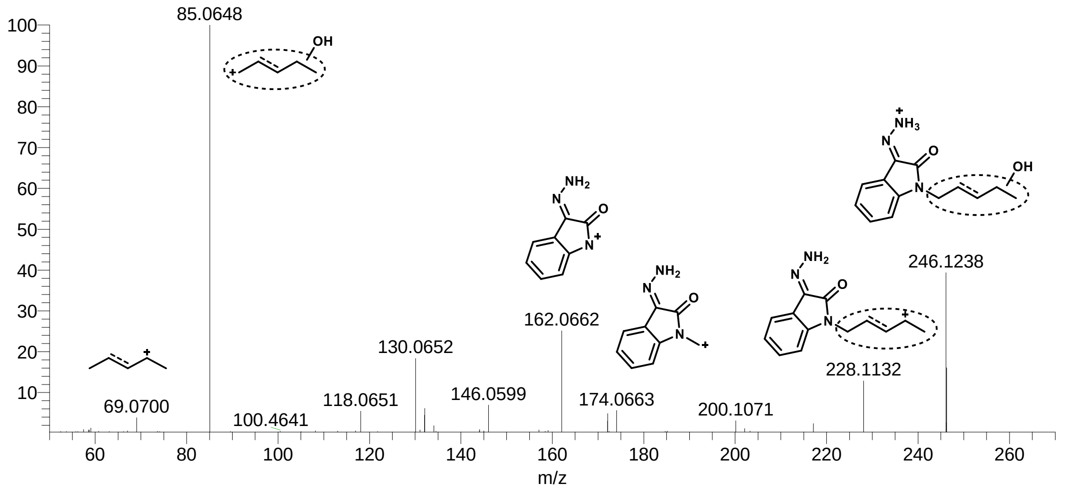

D26

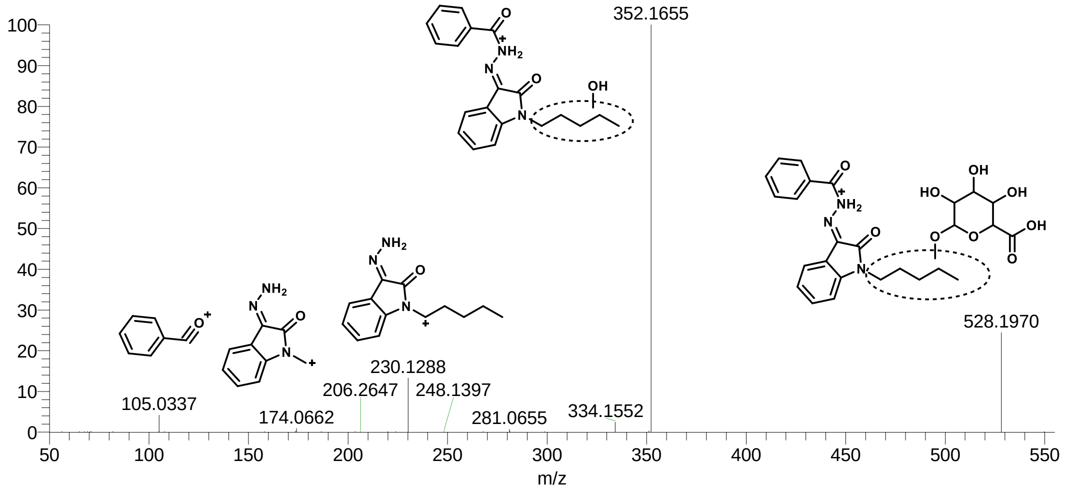

D27,28

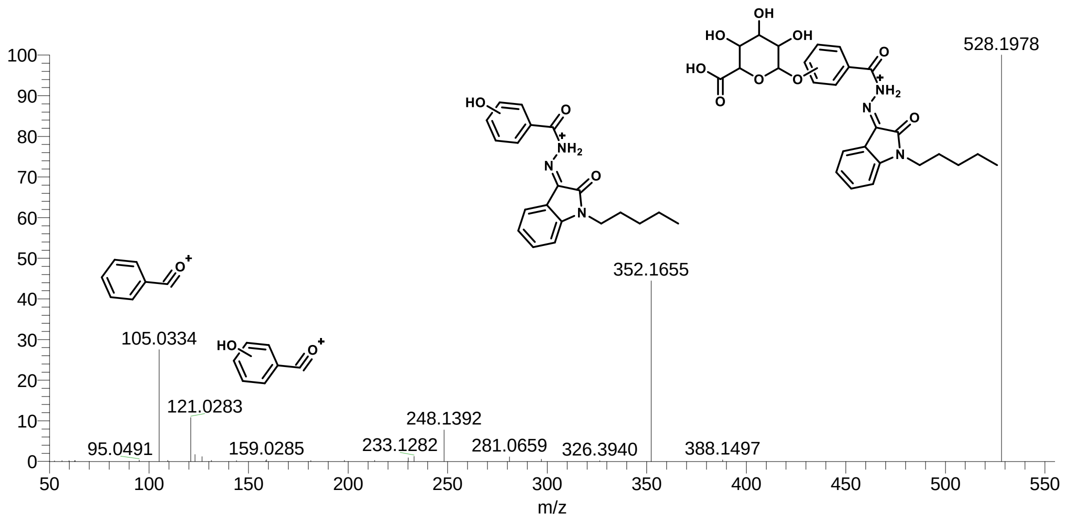
528.1978
352.1655
105.0334
121.0283
95.0491
159.0285
233.1282
248.1392
281.0659
326.3940
388.1497
m/z
HO
OH
HO
O
NH2
N
N
O

D29,30

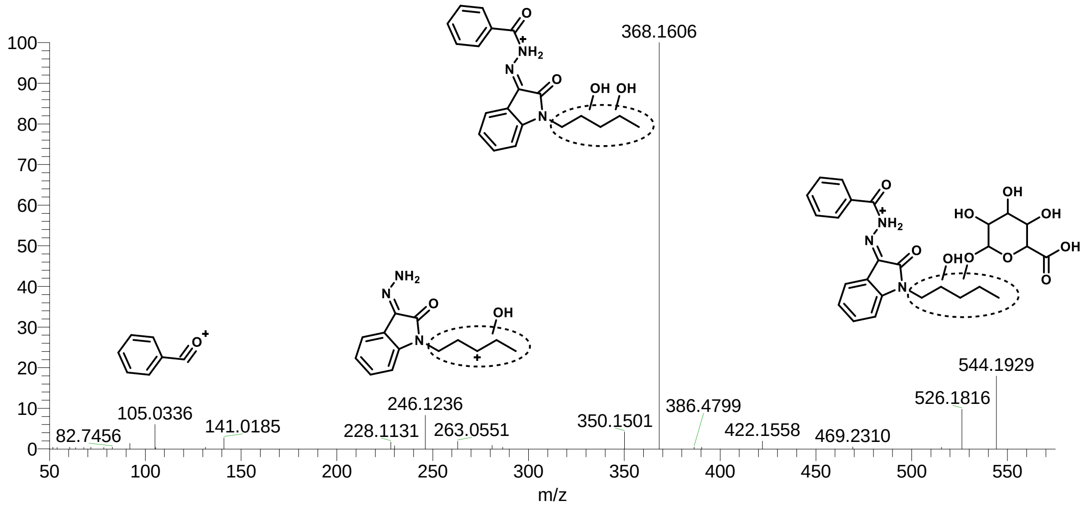
368.1606
544.1929
526.1816
105.0336
141.0185
82.7456
228.1131
246.1236
263.0551
350.1501
386.4799
422.1558
469.2310
m/z
OH
NH2
N
O

Figure S11. Mass spectrometry spectra and fragmentation of BZO-4en-POXIZID and its metabolites.

E0

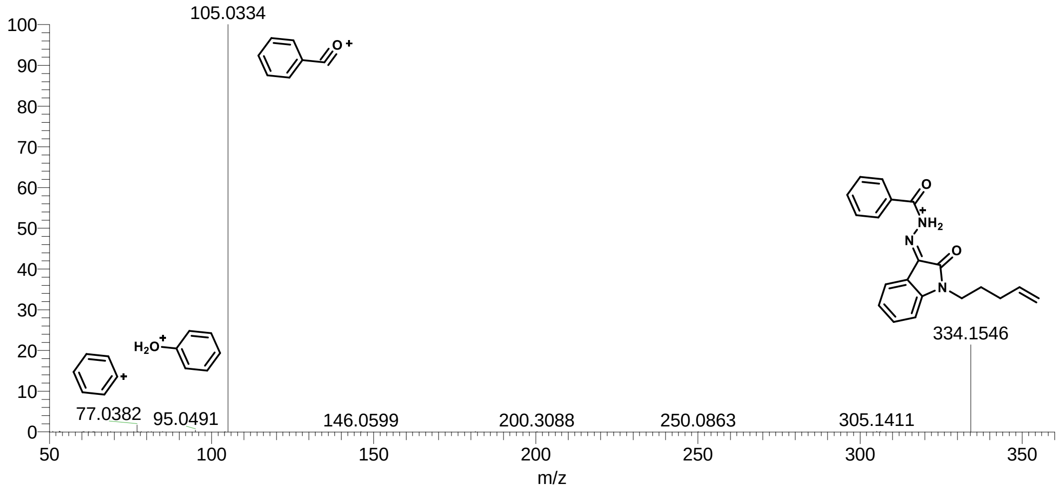

E1,2,3

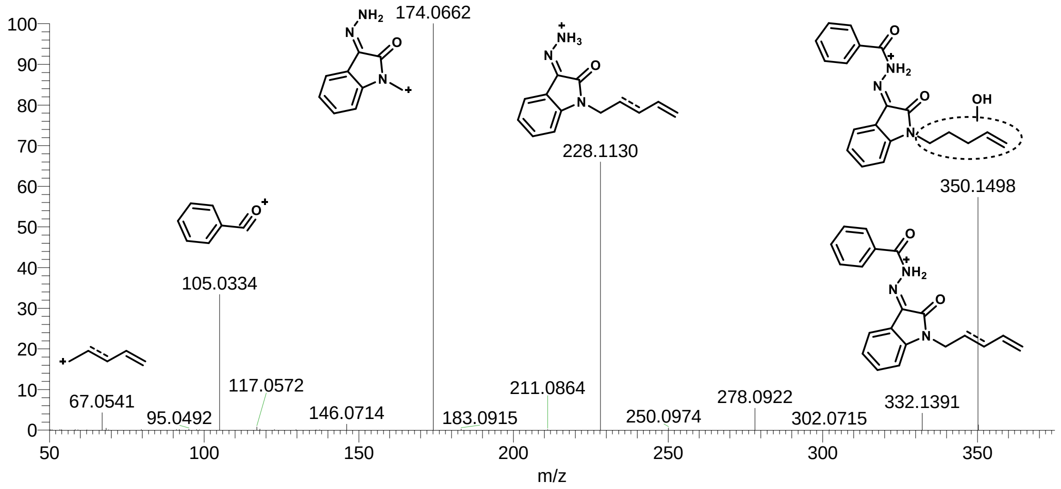

E4

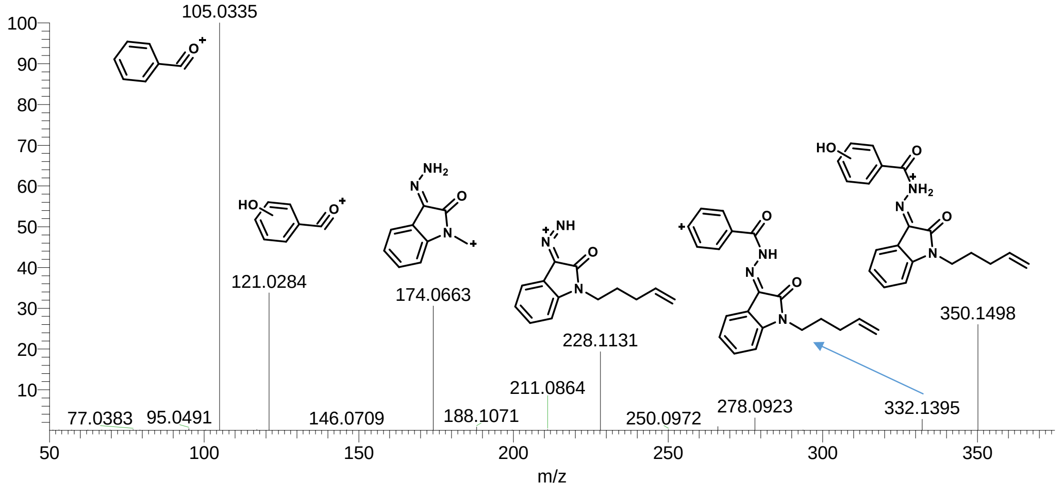

E5,6

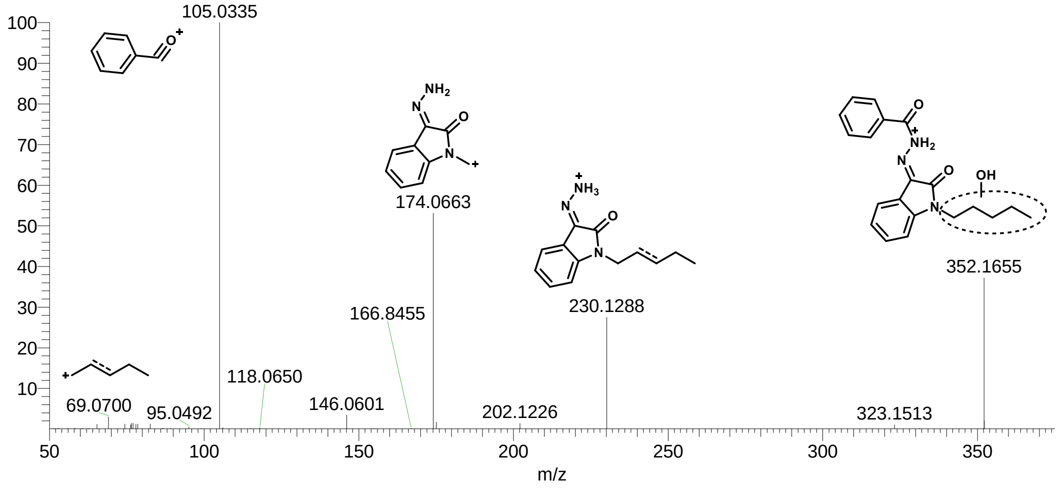
105.0335
174.0663
230.1288
352.1655
166.8455
118.0650
69.0700
95.0492
146.0601
202.1226
323.1513
m/z

E7

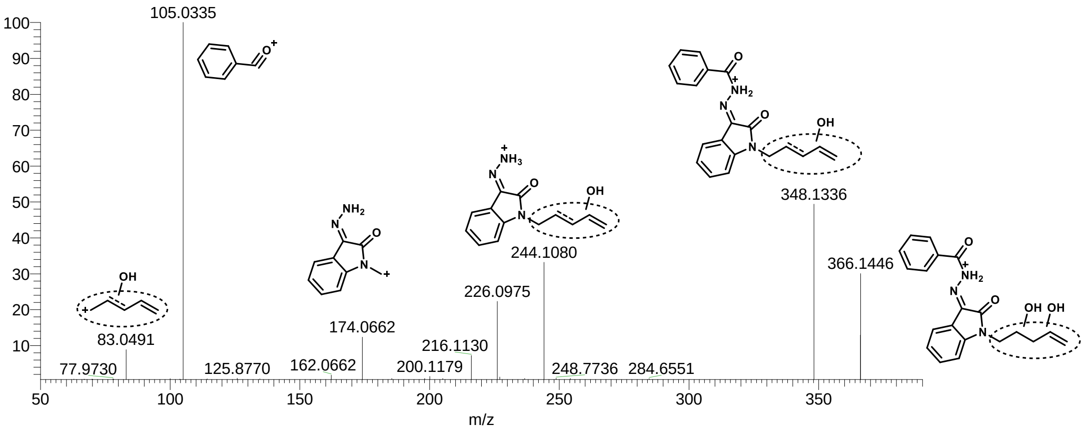
105.0335
348.1336
244.1080
366.1446
226.0975
174.0662
83.0491
216.1130
77.9730
125.8770
162.0662
200.1179
248.7736
284.6551
m/z

E8

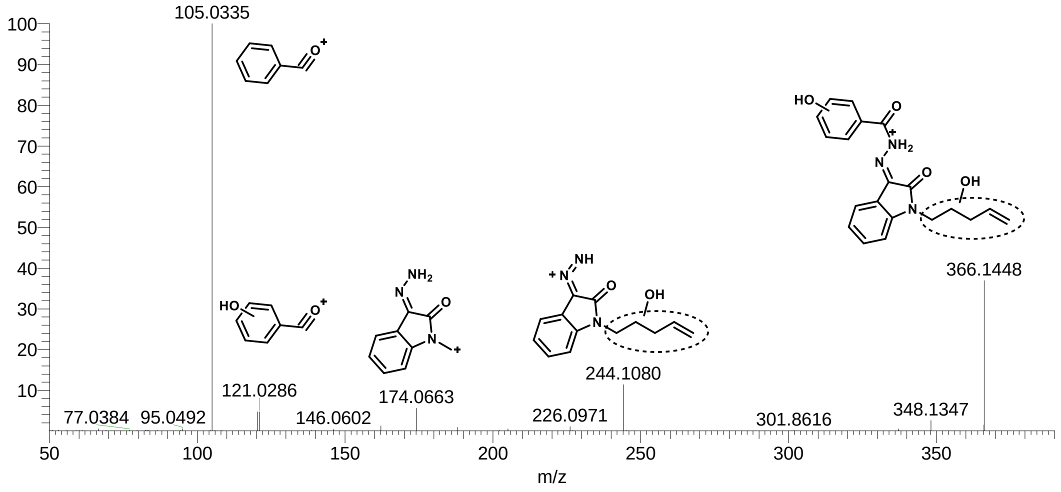
105.0335
366.1448
244.1080
121.0286
174.0663
77.0384
95.0492
146.0602
226.0971
301.8616
348.1347
m/z

E9

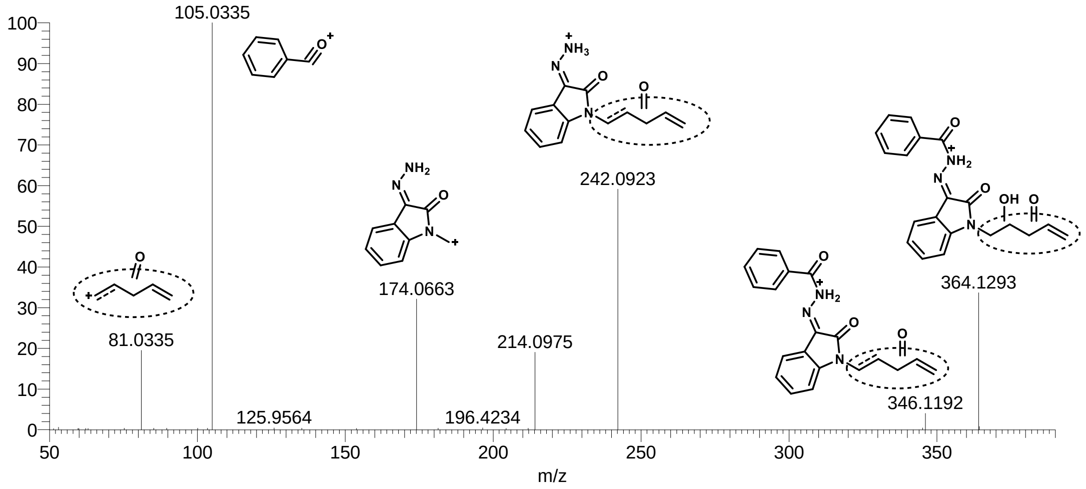
105.0335
242.0923
364.1293
81.0335
174.0663
214.0975
346.1192
125.9564
196.4234
m/z

E10

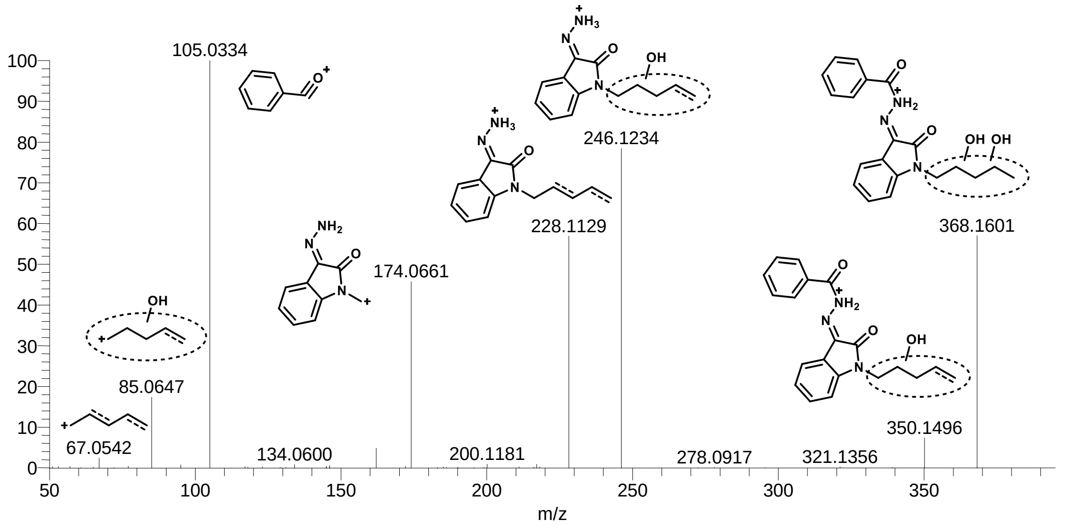
105.0334
246.1234
228.1129
368.1601
174.0661
85.0647
67.0542
350.1496
134.0600
200.1181
278.0917
321.1356
m/z

E11

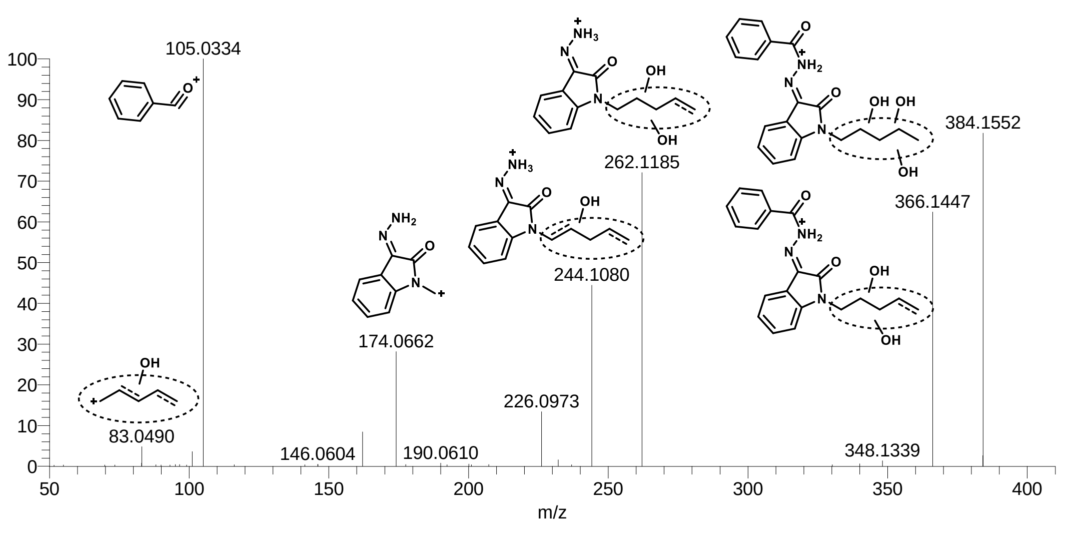
105.0334
262.1185
384.1552
366.1447
244.1080
174.0662
226.0973
83.0490
146.0604
190.0610
348.1339
m/z

E12

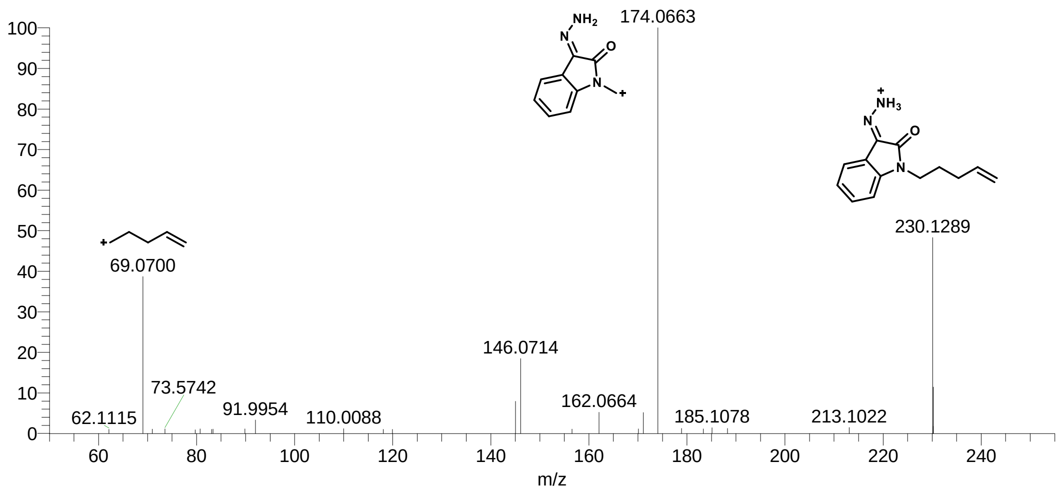

E13

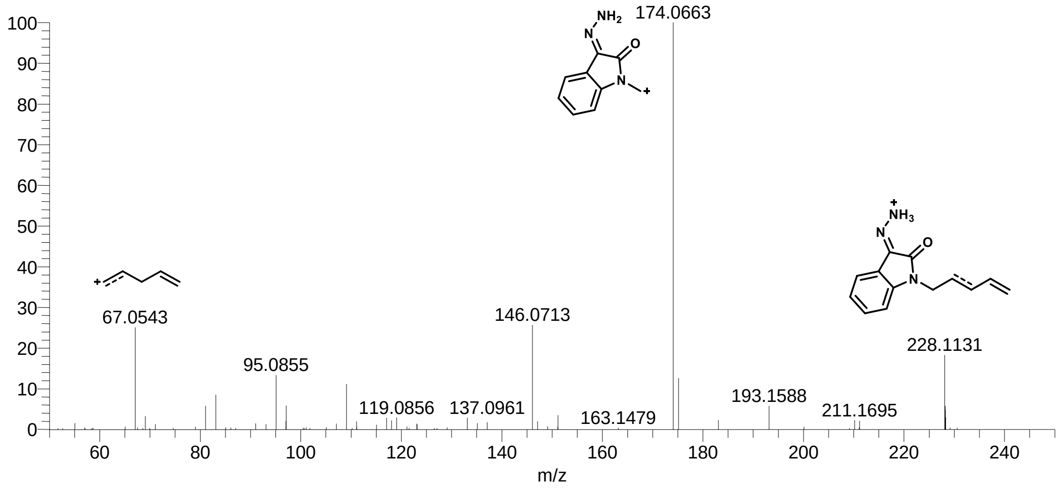

E14-16

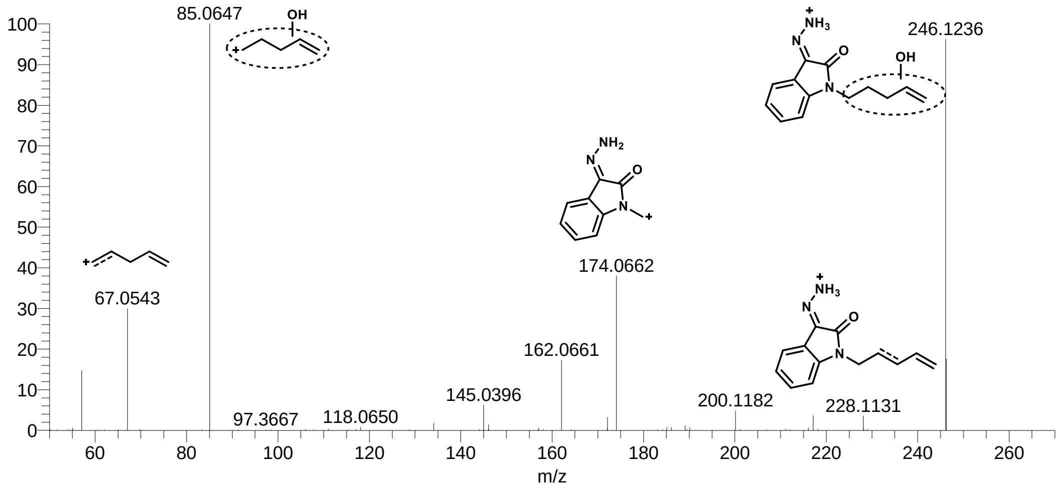

E17-19

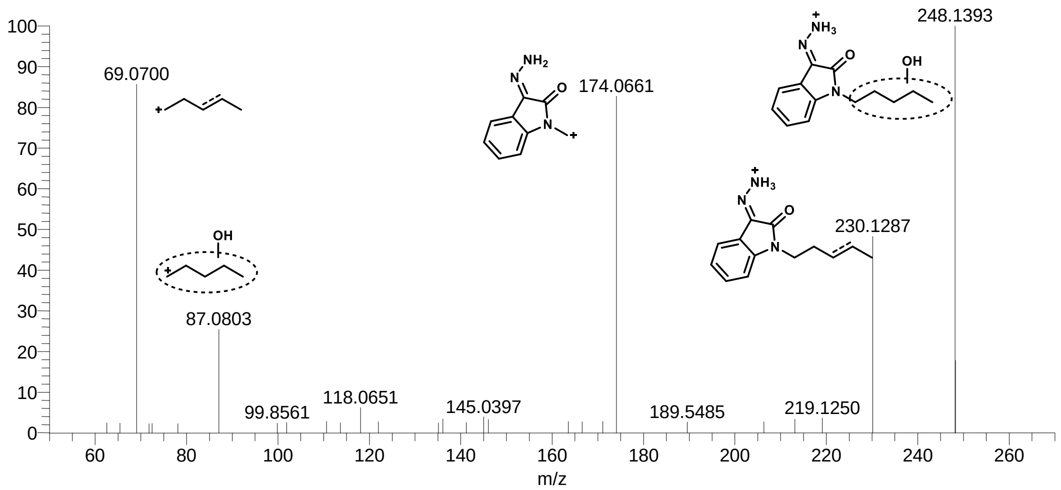

E20,21

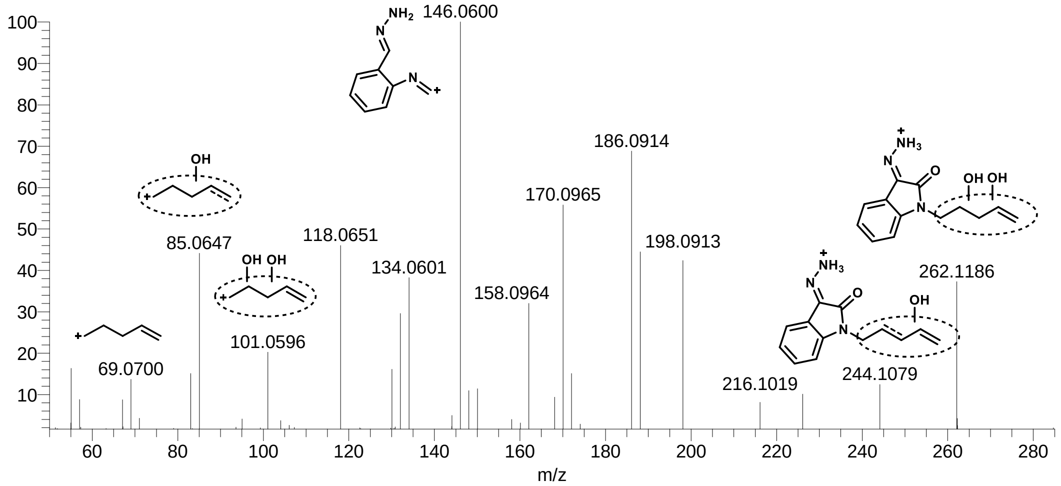

E22,23

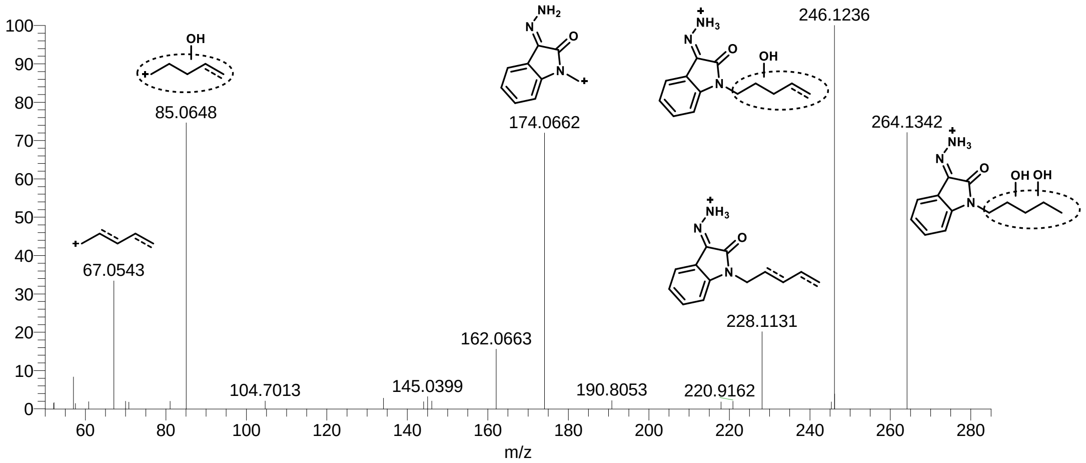

E24

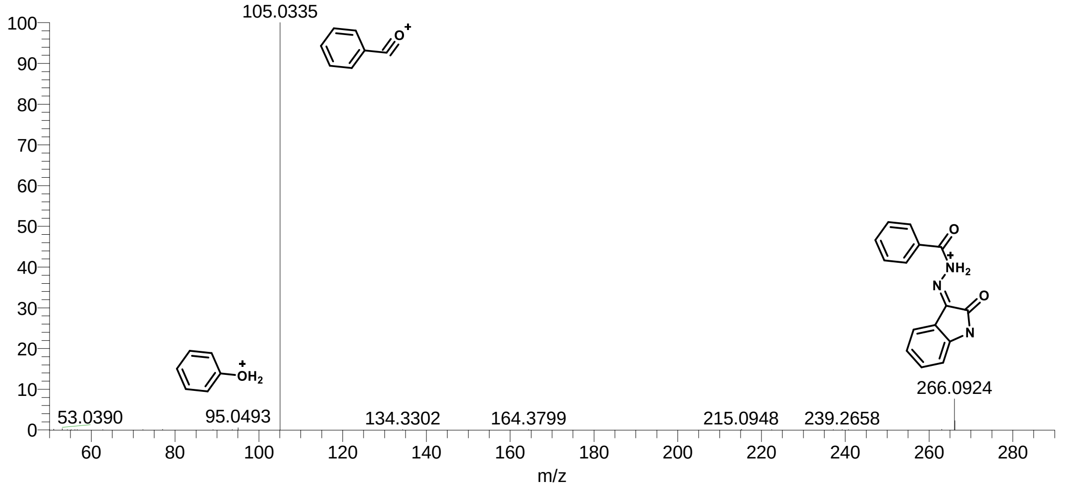
105.0335
53.0390
95.0493
134.3302
164.3799
215.0948
239.2658
266.0924
m/z

E25

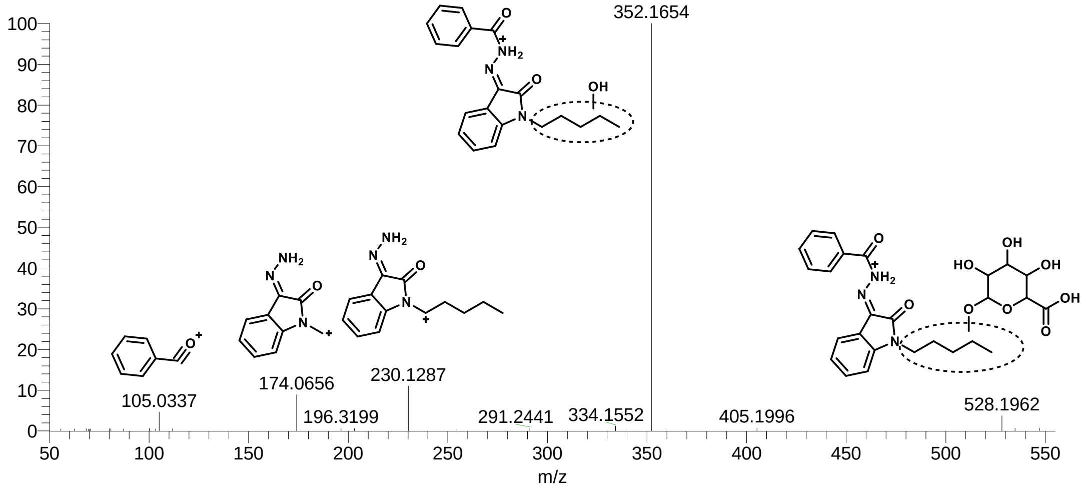
352.1654
105.0337
174.0656
196.3199
230.1287
291.2441
334.1552
405.1996
528.1962
m/z

E26

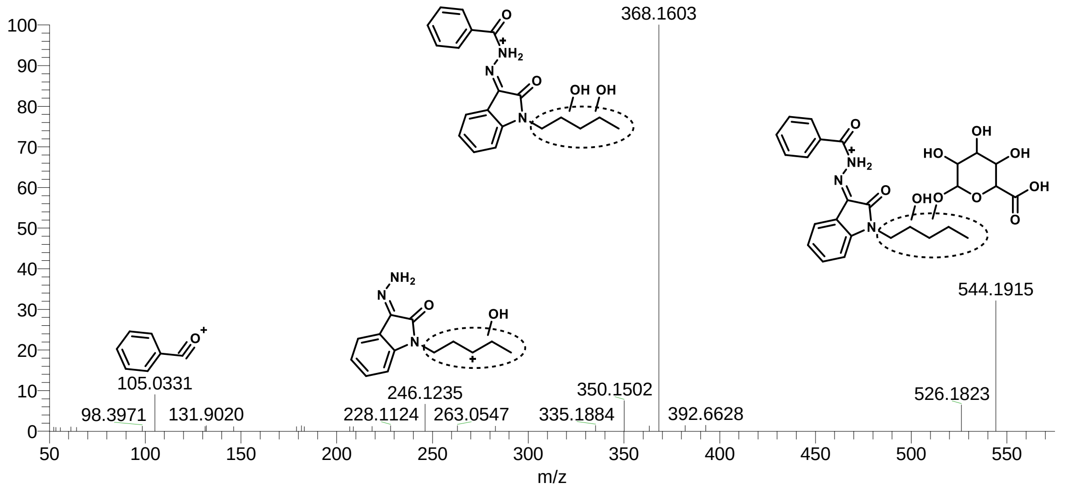
368.1603
544.1915
105.0331
98.3971
131.9020
228.1124
246.1235
263.0547
335.1884
350.1502
392.6628
526.1823
m/z

Figure S12. Mass spectrometry spectra and fragmentation of 5F-BZO-POXIZID and its metabolites.

F0

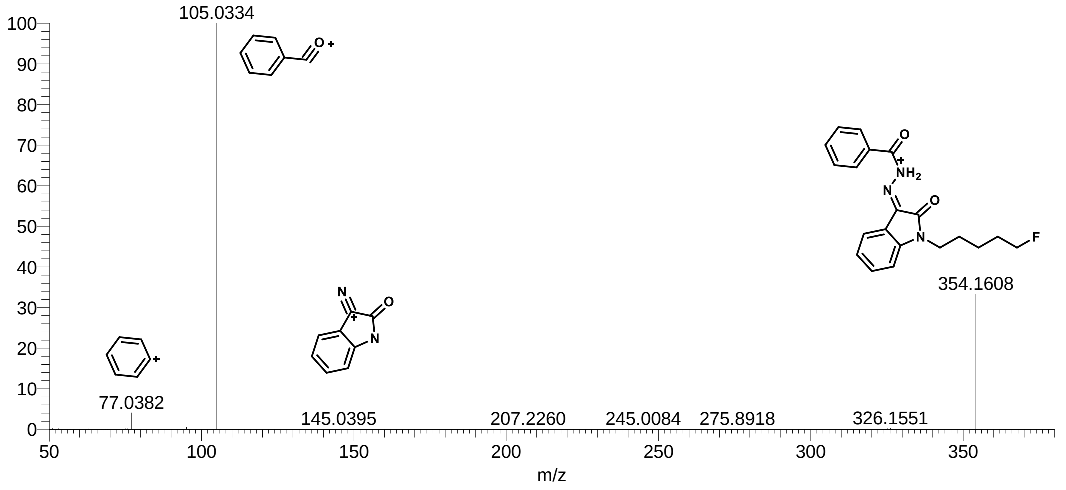

F1,2

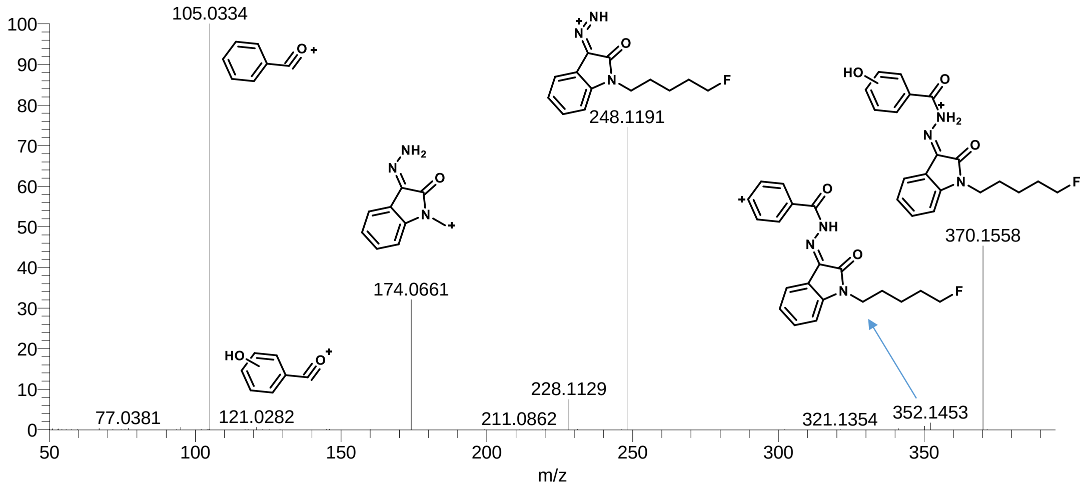

F3-5

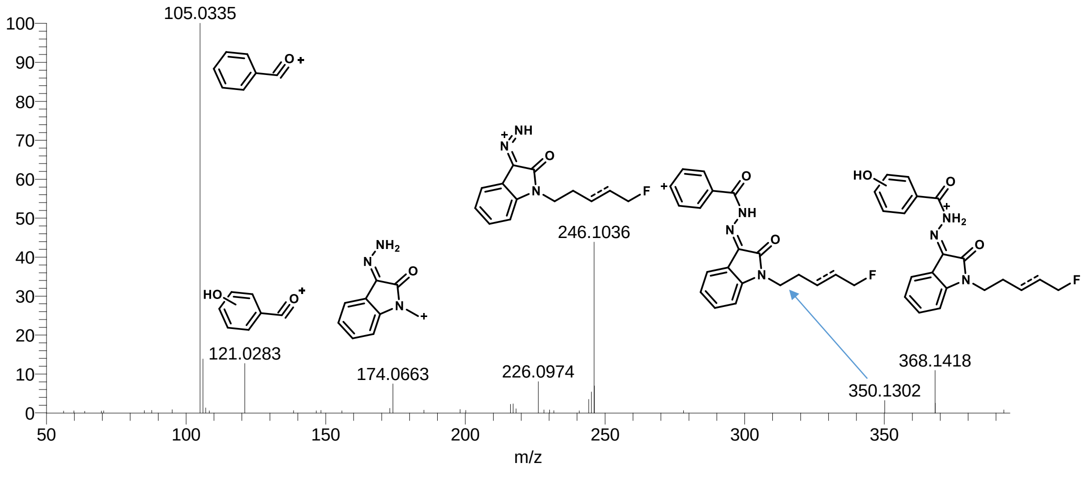

F6,7

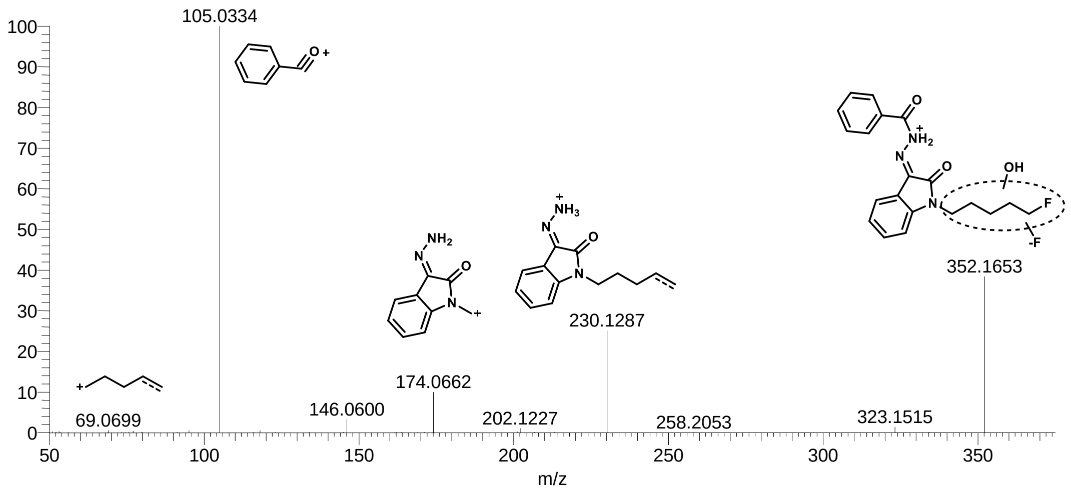
105.0334
352.1653
230.1287
174.0662
146.0600
202.1227
258.2053
323.1515
69.0699
m/z

F8-10

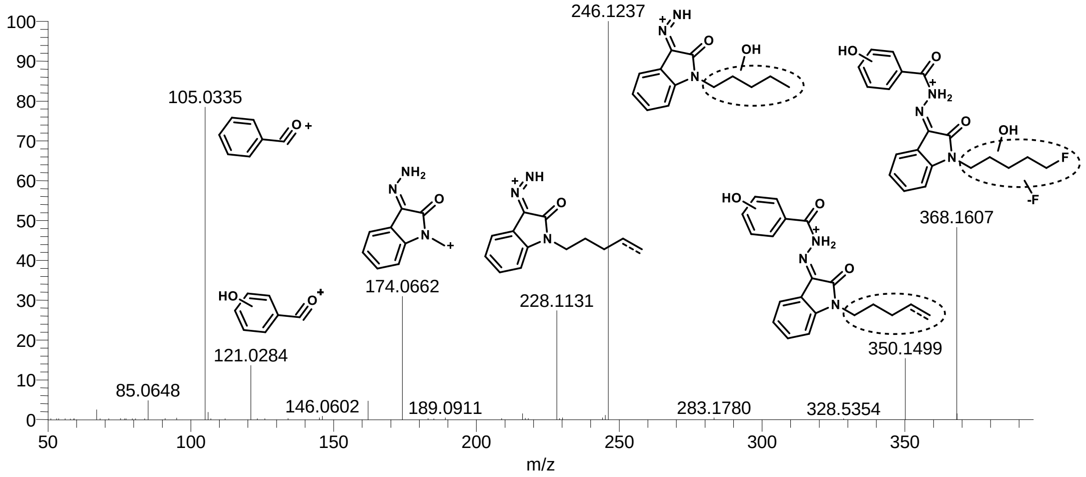
246.1237
105.0335
368.1607
174.0662
228.1131
121.0284
350.1499
85.0648
146.0602
189.0911
283.1780
328.5354
m/z

F11

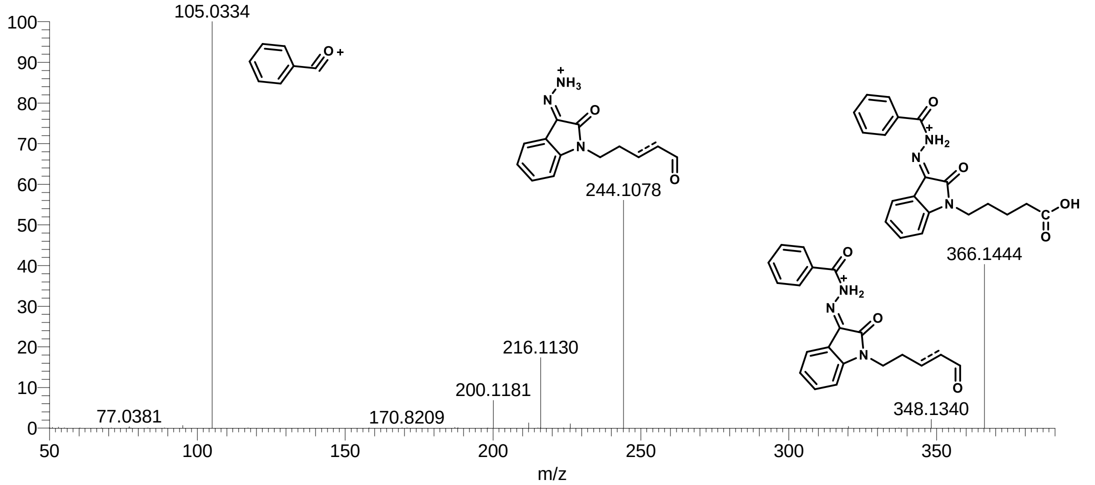
105.0334
244.1078
366.1444
216.1130
200.1181
348.1340
77.0381
170.8209
m/z

F12

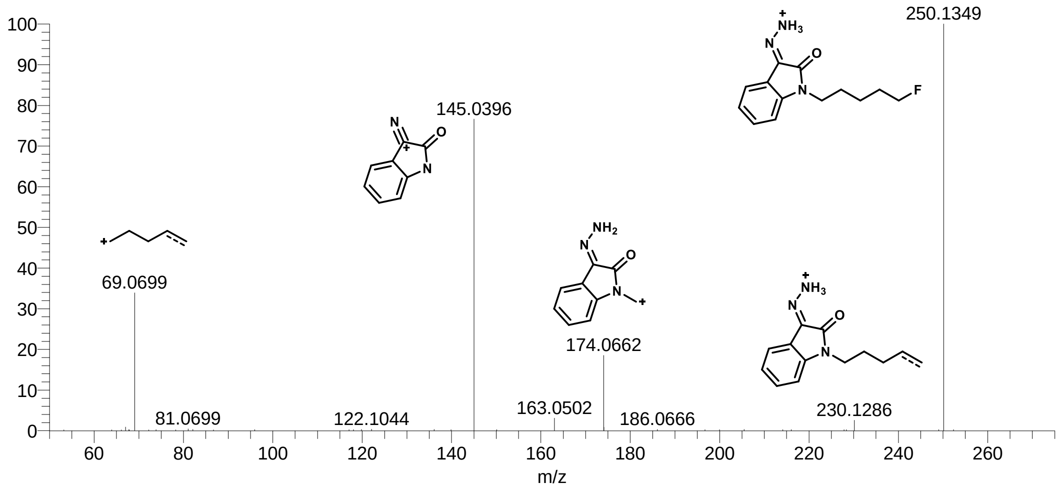

F13-15

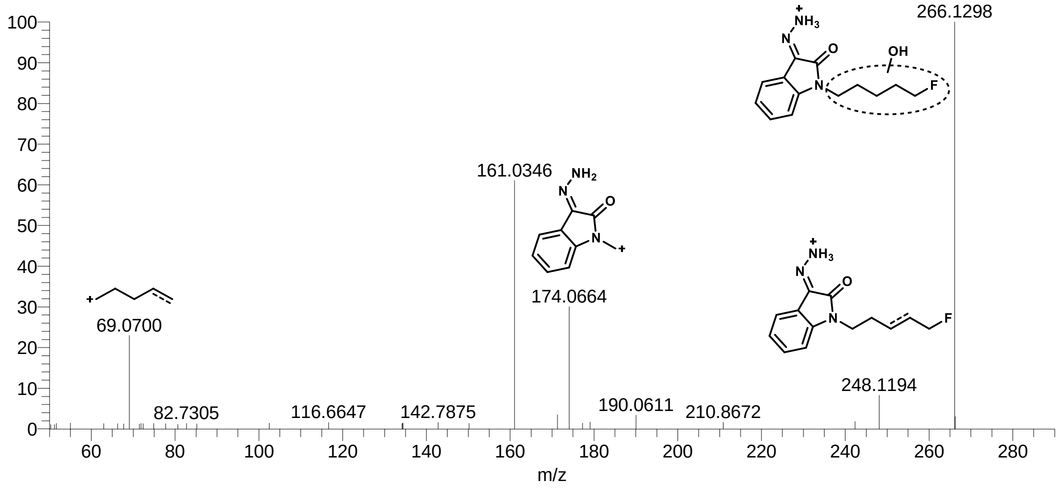

F16

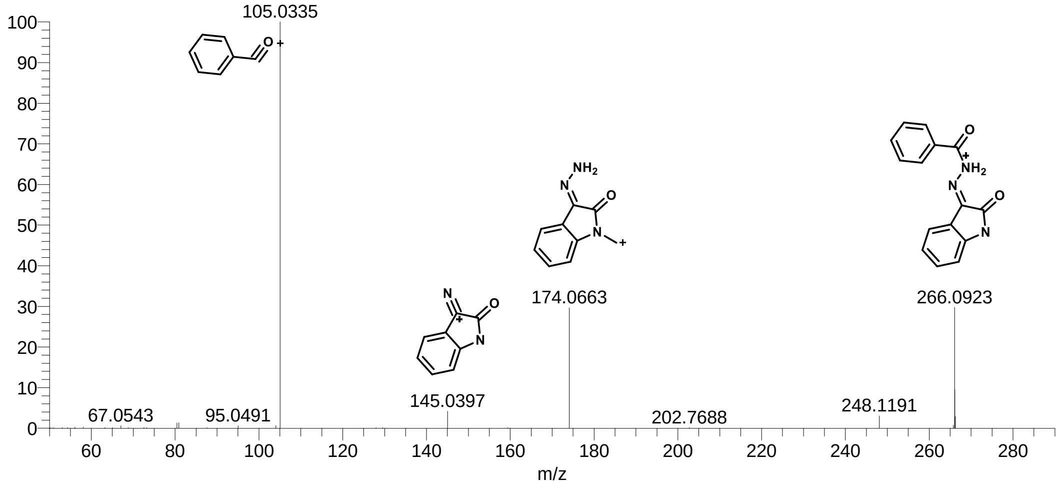

F17-19

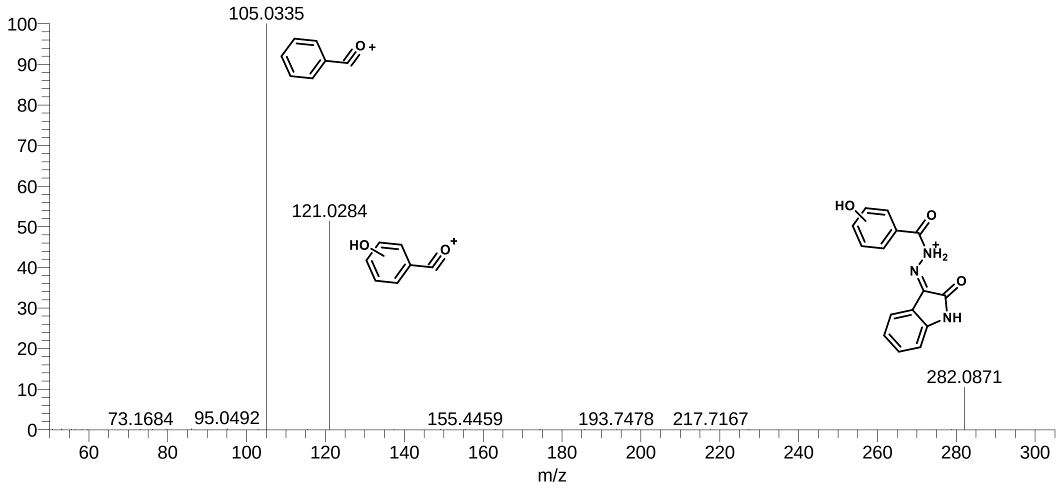
105.0335
121.0284
282.0871
73.1684
95.0492
155.4459
193.7478
217.7167
m/z

F20

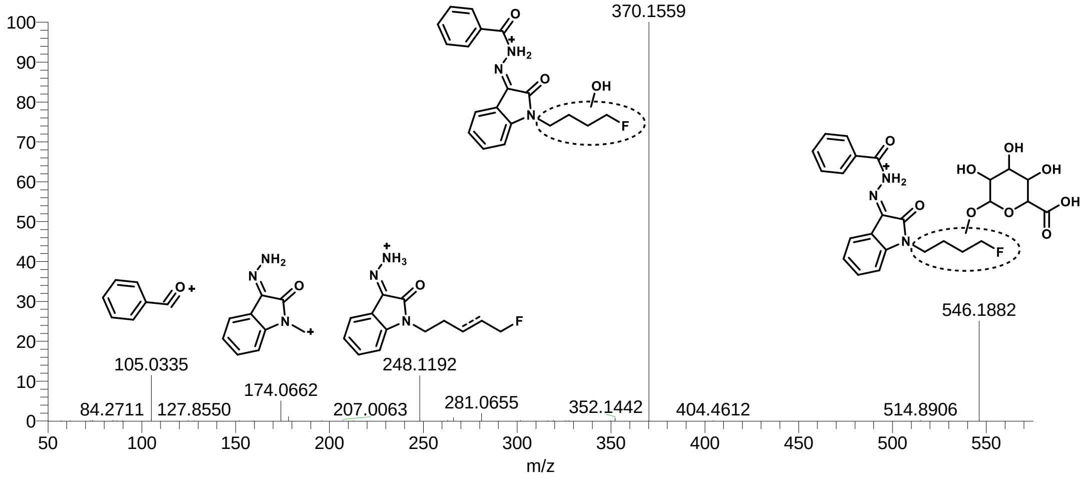
370.1559
546.1882
105.0335
174.0662
248.1192
84.2711
127.8550
207.0063
281.0655
352.1442
404.4612
514.8906
m/z

F21

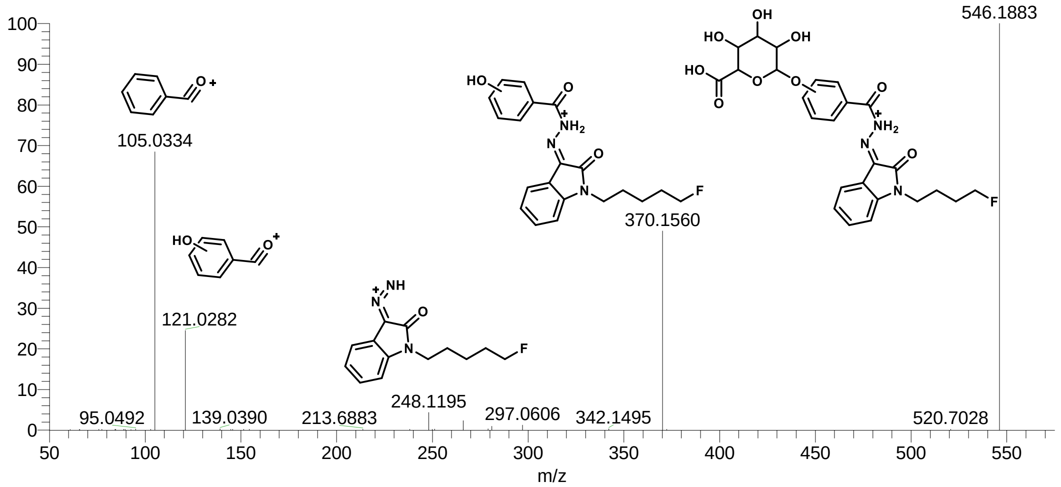
546.1883
105.0334
370.1560
121.0282
95.0492
139.0390
213.6883
248.1195
297.0606
342.1495
520.7028
m/z

F22

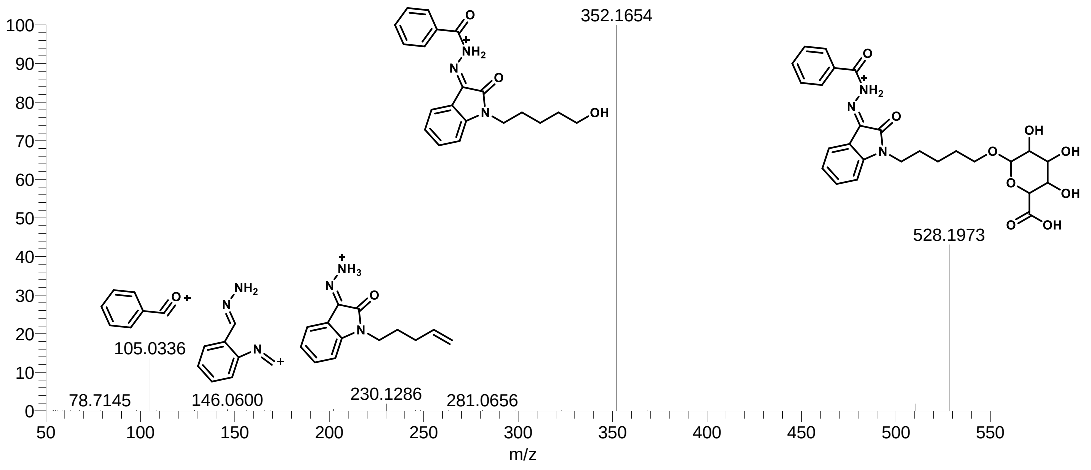
352.1654
528.1973
105.0336
78.7145
146.0600
230.1286
281.0656
m/z

F23

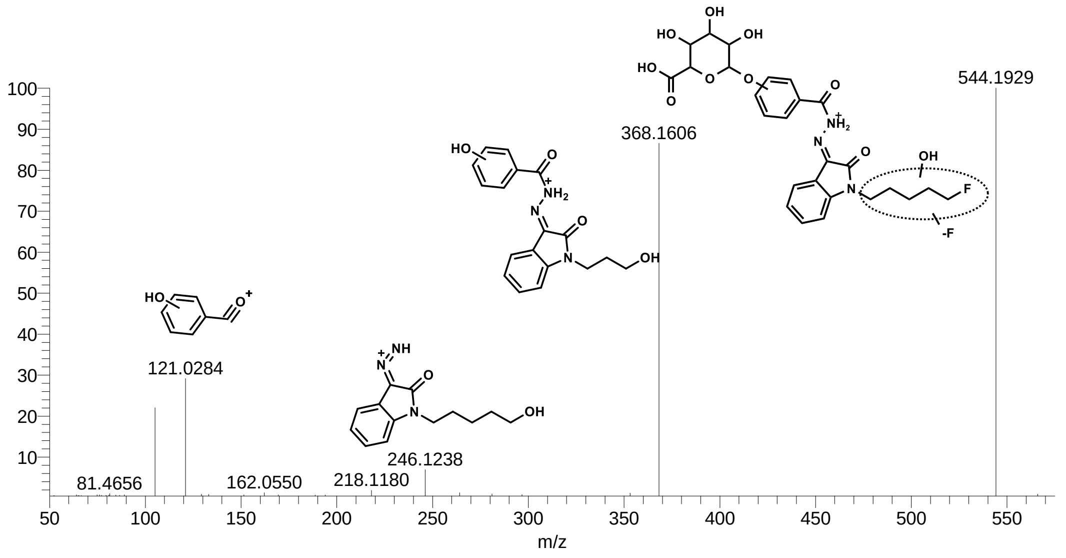
544.1929
368.1606
121.0284
246.1238
81.4656
162.0550
218.1180
m/z

F24

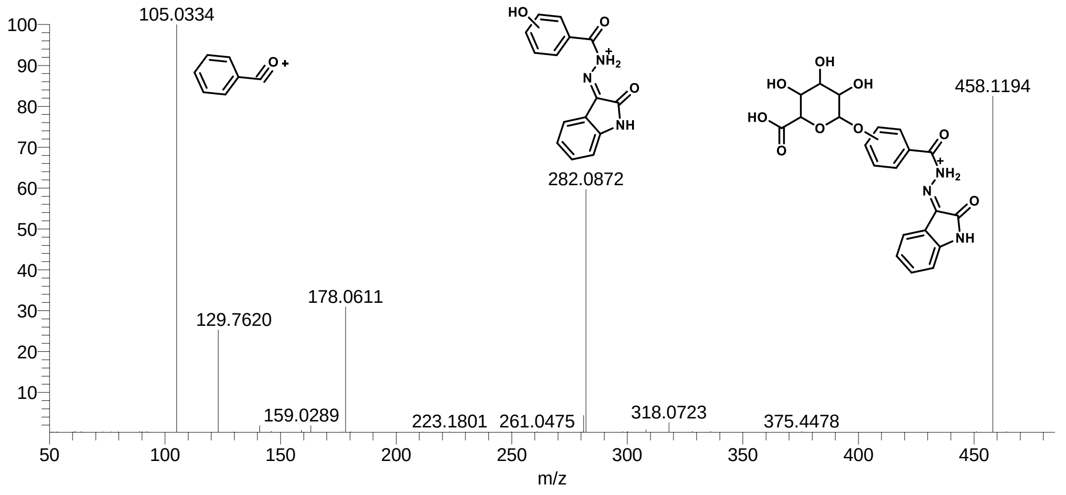
105.0334
458.1194
282.0872
178.0611
129.7620
159.0289
223.1801
261.0475
318.0723
375.4478
m/z
