# Supplementary material for: Study of the Metabolic Profiles of “Indazole-3-Carboxamide” and “Isatin Acyl Hydrazone” (OXIZID) Synthetic Cannabinoids in a Human Liver Microsome Model Using UHPLC-QE Orbitrap MS
Source: Metabolites. 2023 Apr 18;13(4):576. doi: 10.3390/metabo13040576 (PMC10141538; doi:10.3390/metabo13040576)
Supplement: Supplementary file 1 [file metabolites-13-00576-s001.zip › Tables S1-S6.pdf]

Table S1 UHPLC-QE-Orbitrap MS data of ADB-BUTINACA and its metabolites

Name	Biotransformation	Elemental composition [a]	Theoretical mass (m/z)	Measured mass (m/z)	Error (ppm)	RT (min)	Peak areas ($\times 10^7$)
A0	ADB-BUTINACA	$C_{18}H_{27}N_4O_2^+$	331.2129	331.2125	1.21	9.05	549.24
A1	A0+Hydroxylation[A0+O]	$C_{18}H_{27}N_4O_3^+$	347.2078	347.2074	1.15	7.00	125.16
A2	A0+Hydroxylation[A0+O]	$C_{18}H_{27}N_4O_3^+$	347.2078	347.2072	1.73	7.90	90.71
A3	A0+O+Dehydrogenation[A0+O-2H]	$C_{18}H_{25}N_4O_3^+$	345.1921	345.1920	0.29	7.86	30.23
A4	A0+Dihydroxylation[A0+2O]	$C_{18}H_{27}N_4O_4^+$	363.2027	363.2027	0.00	6.11	2.86
A5	A0+Dihydroxylation[A0+2O]	$C_{18}H_{27}N_4O_4^+$	363.2027	363.2030	-0.83	7.07	0.54
A6	A0+2O-2H	$C_{18}H_{25}N_4O_4^+$	361.1870	361.1870	0.00	6.94	0.57
A7	Amido hydrolysis	$C_{18}H_{26}N_4O_3^+$	332.1969	332.1970	-0.30	9.74	60.10
A8	N-Dealkylation	$C_{14}H_{19}N_4O_2^+$	275.1503	275.1496	2.54	6.65	11.13
A9	A0+O+glucuronidation	$C_{24}H_{35}N_4O_9^+$	523.2399	523.2408	-1.72	6.38	64.40

[a]: The elemental composition is for the protonated molecules.

Table S2 UHPLC-QE-Orbitrap MS data of MDMB-4en-PINACA and its metabolites

Name	Biotransformation	Elemental composition [a]	Theoretical mass (m/z)	Measured mass (m/z)	Error (ppm)	RT (min)	Peak areas ($\times 10^7$)
B0	Parent drug MDMB-4en-PINACA	$C_{20}H_{28}N_3O_3^+$	358.2125	358.2122	0.84	11.07	619.44
B1	B0+Hydroxylation[B0+O]	$C_{20}H_{28}N_3O_4^+$	374.2074	374.2072	0.53	8.62	2.25
B2	B0+Hydroxylation[B0+O]	$C_{20}H_{28}N_3O_4^+$	374.2074	374.2076	-0.53	8.99	46.10
B3	B0+Hydroxylation[B0+O]	$C_{20}H_{28}N_3O_4^+$	374.2074	374.2075	-0.27	9.67	11.42
B4	B0+Dihydroxylation[B0+2O]	$C_{20}H_{28}N_3O_5^+$	390.2023	390.2022	0.26	8.16	16.10
B5	B0+Dihydroxylation[B0+2O]	$C_{20}H_{28}N_3O_5^+$	390.2023	390.2023	0.00	8.55	18.43
B6	B0+2O+hydrogenation[B0+2O+2H]	$C_{20}H_{30}N_3O_5^+$	392.2180	392.2176	1.02	7.49	204.19

B7	B0+2O+hydrogenation[B0+2O+2H]	$C_{20}H_{30}N_3O_5^+$	392.2180	392.2177	0.76	7.94	64.15
B8	B0+2O+Dehydrogenation[B0+2O-2H]	$C_{20}H_{26}N_3O_5^+$	388.1867	388.1857	2.58	8.34	2.71
B9	B0+2O+2H+Hydroxylation[B0+3O+2H]	$C_{20}H_{30}N_3O_6^+$	408.2129	408.2127	0.49	6.86	7.79
B10	Ester hydrolysis[B10]	$C_{19}H_{26}N_3O_3^+$	344.1969	344.1967	0.58	9.10	6.90
B11	B10+O	$C_{19}H_{26}N_3O_4^+$	360.1918	360.1916	0.56	7.20	82.71
B12	B10+O+2H	$C_{19}H_{28}N_3O_3^+$	362.2074	362.2070	1.10	7.08	2.58
B13	B10+O-2H	$C_{19}H_{24}N_3O_4^+$	358.1761	358.1759	0.56	7.27	0.29
B14	B10+O-2H	$C_{19}H_{24}N_3O_4^+$	358.1761	358.1759	0.56	7.63	7.79
B15	B10+O-2H	$C_{19}H_{24}N_3O_4^+$	358.1761	358.1766	-1.40	8.75	1.14
B16	B10+2O	$C_{19}H_{26}N_3O_5^+$	376.1867	376.1866	0.27	6.67	11.15
B17	B10+2O	$C_{19}H_{26}N_3O_5^+$	376.1867	376.1868	-0.27	7.32	7.96
B18	B10+2O-2H	$C_{19}H_{24}N_3O_5^+$	374.1710	374.1708	0.53	7.14	0.84
B19	B10+2O-2H	$C_{19}H_{24}N_3O_5^+$	374.1710	374.1708	0.53	7.66	0.77
B20	B10+2O+2H	$C_{19}H_{28}N_3O_5^+$	378.2023	378.2020	0.79	6.87	100.00
B21	Amido hydrolysis+Hydroxylation	$C_{13}H_{15}N_2O_3^+$	247.1077	247.1077	0.00	6.17	1.99
B22	Amido hydrolysis+Hydroxylation	$C_{13}H_{15}N_2O_3^+$	247.1077	247.1077	0.00	7.49	2.46
B23	Amido hydrolysis+Hydroxylation	$C_{13}H_{15}N_2O_3^+$	247.1077	247.1077	0.00	7.95	1.01
B24	N-Dealkylation	$C_{15}H_{20}N_3O_3^+$	290.1499	290.1501	-0.69	8.32	4.62
B25	B0+O+glucuronidation	$C_{26}H_{36}N_3O_{10}^+$	550.2395	550.2411	-2.91	6.24	0.33
B26	B0+O+glucuronidation	$C_{26}H_{36}N_3O_{10}^+$	550.2395	550.2416	-3.82	6.64	2.63
B27	B0+O+glucuronidation	$C_{26}H_{36}N_3O_{10}^+$	550.2395	550.2405	-1.82	7.00	2.56
B28	B10+O+glucuronidation	$C_{25}H_{34}N_3O_9^+$	520.2290	520.2292	-0.38	7.23	3.97
B29	B10+glucuronidation	$C_{26}H_{38}N_3O_{11}^+$	568.2501	568.2500	0.18	6.64	2.63

[a]: The elemental composition is for the protonated molecules.

Table S3 UHPLC-QE-Orbitrap MS data of BZO-HEXOXIZID and its metabolites

Name	Biotransformation	Elemental composition [a]	Theoretical mass (m/z)	Measured mass (m/z)	Error (ppm)	RT (min)	Peak areas ($\times 10^7$)
C0	Parent drug BZO-HEXOXIZID	$C_{21}H_{24}N_3O_2^+$	350.1863	350.1860	0.86	11.67	484.26
C1	C0+Hydroxylation[C0+O]	$C_{21}H_{24}N_3O_3^+$	366.1812	366.1812	0.00	7.62	24.83
C2	C0+Hydroxylation[C0+O]	$C_{21}H_{24}N_3O_3^+$	366.1812	366.1810	0.55	8.68	137.05
C3	C0+Hydroxylation[C0+O]	$C_{21}H_{24}N_3O_3^+$	366.1812	366.1810	0.55	8.91	168.13
C4	C0+Hydroxylation[C0+O]	$C_{21}H_{24}N_3O_3^+$	366.1812	366.1812	0.00	10.40	17.10
C5	C0+O+Dehydrogenation[C0+O-2H]	$C_{21}H_{22}N_3O_3^+$	364.1656	364.1654	0.55	7.89	25.90
C6	C0+O+Dehydrogenation[C0+O-2H]	$C_{21}H_{22}N_3O_3^+$	364.1656	364.1652	1.10	9.08	427.19
C7	C0+O+Dehydrogenation[C0+O-2H]	$C_{21}H_{22}N_3O_3^+$	364.1656	364.1652	1.10	9.45	145.85
C8	C0+Dihydroxylation[C0+2O]	$C_{21}H_{24}N_3O_4^+$	382.1761	382.1761	0.00	6.78	6.99
C9	C0+Dihydroxylation[C0+2O]	$C_{21}H_{24}N_3O_4^+$	382.1761	382.1759	0.52	7.54	85.80
C10	C0+2O+Dehydrogenation[C0+2O-2H]	$C_{21}H_{22}N_3O_4^+$	380.1605	380.1604	0.26	7.21	6.89
C11	C0+2O+Dehydrogenation[C0+2O-2H]	$C_{21}H_{22}N_3O_4^+$	380.1605	380.1602	0.79	8.13	112.44
C12	C0+2O+Dehydrogenation[C0+2O-2H]	$C_{21}H_{22}N_3O_4^+$	380.1605	380.1603	0.53	8.52	49.10
C13	C0+2O+Hydroxylation[C0+3O]	$C_{21}H_{24}N_3O_5^+$	398.1710	398.1711	-0.25	6.87	0.99
C14	C0+2O+Hydroxylation[C0+3O]	$C_{21}H_{24}N_3O_5^+$	398.1710	398.1704	1.51	7.30	0.24
C15	N-Dealkylation	$C_{15}H_{12}N_3O_2^+$	266.0924	266.0918	2.25	6.83	3.87
C16	Amido hydrolysis[C16]	$C_{14}H_{20}N_3O^+$	246.1601	246.1603	-0.81	10.48	0.28
C17	C16+Hydroxylation[C16+O]	$C_{14}H_{20}N_3O_2^+$	262.1550	262.1553	-1.14	6.32	0.81
C18	C16+Hydroxylation[C16+O]	$C_{14}H_{20}N_3O_2^+$	262.1550	262.1552	-0.76	7.18	0.46
C19	C16+Hydroxylation[C16+O]	$C_{14}H_{20}N_3O_2^+$	262.1550	262.1553	-1.14	7.42	1.05
C20	C16+Hydroxylation[C16+O]	$C_{14}H_{20}N_3O_2^+$	262.1550	262.1553	-1.14	8.77	0.13
C21	C16+O+Dehydrogenation[C16+O-2H]	$C_{14}H_{18}N_3O_2^+$	260.1394	260.1393	0.38	6.46	5.03

C22	C16+O+Dehydrogenation[C16+O-2H]	$C_{14}H_{18}N_3O_2^+$	260.1394	260.1393	0.38	7.72	10.16
C23	C16+Dihydrogenation[C16+2O]	$C_{14}H_{20}N_3O_3^+$	278.1499	278.1502	-1.08	5.45	0.23
C24	C16+2O+Dehydrogenation[C16+2O-2H]	$C_{14}H_{18}N_3O_3^+$	276.1343	276.1342	0.36	5.55	0.71
C25	C16+2O+Dehydrogenation[C16+2O-2H]	$C_{14}H_{18}N_3O_3^+$	276.1343	276.1342	0.36	6.37	1.20
C26	C16+2O+Dehydrogenation[C16+2O-2H]	$C_{14}H_{18}N_3O_3^+$	276.1343	276.1345	-0.72	6.70	0.72
C27	C0+O+glucuronidation	$C_{27}H_{32}N_3O_9^+$	542.2133	542.2133	0.00	7.39	4.14
C28	C0+O+glucuronidation	$C_{27}H_{32}N_3O_9^+$	542.2133	542.2133	0.00	7.55	5.99
C29	C0+O+glucuronidation	$C_{27}H_{32}N_3O_9^+$	542.2133	542.2133	0.00	7.81	3.62
C30	C0+O+glucuronidation	$C_{27}H_{32}N_3O_9^+$	542.2133	542.2137	-0.74	8.17	7.92
C31	C0+O+glucuronidation	$C_{27}H_{32}N_3O_9^+$	542.2133	542.2136	-0.55	8.64	18.10
C32	C0+2O+glucuronidation	$C_{27}H_{32}N_3O_{10}^+$	558.2082	558.2086	-0.72	6.80	6.14
C33	C0+2O+glucuronidation	$C_{27}H_{32}N_3O_{10}^+$	558.2082	558.2079	0.54	8.05	1.53
C34	C0+2O-2H+glucuronidation	$C_{27}H_{30}N_3O_{10}^+$	556.1926	556.1929	-0.54	6.94	4.87

[a]: The elemental composition is for the protonated molecules.

Table S4 UHPLC-QE-Orbitrap data of BZO-POXIZID and its metabolites

Name	Biotransformation	Elemental composition [a]	Theoretical mass (m/z)	Measured mass (m/z)	Error (ppm)	RT (min)	Peak areas ($\times10^7$)
D0	BZO-POXIZID	$C_{20}H_{22}N_3O_2^+$	336.1707	336.1703	1.19	11.11	512.08
D1	D0+Hydroxylation[D0+O]	$C_{20}H_{22}N_3O_2^+$	352.1656	352.1652	1.14	7.29	22.14
D2	D0+Hydroxylation[D0+O]	$C_{20}H_{22}N_3O_2^+$	352.1656	352.1655	0.28	8.30	222.30
D3	D0+Hydroxylation[D0+O]	$C_{20}H_{22}N_3O_2^+$	352.1656	352.1653	0.85	8.74	46.61
D4	D0+Hydroxylation[D0+O]	$C_{20}H_{22}N_3O_2^+$	352.1656	352.1654	0.57	9.91	13.03
D5	D0+O+Dehydrogenation[D0+O-2H]	$C_{20}H_{20}N_3O_2^+$	350.1499	350.1495	1.14	7.67	12.81
D6	D0+O+Dehydrogenation[D0+O-2H]	$C_{20}H_{20}N_3O_2^+$	350.1499	350.1496	0.86	8.82	237.51

D7	D0+Dihydroxylation[D0+2O]	$C_{20}H_{22}N_3O_4^+$	368.1605	368.1604	0.27	6.58	4.97
D8	D0+Dihydroxylation[D0+2O]	$C_{20}H_{22}N_3O_4^+$	368.1605	368.1601	1.09	7.30	32.50
D9	D0+2O+Dehydrogenation[D0+2O-2H]	$C_{20}H_{20}N_3O_4^+$	366.1448	366.1445	0.82	7.42	2.66
D10	D0+2O+Dehydrogenation[D0+2O-2H]	$C_{20}H_{20}N_3O_4^+$	366.1448	366.1447	0.27	7.64	12.10
D11	D0+2O+Dehydrogenation[D0+2O-2H]	$C_{20}H_{20}N_3O_4^+$	366.1448	366.1448	0.00	7.79	10.52
D12	N-Dealkylation	$C_{15}H_{12}N_3O_2^+$	266.0924	266.0924	0.00	6.85	4.23
D13	N-Dealkylation+Hydroxylation	$C_{15}H_{12}N_3O_3^+$	282.0873	282.0869	1.42	5.99	0.33
D14	N-Dealkylation+Hydroxylation	$C_{15}H_{12}N_3O_3^+$	282.0873	282.0875	-0.71	6.42	0.20
D15	N-Dealkylation+Hydroxylation	$C_{15}H_{12}N_3O_3^+$	282.0873	282.0874	-0.35	6.95	0.84
D16	Amido hydrolysis[D16]	$C_{13}H_{18}N_3O^+$	232.1444	232.1444	0.00	9.87	0.24
D17	D16+Hydroxylation[D16+O]	$C_{13}H_{18}N_3O_2^+$	248.1394	248.1396	-0.81	5.89	0.59
D18	D16+Hydroxylation[D16+O]	$C_{13}H_{18}N_3O_2^+$	248.1394	248.1394	0.00	6.20	0.36
D19	D16+Hydroxylation[D16+O]	$C_{13}H_{18}N_3O_2^+$	248.1394	248.1396	-0.81	6.70	0.51
D20	D16+Hydroxylation[D16+O]	$C_{13}H_{18}N_3O_2^+$	248.1394	248.1393	0.40	6.95	2.17
D21	D16+O+Dehydrogenation[D0+O-2H]	$C_{13}H_{16}N_3O_2^+$	246.1237	246.1234	1.22	6.09	0.77
D22	D16+O+Dehydrogenation[D0+O-2H]	$C_{13}H_{16}N_3O_2^+$	246.1237	246.1236	0.41	6.57	0.42
D23	D16+O+Dehydrogenation[D0+O-2H]	$C_{13}H_{16}N_3O_2^+$	246.1237	246.1237	0.00	6.95	1.04
D24	D16+O+Dehydrogenation[D0+O-2H]	$C_{13}H_{16}N_3O_2^+$	246.1237	246.1234	1.22	7.35	4.90
D25	D16+O+Dehydrogenation[D0+O-2H]	$C_{13}H_{16}N_3O_2^+$	246.1237	246.1238	-0.41	5.70	0.19
D26	D0+O+glucuronidation	$C_{26}H_{30}N_3O_9^+$	528.1977	528.1968	1.70	7.23	9.63
D27	D0+O+glucuronidation	$C_{26}H_{30}N_3O_9^+$	528.1977	528.1970	1.33	7.79	3.92
D28	D0+O+glucuronidation	$C_{26}H_{30}N_3O_9^+$	528.1977	528.1978	-0.19	8.17	15.30
D29	D0+2O+glucuronidation	$C_{26}H_{30}N_3O_{10}^+$	544.1926	544.1929	-0.55	6.56	4.13
D30	D0+2O+glucuronidation	$C_{26}H_{30}N_3O_{10}^+$	544.1926	544.1922	0.74	7.75	2.67

[a]: The elemental composition is for the protonated molecules.

Table S5 UHPLC-QE-Orbitrap data of BZO-4en-POXIZID and its metabolites

Name	Biotransformation	Elemental composition [a]	Theoretical mass (m/z)	Measured mass (m/z)	Error (ppm)	RT (min)	Peak areas ($\times10^7$)
E0	BZO-4en-POXIZID	$C_{20}H_{20}N_3O_2^+$	334.1550	334.1546	1.20	10.53	659.12
E1	E0+Hydroxylation[E0+O]	$C_{20}H_{20}N_3O_3^+$	350.1499	350.1496	0.86	7.18	7.36
E2	E0+Hydroxylation[E0+O]	$C_{20}H_{20}N_3O_3^+$	350.1499	350.1498	0.29	7.49	6.10
E3	E0+Hydroxylation[E0+O]	$C_{20}H_{20}N_3O_3^+$	350.1499	350.1500	-0.29	8.55	118.45
E4	E0+Hydroxylation[E0+O]	$C_{20}H_{20}N_3O_3^+$	350.1499	350.1496	0.86	8.15	12.40
E5	E0+O+hydrogenation[E0+O+2H]	$C_{20}H_{22}N_3O_3^+$	352.1656	352.1655	0.28	8.29	12.72
E6	E0+O+hydrogenation[E0+O+2H]	$C_{20}H_{22}N_3O_3^+$	352.1656	352.1648	2.27	8.73	1.89
E7	E0+Dihydroxylation[E0+2O]	$C_{20}H_{20}N_3O_4^+$	366.1448	366.1446	0.55	8.21	9.97
E8	E0+Dihydroxylation[E0+2O]	$C_{20}H_{20}N_3O_4^+$	366.1448	366.1448	0.00	7.74	32.30
E9	E0+2O+Dehydrogenation[E0+2O-2H]	$C_{20}H_{18}N_3O_4^+$	364.1292	364.1293	-0.27	8.06	2.96
E10	E0+2O+Hydrogenation[E0+2O+2H]	$C_{20}H_{22}N_3O_4^+$	368.1605	368.1605	0.00	7.19	277.39
E11	E0+2O+2H+Hydtoxylation[E0+3O+2H]	$C_{20}H_{22}N_3O_4^+$	384.1554	384.1554	0.00	6.65	15.90
E12	Amido hydrolysis[E12]	$C_{13}H_{16}N_3O^+$	230.1288	230.1289	-0.43	9.28	1.55
E13	E12+Dehydrogenation[E12-2H]	$C_{13}H_{14}N_3O^+$	228.1131	228.1131	0.00	8.55	8.27
E14	E12+Hydroxylation[E12+O]	$C_{13}H_{16}N_3O_2^+$	246.1237	246.1236	0.41	5.95	1.87
E15	E12+Hydroxylation[E12+O]	$C_{13}H_{16}N_3O_2^+$	246.1237	246.1238	-0.41	6.54	0.97
E16	E12+Hydroxylation[E12+O]	$C_{13}H_{16}N_3O_2^+$	246.1237	246.1236	0.41	7.91	13.80
E17	E12+O+hydrogenation[E12+O+2H]	$C_{13}H_{18}N_3O_2^+$	248.1343	248.1393	-20.15	5.90	0.10
E18	E12+O+hydrogenation[E12+O+2H]	$C_{13}H_{18}N_3O_2^+$	248.1343	248.1396	-21.36	6.17	0.06
E19	E12+O+hydrogenation[E12+O+2H]	$C_{13}H_{18}N_3O_2^+$	248.1343	248.1392	-19.75	6.97	0.57
E20	E12+Dihydroxylation[E12+2O]	$C_{13}H_{16}N_3O_3^+$	262.1186	262.1186	0.00	5.98	1.55

E21	E12+Dihydroxylation[E12+2O]	$C_{13}H_{16}N_3O_3^+$	262.1186	262.1186	0.00	6.65	0.65
E22	E12+2O+Hydrogenation[E12+2O+2H]	$C_{13}H_{18}N_3O_3^+$	264.1343	264.1342	0.38	5.10	1.02
E23	E12+2O+Hydrogenation[E12+2O+2H]	$C_{13}H_{18}N_3O_3^+$	264.1343	264.1340	1.14	5.94	3.66
E24	N-Dealkylation	$C_{15}H_{12}N_3O_2^+$	266.0924	266.0924	0.00	6.85	1.59
E25	E0+O+2H+glucuronidation	$C_{26}H_{30}N_3O_9^+$	528.1977	528.1962	2.84	7.18	0.27
E26	E0+2O+2H+glucuronidation	$C_{26}H_{30}N_3O_{10}^+$	544.1926	544.1915	2.02	6.63	1.33

[a]: The elemental composition is for the protonated molecules.

Table S6 UHPLC-QE-Orbitrap data of 5F-BZO-POXIZID and its metabolites

Name	Biotransformation	Elemental composition [a]	Theoretical mass (m/z)	Measured mass (m/z)	Error (ppm)	RT (min)	Peak areas ($\times 10^7$)
F0	5F-BZO-POXIZID	$C_{20}H_{21}FN_3O_2^+$	354.1612	354.1608	1.13	10.01	553.71
F1	F0+Hydroxylation[F0+O]	$C_{20}H_{21}FN_3O_3^+$	370.1561	370.1559	0.54	7.55	11.30
F2	F0+Hydroxylation[F0+O]	$C_{20}H_{21}FN_3O_3^+$	370.1561	370.1558	0.81	8.43	149.01
F3	F0+O+Dehydrogenation[F0+O-2H]	$C_{20}H_{19}FN_3O_3^+$	368.1405	368.1404	0.27	7.99	1.17
F4	F0+O+Dehydrogenation[F0+O-2H]	$C_{20}H_{19}FN_3O_3^+$	368.1405	368.1418	-3.53	8.58	0.93
F5	F0+O+Dehydrogenation[F0+O-2H]	$C_{20}H_{19}FN_3O_3^+$	368.1405	368.1403	0.54	8.97	7.21
F6	F0+Defluorination+Hydroxylation[F0-F+O]	$C_{20}H_{22}FN_3O_3^+$	352.1656	352.1652	1.14	8.26	446.13
F7	F0+Defluorination+Hydroxylation[F0-F+O]	$C_{20}H_{22}FN_3O_3^+$	352.1656	352.1653	0.85	7.31	21.90
F8	F0-F+O+Dehydrogenation[F0-F+2O]	$C_{20}H_{22}FN_3O_4^+$	368.1605	368.1602	0.81	6.52	1.82
F9	F0-F+O+Dehydrogenation[F0-F+2O]	$C_{20}H_{22}FN_3O_4^+$	368.1605	368.1607	-0.54	7.71	20.79
F10	F0-F+O+Dehydrogenation[F0-F+2O]	$C_{20}H_{22}FN_3O_4^+$	368.1605	368.1602	0.81	7.45	7.53
F11	F0-F+2O+Dehydrogenation[F0-F+2O-2H]	$C_{20}H_{20}FN_3O_4^+$	366.1448	366.1444	1.09	8.19	171.18
F12	Amido hydrolysis[F11]	$C_{13}H_{17}FN_3O^+$	250.1350	250.1349	0.40	7.35	4.45
F13	F11+Hydroxylation[F11+O]	$C_{13}H_{17}FN_3O_2^+$	266.1299	266.1298	0.38	5.12	2.04

F14	F11+Hydroxylation[F11+O]	$C_{13}H_{17}FN_3O_2^+$	266.1299	266.1295	1.50	5.58	0.22
F15	F11+Hydroxylation[F11+O]	$C_{13}H_{17}FN_3O_2^+$	266.1299	266.1301	-0.75	5.87	0.29
F16	N-Dealkylation[F15]	$C_{15}H_{12}N_3O_2^+$	266.0924	266.0923	0.38	6.84	9.88
F17	N-Dealkylation+Hydroxylation[F15+O]	$C_{15}H_{12}N_3O_3^+$	282.0873	282.0869	1.42	5.97	1.11
F18	N-Dealkylation+Hydroxylation[F15+O]	$C_{15}H_{12}N_3O_3^+$	282.0873	282.0870	1.06	6.44	0.55
F19	N-Dealkylation+Hydroxylation[F15+O]	$C_{15}H_{12}N_3O_3^+$	282.0873	282.0873	0.00	7.22	11.12
F20	F0+O+glucuronidation	$C_{26}H_{29}FN_3O_9^+$	546.1882	546.1882	0.00	7.12	8.78
F21	F0+O+glucuronidation	$C_{26}H_{29}FN_3O_9^+$	546.1882	546.1884	-0.37	7.57	22.08
F22	F0-F+O+glucuronidation	$C_{26}H_{30}N_3O_9^+$	528.1977	528.1973	0.76	7.15	9.13
F23	F0-F+2O+glucuronidation	$C_{26}H_{30}N_3O_{10}^+$	544.1926	544.1929	-0.55	6.46	6.17
F24	F15+O+glucuronidation	$C_{21}H_{20}N_3O_9^+$	458.1194	458.1194	0.00	5.95	5.18

[a]: The elemental composition is for the protonated molecules.
